# Detection of mosaic and population-level structural variants with Sniffles2

**DOI:** 10.1038/s41587-023-02024-y

**Published:** 2024-01-02

**Authors:** Moritz Smolka, Luis F. Paulin, Christopher M. Grochowski, Dominic W. Horner, Medhat Mahmoud, Sairam Behera, Ester Kalef-Ezra, Mira Gandhi, Karl Hong, Davut Pehlivan, Sonja W. Scholz, Claudia M. B. Carvalho, Christos Proukakis, Fritz J. Sedlazeck

**Affiliations:** 1 Human Genome Sequencing Center Baylor College of Medicine, Houston, TX USA; 2 Department of Molecular and Human Genetics, Baylor College of Medicine, Houston, TX USA; 3 Department of Clinical and Movement Neurosciences, Royal Free Campus, Queen Square Institute of Neurology, University College London, London, UK; 4 Aligning Science Across Parkinson’s (ASAP) Collaborative Research Network, Chevy Chase, MD USA; 5 Pacific Northwest Research Institute (PNRI), Seattle, WA USA; 6 Bionano Genomics, San Diego, CA USA; 7 Division of Neurology and Developmental Neuroscience, Department of Pediatrics, Baylor College of Medicine, Houston, TX USA; 8 Neurodegenerative Diseases Research Unit, National Institute of Neurological Disorders and Stroke, Bethesda, MD USA; 9 Department of Neurology, Johns Hopkins University Medical Center, Baltimore, MD USA; 10 Department of Computer Science, Rice University, Houston, TX USA

**Keywords:** Genome informatics, Genetics, Cancer, Software

## Abstract

Calling structural variations (SVs) is technically challenging, but using long reads remains the most accurate way to identify complex genomic alterations. Here we present Sniffles2, which improves over current methods by implementing a repeat aware clustering coupled with a fast consensus sequence and coverage-adaptive filtering. Sniffles2 is 11.8 times faster and 29% more accurate than state-of-the-art SV callers across different coverages (5–50×), sequencing technologies (ONT and HiFi) and SV types. Furthermore, Sniffles2 solves the problem of family-level to population-level SV calling to produce fully genotyped VCF files. Across 11 probands, we accurately identified causative SVs around *MECP2*, including highly complex alleles with three overlapping SVs. Sniffles2 also enables the detection of mosaic SVs in bulk long-read data. As a result, we identified multiple mosaic SVs in brain tissue from a patient with multiple system atrophy. The identified SV showed a remarkable diversity within the cingulate cortex, impacting both genes involved in neuron function and repetitive elements.

## Abstract (web summary)

Sniffles2 detects mosaic structural variation from bulk long-read sequencing data.

## Main

The role and biological impact of structural variations (SVs) have become evident^1,2^. SVs are loosely defined as 50-base pairs (bp) or larger genomic alterations that fall into five types (insertions, inversions, deletions, duplications and translocations) or a combination of these types^1^. Given that this type of variant impacts the most number of nucleotides in a genome, it is not surprising that evidence is mounting regarding their importance across all categories of life. This starts, for example, with speciation events^3^ and impacts plants^4,5^ but goes further across human diseases (Mendelian^6,7^ and complex diseases^8–10^) to cancer development^11–13^. Despite the importance of SVs, it is still challenging to detect germline and somatic SVs or even to robustly identify de novo SVs^14–16^. The least often studied and, thus, most challenging SVs are insertions (that is, novel sequences) that, as many studies showed, amount to half of all SVs found in a human genome^17–19^. The latter can be recovered either by long-read mapping methods or by de novo assemblies, followed by a genomic alignment^1,20^.

Long-read sequencing has come a long way over the past years from a novelty to a population/production-scale mechanism to study SVs^21,22^. The error rate of Oxford Nanopore Technologies (ONT) and PacBio HiFi are both ever decreasing^23,24^. Indeed, several studies have now started to sequence larger and larger datasets or even medical applications using PacBio HiFi or ONT^21,25^. This trend started with GENCODE^22^ but is ever increasing to other projects (for example, All of Us initiative^26^ and the Center for Alzheimer’s and Related Dementias (CARD)^27^) and is currently peaking in the M42 endeavor to sequence multiple hundreds of thousands of genomes. This also requires more efficient software to not just detect SVs but also to merge and produce a fully genotyped variant call format (VCF) file^28,29^. The improved degrees of error and lower cost for long reads are also starting to promote applications in medical or clinical space^30,31^. This is needed as several genes or regions of the genome remain a ‘dark matter’^20,32^. Most of these genes (∼70%) can be assessed using long-read technologies, but several challenges remain^32^.

Furthermore, there are more complex SVs beyond simple deletions, duplications, inversions, insertions and translocations that can lead to a Mendelian disease^6^. The genomic locus including the dosage-sensitive gene *MECP2* at Xq28 is particularly susceptible to such genomic instability due to nearby inverted and direct orientation low-copy repeats (LCRs)^33–35^. The protein encoded by *MECP2*, methyl-CpG binding protein 2 (MeCP2), is critical for brain function by acting as an epigenetic regulator^36^. Copy number variation spanning this gene causes *MECP2* duplication syndrome (MDS) (Mendelian Inheritance in Man (MIM): 300260) with 100% penetrance in males^37^. The most prevalent clinical features of MDS are infantile hypotonia, developmental delay, intellectual disability, frequent respiratory infections and refractory epilepsy^38^. One of the frequent complex allele presentations is constituted by an inverted triplication flanked by duplications (DUP-TRP/INV-DUP). This allele is generated by a given pair of inverted LCRs telomeric to *MECP2* being responsible for 20–30% of the MDS cases^6^, a fraction of which will lead to a more severe clinical phenotype. When generated, this structure includes two breakpoint junctions (Jct) connecting the end of the duplication to the end of the triplication (Jct1) and the beginning of the triplication to the beginning of the duplication (Jct2). Given the presence of two breakpoint junctions in *cis*, the involvement of LCRs and the size of such events (often >500 kilobases (kb)), we lack the ability not only to detect this structure solely using long-read sequencing data but also to describe it after the VCF specification. Part of the complexity originates as the reads themselves only partially indicate the allele—for example, highlighting a shorter inversion^28^.

In addition to complex variants, multiple studies have shown that there are mosaic or low-frequency SVs that are likely causal across neurological diseases or other diseases^9^. As an example, single-cell studies show that there can be variable copy number variants (CNVs) across multiple cells in the brain^9^. However, their true frequency is unknown, with around 12% of healthy cortical neurons having megabase (Mb)-scale CNVs^39^. A possible role in neurodegenerative disease^40^ has not been adequately explored. In synucleinopathies, which include Parkinson’s disease and multiple system atrophy^41^ (MSA), somatic CNVs of the highly relevant *SNCA* gene have been reported^42–44^, and single-cell whole-genome sequencing (WGS) in MSA has shown Mb-scale CNVs in approximately 30% of cells^43^. Still, these CNVs studies lack resolution as breakpoints are defined within ± multiple kbp, and only very large ∼1-Mb CNV events are reported^39,45,46^. So far, an identification of complex SVs arising in neurodevelopment has only been possible with WGS of clonally expanded precursors^9,43^. It has, thus, been difficult to identify the underlying alleles even for large, already reported CNVs along the human genome.

Here we present Sniffles2, a redesign of Sniffles, with improved accuracy, higher speed and features that address the problem of population-scale SV calling for long reads. This is needed across tumor/normal comparison over family (for example, Mendelian) studies but also in larger studies deciphering rare alleles across a population or cohort. In addition, Sniffles2 enables the detection of low-frequency SVs across datasets, which facilitates detection of somatic SVs and mosaicism studies and opens the field of cell heterogeneity for long-read applications. We first highlight the performance of Sniffles2 compared to other SV callers over multiple benchmark sets, and, then, we further investigate how the new population or family mode for SV calling improves the accuracy and performance across Mendelian disease probands with ONT. Here we showcase the boundaries of long-read SV calling by assessing highly complex SVs around *MECP2*. Lastly, we investigate the abiliity of Sniffles2 to identify low-frequency/mosaic SVs across an MSA brain sample and compare its performance to Illumina sequencing and Bionano optical genome mapping (OGM). Overall, Sniffles2 pushes the boundaries of long-read-based SV calling and, thus, demonstrates the utility of such an approach further than any existing approach. Sniffles2 remains open source (MIT license) and is available at https://github.com/fritzsedlazeck/Sniffles.

## Results

### Accurate detection of structural variations at scale

**Fig. 1:**
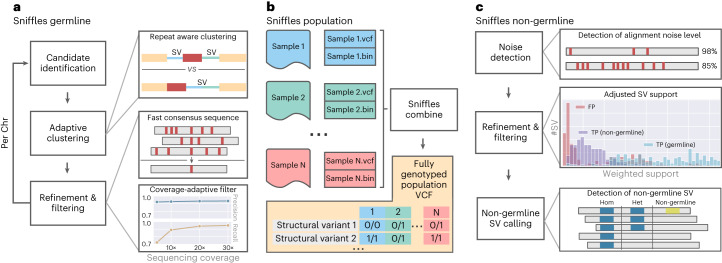
Overview of Sniffles2. **a**, For Sniffles2, we implemented a repeat aware clustering coupled with a fast consensus sequence and coverage-adaptive filtering to improve accuracy of the germline SV calls. **b**, One key limitation of current SV calling is the generation of fully genotyped population VCF. Sniffles2 implements a concept similar to a gVCF file where single-sample calling is done only once, which reduces runtime multiple-fold. **c**, Mosaic SV detection is enabled by improved detection and filtering of low VAF SVs (by default, 5–20%) across a bulk sample. This is enabled over additional noise detection methodology as well as refinement and filtering approaches that we developed.

Sniffles2 is a complete redesign and extension of the SV caller Sniffles^28^. Figure 1 gives an overview of its main components. Sniffles2 now implements repeat aware clustering to improve germline SV calling (Fig. 1a) and further enables family and population SV calling at scale and ease (Fig. 1b) and implements methods to identify mosaic SVs (Fig. 1c). A detailed description of Sniffles2 can be found in the Methods section.

Figure 1a shows a summary of the most important steps applied by Sniffles2 to identify germline SVs. In brief, we use a fast yet high-resolution clustering approach, which identifies SVs in three key steps. First, putative SV events are extracted from read alignments (split reads and inline insertion or deletion events) and allocated to high-resolution bins (default, 100 bp) based on their genomic coordinates and putative SV type. Second, neighboring SV candidate bins are subsequently merged based on a standard deviation measure of SV starting positions within each growing bin. Through the use of optional tandem repeat annotations, Sniffles2 dynamically adapts clustering parameters during SV calling, allowing it to detect single SVs that have been scattered as a result of alignment artifacts. Finally, identified clusters are separately re-analyzed and split based on putative SV length. Final SV candidates are subjected to quality control based on read support, breakpoint variance and expected coverage changes.

**Fig. 2:**
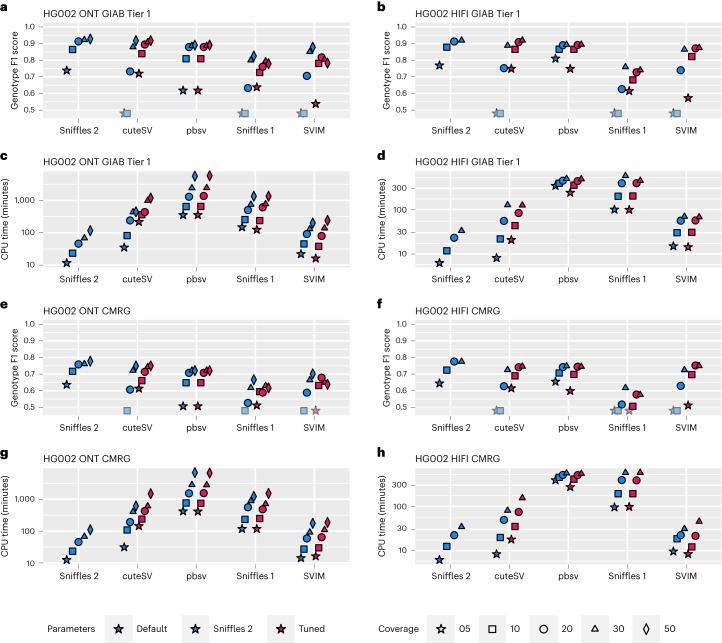
Performance assessment of Sniffles2 based on GIAB. Performance metrics for correctly identifying and genotyping SVs across ONT (left) and PacBio HiFi (right). All details are presented in Supplementary Table 1. For **a**,**b**,**e**,**f**, the shaded symbols mean that the Genotype F1 score was lower than 0.5. **a**,**b**, Comparison across Tier1 GIAB genome-wide SV (Genotype F1 score on the *y* axis; higher is better) across different coverages (symbols) and SV caller (*x* axis) for default and maximum sensitivity parameters (blue and red (Tuned), respectively). **c**,**d**, Runtime comparison across Tier1 GIAB genome-wide SV (CPU minutes on the *y* axis; lower is better) across different coverages (symbols) and SV caller (*x* axis) for default and maximum sensitivity parameters (blue and red (Tuned), respectively). **e**,**f**, Comparison across GIAB challenging medical gene (CMRG) benchmark for SV (Genotype F1 score on the *y* axis; higher is better) across different coverages (symbols) and SV caller (*x* axis) for default and maximum sensitivity parameters. **g**,**h**, Runtime comparison across GIAB CRMG benchmark for SV (CPU minutes on the *y* axis; lower is better) across different coverages (symbols) and SV caller (*x* axis) for default and maximum sensitivity parameters.

We assessed the performance of Sniffles2 (version 2.2) with respect to Sniffles^28^ (version 1.12), cuteSV^47^ (version 1.0.11), PBSV^48^ (version 2.6.2) and SVIM^49^ (version 1.4.2) using Truvari^50^ (version 2.1) and the Genome in a Bottle (GIAB) recommended parameters^51^. Figure 2 shows the results across different GIAB benchmarks (see Supplementary Table 1 for details). Across the tests, we show that Sniffles2 outperforms the other methods in speed and accuracy based on various conditions. Supplementary Section 1 gives additional details (see Supplementary Tables 1–8 and Supplementary Figs. 1–3 for even more details).

Given all these comparisons across different ethnicities (HG002 being Eastern European Ashkenazi Jewish ancestry, HG01243 being Puerto Rican in Puerto Rico, HG02055 being African Caribbean in Barbados and HG02080 being Kinh in Ho Chi Minh City, Vietnam), coverage levels (5–50×) and sequencing technologies (HiFi and ONT), we conclude that Sniffles2 improves the detection of SVs in terms of accuracy and speed compared to other state-of-the-art methods.

### Enabling population-wide studies of the impact of complex SV

**Fig. 3:**
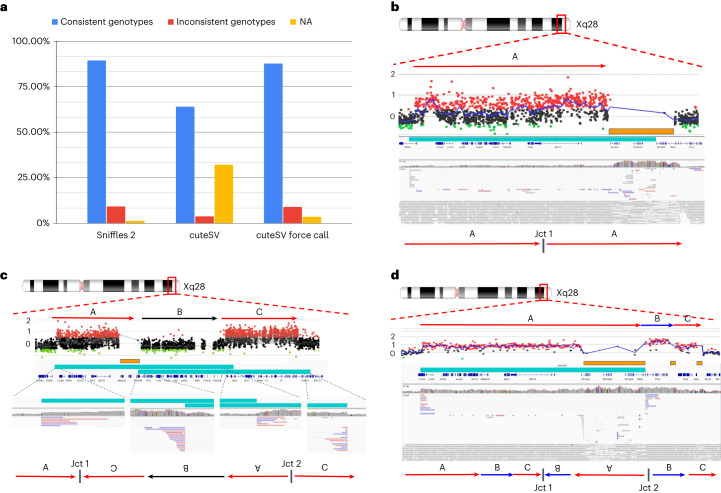
Sniffles2 population approach and application to Mendelian disease. **a**, Comparison of the proportion of consistent, inconsistent and uninformative (NA) genotypes across HG002/3/4 for Sniffles2 population merge and cuteSV. cuteSV with genotyping takes more than 6.24× the time. **b**–**d**, Three examples of SVs detected by Sniffles2 in Mendelian disorders in probands. Chromosomal position is shown in the top part (Xq28), followed by the arrows that represent a specific loci. Next is shown aCGH data dots that represent genomic positions being assayed. Black dots represent a log_2_ ratio between −0.35 and 0.35; red dots represent a log ratio above 0.35; and green dots represent a ratio below −0.35. Consistent (at least three consecutive probes) log_2_ ratios above 0.35 represent a region of copy number gain and below −0.35 represent copy number loss. In orange, we show SegDups, and, in teal, we show the SV called by Sniffles2. IGV screenshot and fully resolved events are shown in the lower part of each example. **b**, Tandem duplication that was fully resolved by Sniffles2 in one of the patients (BH14233_1). Sniffles2 was able to identify and map the junction of the duplication within a segmental duplication region where array data does not provide information. **c**, Detailed aCGH view of a complex duplication-normal-duplication (DUP-NML-DUP) structure in sample BH13947_1 with breakpoints within SegDup or LCR region (orange bar) where Sniffles2 is indicating two overlapping inversions in IGV (teal bars) forming Jct1 and Jct2. Bottom arrows indicate the possible DUP-NML-INV/DUP haplotype structure containing Jct1 and Jct2. **d**, Sample BH15700_1 shows a complex duplication-triplication-duplication structure as highlighted in aCGH data with SegDups and LCRs highlighted (orange bars). Sniffles2 identifies the inversion breakpoint at Jct2 (teal bar) but cannot fully resolve the entire allele including Jct1 as it is also not possible to be reported in the VCF standard. Red arrows indicate duplicated regions, and blue arrows show triplicated portions. One possible haplotype structure for a DUP-TRP/INV-DUP is shown with the triplication and initial duplication being inverted, forming Jct1 and Jct2 (ref. ^34^).

**Extended Data Fig. 1:**
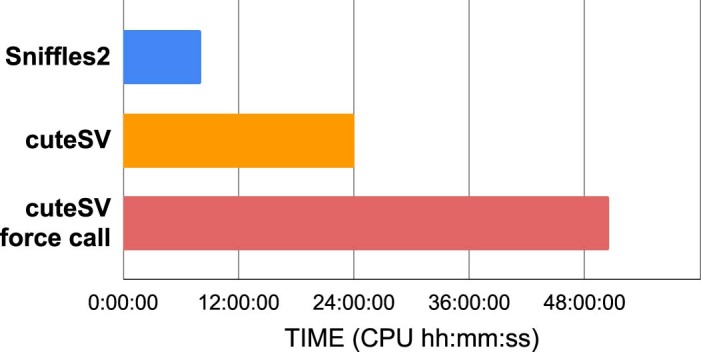
Performance of Sniffles2 population merge. Here we show the total time used by each approach, which includes the SV calling for each member of a family trio, merging SV into a single VCF file, and in the case of cuteSV force call, re-genotyping each sample followed by a second merge. See Supplementary Table 10 for details.

Over the past years, an uptake of ever larger studies using long reads is foreshadowing a trend in genomics to use long reads more often than ever^21^. To promote this, Sniffles2 is fast and efficient but further implements a strategy to obtain a fully genotyped population VCF. Traditionally this is a multi-stage process of calling, merging, genotyping and re-merging^21,52,53^. This is clearly inefficient as the BAM/CRAM alignment files need to be assessed twice. Even so, this process can be achieved only by using a few of the existing methods (SVJedi^54^, Sniffles^28^ and cuteSV^47^). The Sniffles2 strategy requires only an initial calling and merging to obtain a fully genotyped population-level VCF. Figure 1b illustrates the principle. The calling can be done independently where each sample produces a single VCF file accompanied by a binary file that serializes every single candidate SV that has even a single read support. Next, each binary file per sample is provided as a list to Sniffles2 merge, which combines the SV across the samples and fills the missing information using the binary files per sample. This process is extremely efficient as it scales linearly with the number of samples and allows the samples to be analyzed in parallel and independently of each other (Supplementary Table 9 and Supplementary Fig. 4). In addition, it solves the ‘n + 1’ problem, by allowing the inclusion of further samples in the future by only re-doing the merge step, instead of re-genotyping across each BAM file. To assess the validity of this, we measured the Mendelian inconsistency rate using a family trio (Methods)^55^. For Sniffles2, we obtained a Mendelian inconsistency rate of 9.13% with a low rate of missing genotype of 1.29% for SVs with less than 5× coverage (default parameter) (Fig. 3a). In comparison, cuteSV with a simple merge (SURVIVOR^56^) presented a Mendelian inconsistency of 3.74%, with a much higher missingness of 32.20%. When we apply a re-genotyping and re-merging of the cuteSV results, we obtain a Mendelian inconsistency rate of 8.88% with almost three times higher missingness of 3.45% when compared to Sniffles2. Furthermore, the cuteSV approach took more than 50 h of CPU time (Supplementary Table 10 and Extended Data Fig. 1) in contrast to only 8 h of CPU time for Sniffles2, thus rendering it impractical for larger cohorts. As a stress test, we merged from three to 777 samples, which consisted of repeating up to 259 times the HG002 family trio. This took a little more than 11 h of CPU time using Sniffles2 (11:16:18; Supplementary Table 9).

Next, we applied this population/family approach of Sniffles2 across 31 ONT datasets that represented cases of Mendelian disorders in probands (seven complete trios, one duo and eight only probands). The merge was completed in 28 CPU minutes, and we measured an average of 3.89% Mendelian inconsistency rate and 1.11% of missingness (Methods, Supplementary Table 11 and Supplementary Fig. 5). The probands for sequencing were selected based on a Mendelian disease that often is caused by SVs impacting *MECP2* at the Xq28 locus. As described in the introduction, this is a severe neurodevelopmental disorder that is often caused by extreme complex alleles in this region. We were interested if Sniffles2, together with ONT data, can resolve the breakpoints, which were not always solvable using array data, and if we were able to fully explain the entire allele or just partially solve the junctions. To address this, we filtered SVs based on ChrX together with their size (10 kb) and filtered for SVs only being de novo or inherited from the mother.

Within this cohort, Sniffles2 is able to achieve a high rate of detection across junctions but sometimes struggles to recapitulate the entire allele that contains complex SVs. Table 1 shows the details per proband. In samples harboring a tandem duplication, Sniffles2 was able to properly detect the allele and fully resolve its architecture. In our cohort, these duplications span the dosage-sensitive gene (*MECP2*) and form a single breakpoint junction (Jct1), confirming a tandem duplication structure. As highlighted in sample BH14233_1, although aCGH broadly defines the genomic interval of the duplicated region, Sniffles2 is able to properly give positional context of genomic fragment defining at nucleotide-level resolution to be a tandem duplication on the allele even though the end of the duplication is within a segmental duplication region (orange bar) (Fig. 3b). Note that the presence of the segmental duplication caused the SV to be tagged with a STDEV_LEN filter. This indicates non-agreement on the precise start of the SV given the repetitive nature of the region.

**Table 1:** Table across all the probands assessed here and highlighting in bold which junctions could be resolved using Sniffles2

ID	Sex	Inheritance	Pathogenic CGR	Coordinates CGR (aCGH)					Coordinates (Sniffles2)					
				CNV	Chr	Start	SegDUP	End	SegDUP	SV	Chr	Start	End	Filter
**BH14233_1**	M	Maternal	Tandem duplication	DUP	X	**153084841**	-	**153414342**	Yes	DUP	X	**153084620**	**153483892**	**STDEV_LEN**
**BH13948_1**	M	Maternal	Tandem duplication	DUP	X	**152877325**	-	**153414342**	Yes	DUP	X	**152808716**	**153487348**	**PASS**
**BH15642_1**	F	De novo	Tandem duplication	DUP	X	**153289589**	-	**153399165**	-	DUP	X	**153289208**	**153386550**	**PASS**
**BH13947_1**	M	Maternal	DUP-NML-INV/DUP	DUP1	X	**153106533**	-	**153414342**	Yes	INV	X	**153106249**	**153937616**	**PASS**
				DUP2	X	**153938964**	-	**154293950**	-	INV	X	**153492860**	**154294604**	**PASS**
**BH15700_1**	M	Maternal	DUP-TRP/INV-DUP	DUP1	X	**153131406**	-	153409337	-	INV	X	**153131086**	**153520844**	**PASS**
				TRP	X	**153523170**	Yes	153565901	Yes					
				DUP2	X	153575989	-	153623000	Yes					
**BH15701_1**	M	Maternal	DUP-TRP/INV-DUP	DUP1	X	**153189181**	-	153420198	Yes	INV	X	**153188685**	**153499734**	**PASS**
				TRP	X	**153505485**	Yes	153565901	Yes					
				DUP2	X	153575989	-	153623000	Yes					
**BH15646_1**	M	Maternal	Terminal DUP/ recombinant chromosome	DUP	X	**147326287**	-	Telomere	-	INV	X	**1406919**	**147326058**	**PASS**
				DEL	X	Telomere	-	**1405994**	-					
**BH15692_1**	M	De novo	Terminal DUP/Translocation Y	DUP	X	**151905254**	Yes	Telomere	-	BND	X	**151904176**	**N]Y:23243741]**	**PASS**
				DUP	Y	**23243948**	-	23655166	Yes					
				DEL	Y	24095954	Yes	Telomere	-	BND	X	**23243742**	**]X:151904175]N**	**PASS**
**BH15696_1**	M	De novo	Terminal DUP/translocation Y	DUP1	X	**148351663**	-	148384182	-	BND	X	**148351430**	**]Y:28389311]N**	**PASS**
				DUP2	X	**148706667**	-	Telomere		BND	X	**148384577**	**[Y:25210061[N**	**PASS**
				DEL	Y	**28458870**	Yes	Telomere		BND	X	**148705972**	**N]Y:25654822]**	**PASS**
**BH14229_1**	M	Maternal	Terminal duplication /unknown structure	DUP	X	**151893933**	Yes	Telomere	-	INV	X	**151919987**	**155251615**	**PASS**
**BH13949_1**	M	Maternal	Terminal DUP/ unknown structure	DUP1	X	**144057799**	-	144066387	-	DUP	X	**144056099**	**150063756**	**PASS**
				TRP	X	144067901	-	144101282	-	INV	X	**144068403**	**150063449**	**PASS**
				DUP2	X	144101282	-	Telomere	-					

Variants assessed in all the probands including junctions which could be resolved using Sniffles2. Results from BH14233_1, BH13947_1, BH15646_1 and BH15700_1 are discussed in the main text.

A portion of the inversions that Sniffles2 was able to detect were not simple genomic inversions but, instead, part of more complex structures that could not be fully resolved using current bioinformatic tools. A more complex allele was detected in sample BH13947_1, which consists of a duplication-normal-duplication (DUP-NML-INV/DUP) with breakpoints spanning segmental duplications (SegDups) (Fig. 3c). Here, Sniffles2 indicates two overlapping inversions that form junctions 1 and 2 (Jct1 and Jct2), generating a DUP-NML-INV/DUP structure.

**Extended Data Fig. 2:**
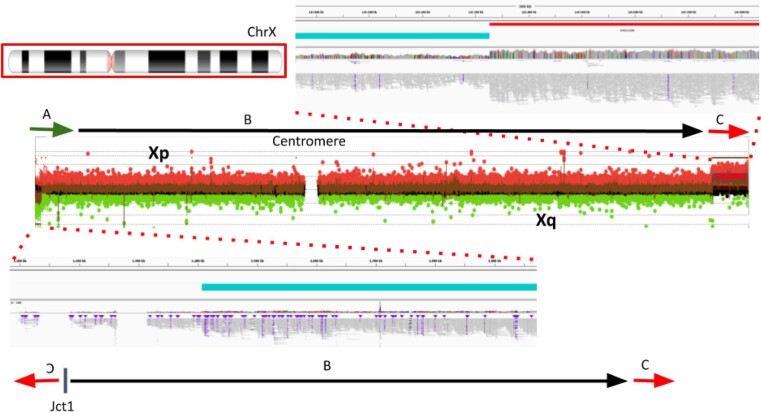
Detailed view of inversion spanning nearly the entire X chromosome called by Sniffles2. Detailed view of inversion spanning nearly the entire X chromosome (∼155 Mb) called by Sniffles2. This event is in fact not an inversion but a recombinant chromosome. This chromosomal aberration is generated *de novo* as the result of meiotic recombination in a mother carrying a heterozygous pericentric inversion. aCGH data shows a short-arm deletion (A, green arrow) and a long-arm duplication (C, red arrow). Sniffles2 is able to positionally connect the beginning of the duplication to the end of the deletion forming Jct1.

In sample BH15646_1, the inversion called by Sniffles2 spanning nearly the entire X chromosome (∼148 Mb) represents the breakpoint junction of a recombinant chromosome. In the sample, aCGH data show a short-arm deletion and a long-arm duplication—that is, a DEL-NML-DUP structure. Sniffles2 is able to positionally connect the beginning of the duplication to the end of the deletion forming Jct1 (Extended Data Fig. 2). This allele is generated as the result of meiotic recombination between heterozygous homologous X chromosomes in females harboring a pericentric inversion^57^.

Another example is represented by an apparent 311-kb inversion detected in sample BH15700_1. This inversion is part of a DUP-TRP/INV-DUP structure (Fig. 3d), which is generated by a given pair of inverted SegDups and produces an inverted triplication flanked by duplications^34^. When generated, this structure includes two breakpoint junctions (Jct) connecting the end of the duplication to the end of the triplication (Jct1) and the beginning of the triplication to the beginning of the duplication (Jct2). Although Sniffles2 can properly detect the inverted breakpoint generating Jct2, it is not able to fully resolve the context of the larger structure due to Jct1 being embedded within a pair of inverted SegDups with 99.9% sequence similarity.

In this cohort, Sniffles2 is able to correctly detect with nucleotide-level resolution the precise breakpoints defining a genomic interval in patients carrying complex genomic rearrangements (CGRs). A large portion of the CGRs in this cohort have at least one of the breakpoint junctions mapping to SegDups; those can be fully resolved by Sniffles2 together with copy number information. Additionally, Sniffles2 infers positional connections that help resolve a given complex allele architecture with information that aCGH alone cannot provide.

### Identification of mosaic SVs reveals insight in diversity

We know from many studies that germline variants are not the only source of structural variation. Often, somatic/mosaic variants are important. This has been indicated in, for example, cancer and neurological disorders^9,12^. Thus, Sniffles2 is equipped with a mosaic mode to identify low-frequency (5–20% variant allele frequency (VAF)) SVs across a single sequenced sample. Figure 1c shows the principal steps where the main innovation is to weigh the support of each read, taking into consideration its edit distance as a confidence measure. To circumvent the impact of sequencing error rates on mosaic SV detect, we filter out SVs where the average edit distance of reads supporting exceeds a threshold, which is estimated per dataset to account for different sequencing error levels (Methods).

**Fig. 4:**
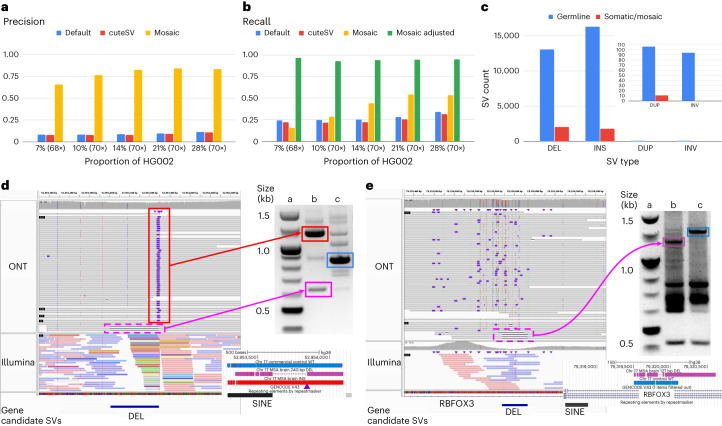
Recovery of somatic SVs using the Sniffles2 mosaic mode. **a**,**b**, Benchmark of mixtures of HG002 with HG00733. We spiked HG002 in various concentrations and measured the precision (**a**) and recall (**b**) of Sniffles2 default (blue) and mosaic (yellow) modes, alongside cuteSV (in red). For the recall, we added an adjusted recall (in green) as Sniffles2 mosaic mode calls SVs only in the range of 0.05 to 0.20 VAF, and, thus, everything outside that range will not be analyzed. **c**, Overview of the number of SV types identified as germline (blue) and mosaic (red) in the cingulate cortex brain region of an MSA patient brain sample sequenced with 55× ONT long reads. A zoom is shown for duplication and inversion SVs. **d**,**e**, Validated mosaic SVs detected by Sniffles2. Each PCR was done once (**d**)—mosaic deletion close to a germline Alu insertion. The IGV screenshot shows bulk WGS: top panel 55× ONT, bottom panel 85× Illumina. PCR validation shows both products from the MSA brain (column b, insertion in top and deletion in bottom) compared to a control (column c) and the ladder (column a). The PCR products highlighted in squares were Sanger sequenced, and the alignment is shown below the gel (colors matching), with the INS position marked with a purple triangle. **e**, Mosaic deletion within RBFOX3. The IGV screenshot shows bulk WGS: top panel 55× ONT, bottom panel 85× Illumina. PCR demonstrates the mosaic deletion (column b, wild-type in top and deletion in bottom) compared to two controls (column c, brain control) and the ladder (column a). The PCR products highlighted in squares were Sanger sequenced, and the alignment is shown below the gel (colors matching). Supplementary Fig. 6 shows the complete unannotated gels, and Supplementary Fig. 7 shows a different view of the same Illumina results for **e**. Supplementary Table 14 shows the complete list of candidate SVs, and Supplementary Fig. 8a–h shows all IGV screenshots for the same candidates.

We used a spike-in experiment of different concentrations ranging from 7% to 28% VAF of HG002 into high coverage of HG00733. Figure 4a,b shows the precision and recall of SVs across the different concentrations. Overall, Sniffles2 mosaic mode outperforms the other SV calling approaches. The recall is impacted by the fact that the subsampling occurs randomly and that heterozygous SVs are disproportionately sampled. Correcting for this fact and measuring recall on SVs occurring within 5–25% VAF improved the recall for Sniffles2 mosaic mode, as it averages 94.47% given an overall 84.12% precision. See Supplementary Section 2 and Supplementary Table 12 for details.

Next, we applied Sniffles2 mosaic mode to an affected brain region (cingulate cortex) of a patient with MSA at 55× coverage using ONT. Here, we are interested in all types of SVs, including rearrangements. In this particular case, however, we need to be alert to the possibility that chimeras can form inversions or other duplications and, as such, contribute to the overall apparent somatic SV calls. To avoid this, Sniffles2 deploys filters for low-frequency inversions that are 1 kb or smaller. Figure 4c shows the overall number of SVs and their type for both the germline and mosaic SV call sets (Supplementary Table 13). We detected a higher proportion of deletions than insertions in the mosaic calling when compared to germline (INS/DEL ratio 1.37 germline, 0.78 mosaic). We compared this ratio across all samples used in this study and found an average INS/DEL ratio of 1.10 for germline SV calling, thus clearly showing differences between mosaic and germline SVs. Supplementary Table 14 shows 34 mosaic SVs that were manually curated, and Fig. 4d,e shows two of them, both deletions, which were validated by polymerase chain reaction (PCR) and Sanger sequencing. One is overlapping a repeat element, and one affects a neuronal gene (complete gels in Supplementary Fig. 6). Figure 4d shows an example of a mosaic deletion close to a germline insertion that was identified using 55× ONT long reads (top IGV panel). We observed that these events were located between Alu elements—one novel insertion and one already pre-existing on the reference. We compared the insertion sequence to the neighboring Alu sequence and found great similarity (89.17%). This particular case is a direct orientation of an AluY, which is the Alu subfamily that is most predisposed to brain recombination and, thus, leads to mosaic deletions^58^. We then performed a blast search of the insertion sequence reported by Sniffles2 (and Sanger sequencing, 100% identity) and found that it belongs to an AluYa5. Across this sample, we could identify 25 other regions that had similar alleles of Alu insertions that lead to mosaic deletions, both identified with Sniffles. When expanding our search to other sizes of insertions, we identified a total of 206 regions where insertions might lead to an instability of the region, causing a mosaic deletion in the proximity. This again highlights the ability of Sniffles2 to recover potential interesting alleles genome wide and at scale. Figure 4d further shows discordant Illumina reads (colored), indicating multiple translocations instead of the actual Alu insertions, which we reported previously^28^.

**Fig. 5:**
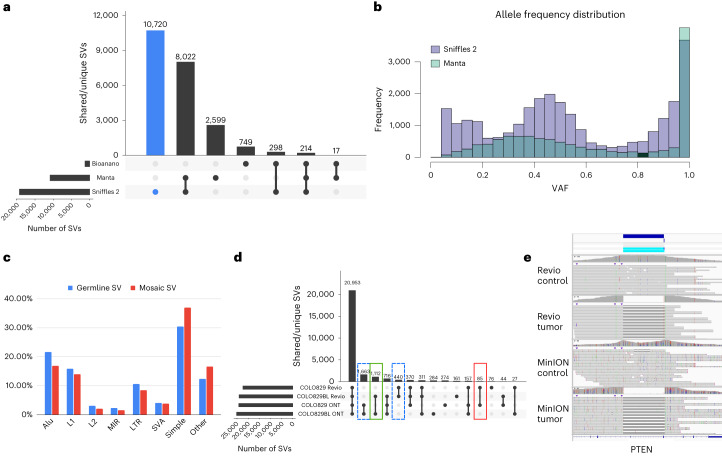
Insights into somatic SVs in the MSA patient brain sample. **a**, Overall comparison of SVs detected in ONT (Sniffles2), Illumina (Manta) and OGM datasets. **b**, Distribution of allele frequencies for SVs identified by Sniffles2 and Manta. **c**, Association of Sniffles2 germline and mosaic SVs with repeat elements. **d**, Tumor/normal comparison of the COLO829 cell line using two different sequencing technologies: ONT MinION and PacBio Revio. Highlighted are the tumor-specific SVs (in red), the normal/control-specific SVs (in green) and the technology-specific SVs (dashed lines). In the cancer-specific SV, we found variants overlapping with cancer-related genes, such as PTEN, PMS2, ARHGEF5, PAK2 and WWOX. Differences between ONT and Revio calls for the same cell line can be attributed to either technology differences or the evolution of the cell line through time. **e**, Example of a cancer-specific somatic SV that affects the PTEN gene. Both the PacBio and ONT datasets showed the same coordinates for the variant, and no read support was found in the control.

**Extended Data Fig. 3:**
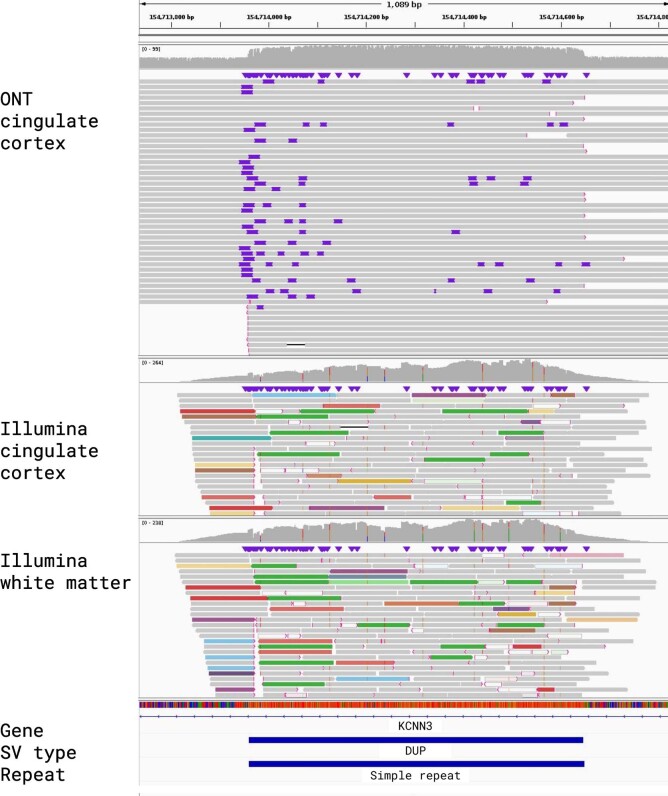
IGV alignments for a Structural Variant that was called by Sniffles2 but not represented in either the Bionano or Illumina call sets. The top shows the read alignments for the cingulate cortex ONT data, followed below by Illumina read alignments for cingulate cortex and cingulate white matter. A 687 bp mosaic duplication on chromosome 1, overlapping with simple repeats. Manual curation revealed DEL being called in the Illumina data in both cingulate cortex and white matter and no call in the Bionano data set.

**Extended Data Fig. 4:**
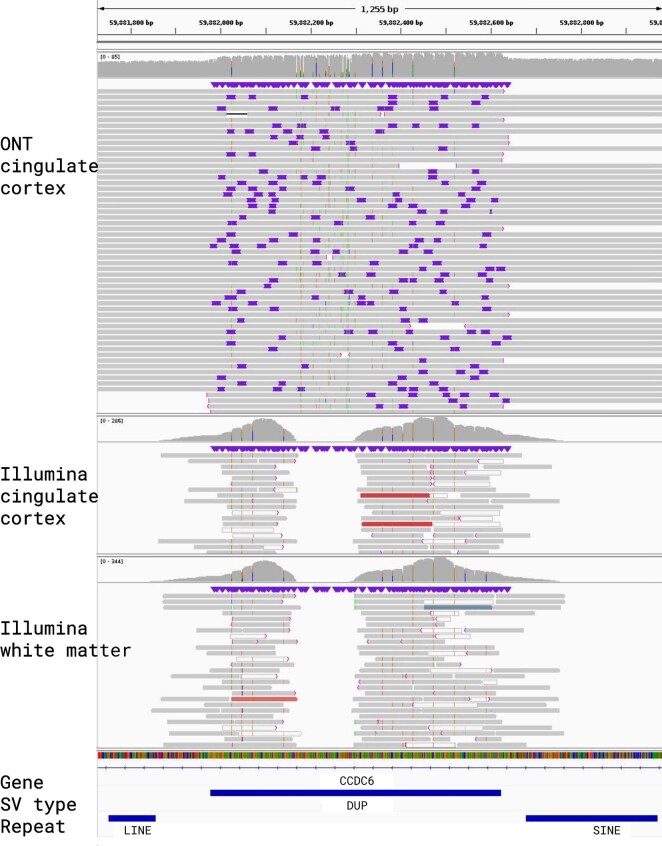
IGV alignments for a Structural Variant that was called by Sniffles2 but not represented in either the Bionano or Illumina call sets. The top shows the read alignments for the cingulate cortex ONT data, followed below by Illumina read alignments for cingulate cortex and cingulate white matter. A 645 bp non-mosaic duplication in chromosome 10 that is flanked by a SINE and LINE element. Manual curation revealed no overlap in the Illumina nor the Bionano data.

Figure 4e shows another example of a mosaic deletion, this time overlapping an intron of the *RBFOX3* gene, which encodes NeuN, a nuclear antigen used for sorting neuronal nuclei^58,59^. On manual inspection of the short reads (Supplementary Fig. 7), we observed this deletion also on the Illumina reads (five reads out of ∼85×), but it was not identifiable using Manta. Figure 4d,e also shows the result of PCR validation of both SVs. For the first validated SV (4D), the PCR gel shows both the insertion and deletion event (column b) with the proper SV length of 240 bp reported by Sniffles2. For the second validated SV (4E), the PCR gel shows evidence of the 127-bp deletion. We further validated these SVs by Sanger sequencing the PCR products highlighted in both gels, which, again, showed both deletions detected by Sniffles. Supplementary Section 3 lists details on the impact of SV on genes and overlap of repeats for this MSA sample (Fig. 5c, Extended Data Figs. 3 and 4, Supplementary Figs. 8 and 9 and Supplementary Tables 15 and 16).

Next, we compared the different technologies to the Sniffles2 results. The same brain region was also sequenced by Illumina short reads (90×) and analyzed by Bionano OG (690×) (Methods). The variant calls from Sniffles2 for both germline (21,965) and mosaic (2,937) were concatenated as the VAFs between them are mutually exclusive. For Illumina, Manta^60^ detected 12,142 SVs, and OGM (5 kb or larger) detected 1,463 SVs. Figure 5a highlights the agreement of all SVs detected in the same sample by the three technologies for a minimum length of 50 bp and excluding translocations (Supplementary Table 17.1). Supplementary Section 4 provides details on the comparison. Overall, we saw disagreement between OGM and Illumina mainly driven by size or SV type differences (for example, insertions or rearrangements). We further noted a shift in the variant allele frequencies (VAFs) across the Manta calls compared to the Sniffles2 calls (Fig. 5b), which further impacts the overlap between the technologies.

Finally, we focused on the non-germline (mosaic/somatic) SVs exclusive to the cingulate cortex brain region. For this, we also sequenced the neighboring cingulate white matter from the same patient using Illumina. We used SVTyper^61^ to genotype Sniffles2 SVs (only deletions, duplications and inversions) that were not initially identified by Manta against the aligned Illumina reads from both brain regions (Supplementary Table 17.3). This way, we identified 497 SVs that initially were not identified in Illumina but were genotyped as present. We identified 484 non-germline SVs using Sniffles2 that have Illumina read support in the neighboring brain region, thus showing that Sniffles2 is able to accurately detect low-frequency (that is, mosaic) SVs.

Given these advancements in mosaic and germline calling, we further tested Sniffles2 across COLO829/COLO829BL. Figure 5d shows the overlap across ONT and PacBio calling using Sniffles. Details on the results are in Supplementary Section 5. Supplementary Fig. 10 and Supplementary Table 18 show the benchmark results; Fig. 5e and Supplementary Fig. 11 show examples of cancer-specific germline SVs; and Supplementary Fig. 12 show examples from the benchmark.

## Discussion

Here we present an updated version of the highly popular SV caller Sniffles (Fig. 1). Sniffles2 is a substantial improvement in terms of accuracy and runtime compared to all other commonly used long-read-based SV callers (Fig. 2). We show higher accuracy across different coverages (5–50×) using different sequencing technologies (PacBio HiFi and ONT) and even across all SV types. This is achieved by an automatic parameter optimization that is part of Sniffles2 compared to all other SV callers that require manual adjustments. Besides this, Sniffles2 is also able to genotype SV and leverage phased reads (using haplotype phase (HP) and phase state (PS) tags) as input to provide phased SV in a VCF file. We demonstrated a genomic VCF (gVCF) concept for SV calling and implemented a working version in Sniffles2. This instantaneously halves the requirements of computing and storage for population/family SV or even tumor versus normal SV calling (Supplementary Section 5), thus resolving the ever-larger demands of long-read datasets^21^. Furthermore, it solves the n + 1 problem when a new sample is added on a later stage of the project. We demonstrate the utility across the 31 ONT Mendelian samples, where Sniffles2 resolved SVs mapping to complex regions of the genome with a direct impact to disease, supporting copy number data. This clearly illustrates the benefit of this approach that can easily scale to new population long-read challenges. For cancer applications, there are in development other somatic SV callers^62^ that are specialized on tumor versus normal tissue comparison. In contrast to them, Sniffles2 is a general purpose SV caller that can also be used to detect cancer-specific somatic but further mosaic SVs. Furthemore, the same strategy can also be used to compare different tissues within the same organism.

We demonstrated this approach using a synthetic dataset of HG002 and a genetically unrelated individual HG00733. This showed the accuracy and recall of Sniffles2 while depending on only 2–3 reads overall to distinguish SV from noise (Fig. 4a,b). We then turned our attention to MSA, a rare sporadic neurodegenerative disease related to Parkinson’s disease, with negligible heritability (<7%)^63^. We performed ONT WGS on an affected brain region from one patient, where Sniffles2 was able to identify presumptively low-frequency mosaic SVs and showcased great performance partially validated by Illumina and OGM approaches, thus overall highlighting the fact that Sniffles2 is highly versatile and accurate. Although thresholding on the VAFs (here, 5–20% AF) for the identification of potential somatic variants is straightforward, there is still a gray area to be addressed. For multiple SVs, we saw a continuum in VAF (between 20% and 30% AF), which suggests that some SV with apparent AF < 30% may also be germline. Thus, the comparison to population data or to different tissues is still favorable (for example, tumor versus normal). Furthermore, it is interesting to note that the insertion versus deletion characteristics change between germline and mosaic. We attribute this to the many cases of repeat mediated recombination that we could identify that manifest as deletions. It is also interesting to note that the detection of tissue-specific SVs as proposed here can be impacted by multiple biases. First, we can have a detection bias in the Illumina data (for example, insertions), but, furthermore, a sampling bias in the other tissue might also result in tissue-specific SV detection. The possible role of somatic SVs in MSA is under investigation^43^, although further validation data from more cases and controls would be required to allow interpretation of the present findings. In our experiments at mosaic level, we identified many more deletions than insertions in contrast to germline (AF > 0.2). We speculate that this is indeed a biological signal and not a detection bias due to non-allelic homologous recombination (NAHR) or other mediated mechanisms.

Despite solving central problems of SV calling at scale and accuracy for long reads, many challenges remain. High-quality benchmarks for complex SVs are lacking. Sniffles2 does not yet solve the issues with highly rearranged regions where SVs can be overlapping with each other. This remains a near-future goal of Sniffles2 and will also require improved benchmark sets and even standards to report these events, as the VCF standard does not provide a clear recommendation. Currently, these complex alleles would need to be reported as independent breakend (BND) events, which lose their individual impact (for example, DUP-TRP/INV-DUP and DUP-NML-INV/DUP) on the region itself. Nevertheless, this is clearly needed, as our experiments on the Mendelian cohort show.

Overall, we report here the innovations across Sniffles2 and highlight them across Mendelian cases, a patient with MSA and a tumor/normal comparison. We think that our implementations will spark discoveries across human diseases and diversity. Furthermore, we think that these will also be important for other species. Despite the fact that the genotype model for Sniffles2 is designed for diploid organisms, Sniffles2 is capable of also detecting SVs in haploid (as shown for X chromosomes in males) or polyploid organisms. For higher ploidy levels, we would suggest running the mosaic mode as, otherwise, the genotype caller will penalize true SVs, thus again highlighting Sniffles2 as a highly accurate and versatile method to detect SVs of any kind and property.

## Methods

### Patient enrollment

The 31 individuals (proband and parents) included in this study were enrolled into research protocols approved by the institutional review board (IRB) at Baylor College of Medicine and the Pacific Northwest Research Institute (H-29697 and H-47127 and WIRB 20202158).

### Sniffles2 methodology

#### Sniffles2: germline calling

An overview of the steps involved in the Sniffles2 germline SV detection algorithm is shown in Supplementary Fig. 13.

Sniffles2 germline mode accepts aligned long reads as input (BAM or CRAM format, sorted by genomic coordinate and indexed). First, read alignments are parsed and pre-filtered based on minimum mapping quality (default, 20), minimum alignment length (default, 1 kb) and maximum number of split alignments (default, 3 + 0.1 *ReadLengthKb*). Split alignments are analyzed to extract SV signals for insertions, deletions, duplications, inversions and BNDs. Next, to analyze splits, inline alignments are scanned for insertion and deletion signals. Sniffles2 does not merge nearby inline insertion and deletion events at this point. SV signals that fulfill a minimum length threshold (default, 0.9 *MinSVLength*) are subsequently recorded in high-resolution genomic bins. Start and end positions of alignments are recorded in a separate data structure for facilitating later coverage computation without requiring reopening of alignment files.

Sniffles2 employs a three-phase clustering process to translate individual SV signals into putative SV candidates. First, SV signals extracted from reads in the pre-processing step are clustered based on their indicated SV type and genomic start position. Second, insertion and deletion sequences in each cluster stemming from the same read are merged to correct for alignment errors in highly repetitive regions. Third, preliminary clusters are re-split to represent different supported SV lengths.

The first clustering phase constitutes a fast pass over all bins (default bin size, 100 bp) containing SV signals extracted from alignments in the pre-processing step. Bins are traversed from chromosome start to end separately for each of the five basic SV types. Neighboring bins are merged if the inner distance between them is smaller than a threshold calculated based on the minimum standard deviation of the genomic SV start positions within each bin. The inner distance threshold *d*_*n*_ is calculated as ${d}_{n}=r\cdot \min ({\sigma }_{{StartA}},{\sigma }_{{StartB}})$, where *r* is a constant (default, 2.5), and ${\sigma }_{{StartA}}$ and ${\sigma }_{{StartB}}$ refer to the standard deviation of indicated SV start positions in the two neighboring bins, respectively. In regions spanning tandem repeats, a more relaxed clustering criterion is applied. Neighboring bins are also clustered when their outer distance falls below a threshold defined based on the indicated average SV length of the SV signals stored in the neighboring bins. This threshold *d*_*r*_ is calculated as ${d}_{r}=\min ({h}_{{max}},{h}\cdot [{x}_{A}+{x}_{B}])$, where *h* and *h*_*max*_ are constants (default, 1.5 kb and 1 kb, respectively) and *x*_*A*_ *and x*_*B*_ refer to the mean indicated SV length in the two neighboring bins. Whenever two neighboring bins have been merged, the clustering is restarted at the bin preceding the merged pair, facilitating the growth of SV clusters in both upstream and downstream directions. The first clustering phase is completed as soon as the last bin in the chromosome has been reached.

The second clustering phase constitutes merging of insertion and deletion events stemming from the same read that have been placed within the same initial cluster. Events with an inner distance closer than the set threshold (default, 150 bp) are merged. In areas of tandem repeats, the distance threshold is set to the size of the initial cluster itself.

In the third phase, clusters are split by indicated SV length of the contained SV signals and subsequently re-merged, which leads to the final separation of SVs that share a start position on the reference but have different lengths. Bins are traversed from those containing small to large SV signals and merged in a similar fashion to phase one, based on the relative difference in SV length between neighboring bins being no larger than a given threshold (default, 0.33). In clusters overlapping tandem repeats, Sniffles2 does not perform resplitting.

Differentiated clustering parameters are applied to BND-type SVs, because no length is available as a metric to drive clustering.

At the beginning of post-processing, SV candidates are generated from the final clusters resulting at the end of the last stage. Start coordinates and SV length are determined based on the median of the most common values supported by the reads. Standard deviations are calculated for the trimmed distribution of indicated SV start position and lengths. The quality value is summarized as the mean mapping quality of supporting reads. SVs are labeled as precise when the sum of SV start and length standard deviation is less than the set threshold (25 bp).

SV candidates are filtered based on absolute and relative (compared to the SV length) standard deviation of their coordinates. In addition, type-specific coverage filtering is applied to deletions and duplications, requiring central coverage changes consistent with the detected variant. Instead of requiring users to settle for a predefined, static minimum read support threshold, Sniffles2 dynamically adjusts the minimum support value based on estimates of global and regional sequencing coverage. By default, the minimum read support threshold is calculated as ${MinSupport}=\alpha \cdot ([1-\lambda ]{C}{global}+\lambda {C}{local}$, where *Cglobal* and *Clocal* refer to average chromosomal and SV surrounding coverage, respectively. The parameters are set as *α* = 0.1 and *λ* = 0.75, by default. For insertion and deletion SVs, support from inline alignments and split alignments is output separately. Additionally indicated support from soft-clipped reads is additionally recorded for insertion SVs.

Genotypes are determined using a maximum-likelihood approach. The genotype quality is calculated based on the likelihood ratio of the second most likely to the output genotype: $Q=-10{lo}{g}_{10}({L}_{2}/{L}_{1})$, where *L*_*1*_ and *L*_*2*_ refer to the likelihood of the most likely genotype and the second most likely genotype, respectively. Genotype likelihoods are computed for a binomial distribution for the observed number of variant and reference reads. Genotype likelihoods are set as 1.0-ß for 1/1, 0.5 for 0/1 and ß for 0/0, where ß represents the genotype error introduced through sequencing and alignment artifacts and is set to ß = 0.05 by default.

For insertion SVs, sequencing and read aligner errors are corrected using a fast k-mer-based pseudo-alignment method. Through this, Sniffles2 generates a consensus sequence in two steps. In the first step, the best possible starting sequence is chosen from the supporting read with the smallest distance in SV start position and length to the final reported SV coordinates. K-mers (default length, 6 bp) are enumerated for this read-supported insertion sequence, and a taboo set of repetitive k-mers, which occurs more than once in the sequence, is built. Simultaneously, the positions of non-repetitive k-mers are stored in an anchor table to facilitate pseudo-alignment of the other reads. In the second phase, k-mers from other read insertion sequences are enumerated. When a k-mer is present in the anchor table, the corresponding position in both the initial insertion sequence and the current read is stored. After all reads have had their k-mers anchored, sequences between anchored k-mers are extracted from the pseudo-aligned reads. These sequences from between the anchored k-mers constitute the parts of each read insertion sequence in disagreement with the initial sequence. Finally, coordinates of the initial sequence are traversed, and the consensus is generated as the most common base at the respective position throughout all pseudo-aligned reads. Long insertions (that is, multiple kbp) are often difficult to detect even in long-read data because reads often do not span the full insertion sequence. To improve detection of long insertions, Sniffles2 records these clipped read events as additional support for presence of a large insertion. This enables Sniffles2 to accurately detect large insertions even when the SV is fully covered by just a single read.

Post-processed and annotated SV calls that passed quality control checks are written to the output VCF file. Quality control filters applied to SV candidates by default include absolute and relative standard deviation of the SV breakpoints, coverage change for copy number variants and minimum coverage in the surrounding genomic region. Additionally, all unfiltered SV candidates and genome-wide coverage information are written to a specified output SNF file, which may be consecutively used as input for multi-sample calling (See below, combined calling). Using the --qc-output-all option, all unfiltered candidates (except for the minimum SV length filter) can also be directly written to the VCF output file complete with the respective reasons for why they would have been filtered by default.

Full parallelization across chromosomes is applied through all key steps in Sniffles2, including pre-processing, clustering and post-processing. The final SV calls are written to a sorted VCF output file. Alternatively, Sniffles2 also supports direct output to a sorted, bgzipped and tabix-indexed VCF file.

#### Sniffles2: combined calling (population mode)

Sniffles2 produces a fully genotyped population VCF file by introducing a specialized mode (‘Sniffles2 combine’) for both family-level and population-level SV calling. ‘Sniffles2 combine’ is built around an SNF file, designed to store a complete snapshot of structural variation and sequencing coverage for a single sample. Mergeable SNF files for later population-level calling are designed to be easily produced as a side-product of regular single-sample SV calling using Sniffles2, by using the optional --snf output argument. Based on individual use case requirements, Sniffles2 can simultaneously produce SNF files and/or regular VCF files in a single run of processing an individual sample.

SNF files consist of a JSON-based index followed by a series of multiple gzip-compressed blocks (separated by genomic coordinates). Each block stores all putative SV candidates, separated by SV type, for a single sample’s respective genomic region. This includes candidates only supported by, for example, a single read that would normally be ignored. Each block furthermore stores sequencing coverage information (500-bp resolution by default). All stored SV candidates contain a compressed form of all the information of the final SV calls, as they would be output in a single-sample VCF file, such as start, end positions, standard deviation and alternative alleles. SNF blocks span a genomic region of 100 kb by default. This small block size comparison to a typical mammal genome allows Sniffles2 to combine a high number of samples simultaneously while keeping a manageable memory footprint.

SNF files, once generated, can then be used as input for the ‘Sniffles2 combine’ mode, producing a final, fully genotyped population-level VCF file within seconds. SNF files may also be reused in the combine step—for example, when the population is later on extended, when individual samples need to be re-run or when querying whether a later newly identified SV is present in a population. These use cases would not be possible without costly re-processing of all samples with the currently prevalent method of forced calling. A schematic of SNF file structure can be found in Supplementary Fig. 14.

When presented with multiple SNF files as input, Sniffles2 combines them through a single pass over chromosomal regions. For each region, the respective SNF blocks overlapping it are loaded, including all SV candidates and coverage information from each sample. In the following step, Sniffles2 groups the loaded SV candidates based on SV type and coordinate-based matching criteria. For each SV candidate, Sniffles2 first checks if there is an already existing, matching group. An SV candidate matches a group if it has the same SV type and the sum of start position and length deviation is less than $M\cdot \sqrt{\min ({SVLength},{GroupSVLength})}$, where *M* is set to 500 bp by default (user-adjustable). The start position and SV length of a group are defined as the arithmetic mean of all SVs currently contained in it. In case there are one or more groups that fulfill the matching criteria for the current SV candidate, the group with the smallest deviation metric is chosen, and the SV candidate is placed therein. The coordinates of the selected group are then subsequently updated to represent the new average position of length of the contained candidates. If there are no matches, a new SV group is created. By default, Sniffles2 allows for matching multiple SVs from the same sample within a group (can be disabled using a dedicated parameter).

This partition of SNF files into individually loadable blocks keeps Sniffles2 memory footprint manageable even when processing a high number of samples and/or samples with high coverage. Sniffles2 further implements a dynamic binning strategy for accelerating the grouping phase. Sniffles2 first assigns all loaded SV candidates from the current chromosomal region to bins based on SV type and start position. Bins are then traversed from low to high coordinate within the current block while collecting encountered SV candidates. When the number of SV candidates exceeds a certain threshold (default, *PopulationSize* × 0.5), the collected SV candidates are grouped as described above. Triggering the grouping stage only when a set number of SV candidates is reached allows for the highest possible accuracy in matching SVs from different samples in regions with low complexity while keeping the runtime manageable even in regions with a high density of SV candidates. To avoid edge effects, the final resolving of SV groups with genomic coordinates close to the ends (default, <2.5 kb) of the respective bin are carried over and finally resolved in conjunction with the grouping of the next bins. The same strategy is applied to SV groups close to the genomic start or end coordinate of the currently processed SNF block.

By default, the ‘Sniffles2 combine’ mode will output all resulting SV groups in the population that meet at least one of two criteria:

A. The SV has been detected with high confidence (that is, passes all quality control checks) in at least one sample and/or
B. By default, to have a high-confidence call in at least one sample.

The SV is present in a sufficiently high number of individual samples, even though it may not have passed individual quality control checks (default, present in at least $\max (0.2{PopulationSize},2)$ samples). These parameters are also user-adjustable and can be adjusted or disabled without having to re-generate the SNF files for the individual samples.

Each final SV group that passes the above criteria is output as an SV in the final population-level VCF file, including the genotypes from all samples. For samples that did not have an SV candidate that could be matched to the group, Sniffles2 first uses the coverage information stored in the SNF file of the respective sample to determine if the sequencing depth at around the group’s genomic location was sufficiently high (default value, 5×). If it is, the sample genotype for that SV is output as 0/0 if there is no evidence and, otherwise, as missing (./.). For all SVs, the number of reads supporting the SV and supporting the reference are output for all samples, allowing for differentiation between true biological and technically induced absence of each SV from a sample.

‘Sniffles2 combine’ is fully parallelized, allowing leveraging multi-core CPU systems not just for calling individual samples but also for the final combination step. This, in conjunction with the separation of SNF files into blocks and dynamic binning strategy, together enables Sniffles2 to perform scalable population-level SV calling.

#### Sniffles2: low-frequency SV (mosaic) calling

In the mosaic mode, a reduced default minimum support multiplier is applied (default, 0.025) to increase sensitivity for low-frequency SVs. At coverage levels of 30× to 50×, this leads to a minimum read support of 2–4 reads for the detection of mosaic SVs. To balance out the increased influence of sequencing and alignment artifacts at this lowered read support threshold, additional filtering based on alignment quality is applied. In the pre-processing steps, the length-weighted number of mismatches is recorded for all SV signals, excluding insertions and deletions. After calling, SVs with an average weighted mismatch ratio of larger than a threshold *t* = *c* × *a*, where *a* is the average length-weighted mismatch number for all reads and *c* is a constant (default, 1.66), are filtered. The additional, coverage-based filtering steps for CNVs applied in the germline mode are not applied in mosaic mode, as coverage changes induced by somatic SVs are not reliably measurable.

### Benchmarking methodology

#### Computer specifications

All tests were performed in a high-performance cluster with Intel Xeon Gold 6148 CPU @ 2.40 GHz; the memory allocation was 32 Gb unless otherwise stated; and the number of CPU cores allocated was eight unless otherwise stated. All CPU time is given as the sum of all compute times as if a single core was used.

#### Benchmarking SV callers on GIAB, 1000 Genomes and Challenging Medical Relevant Genes

Reads were mapped using minimap2 (ref. ^64^) (version 2.17-r941) technology-specific preset parameters. Reference genome GRCh37 was used to test for the GIAB version 0.6 SV benchmark, and GRCh38 was used to test the Challenging Medical Relevant Genes (CMRG) SV panel. In both cases, the ALT and/or Decoy contigs were not included. The -Y option was supplied to disable hard clipping (required by pbsv) and generate the --MD tag (required by Sniffles1), and the PacBio/ONT presets were used, respectively. Resulting alignments were converted to BAM format, sorted and indexed using SAMtools (version 1.13).

As measure of coverage across all benchmarked datasets, we used the mapping coverage as reported by mosdepth^65^ (version 0.3.2), which was averaged across all autosomes.

In addition to GIAB’s HG002 sample, we also benchmarked SV on three assemblies from 1000 Genomes (HG01243, HG02055 and HG02080). Here, we leveraged the phased HiFi assemblies provided at https://github.com/human-pangenomics/hpgp-data and the corresponding long reads. The benchmark set was derived from a dipcall^66^ (version 0.2) alignment against the GRCh38 reference. This result was used together with the corresponding BED files for benchmarking^67^.

We used Truvari^50^ (version 2.1) for benchmarking the accuracy of all SV callers across datasets. For benchmarking, we used the --*passonly* parameter to include only those SVs from caller and gold standard that are not marked as filtered. For the GIAB benchmarks, we additionally used the --*giabreport* parameter to generate the benchmark-specific detailed report. As included regions, Tier 1 regions were used unless otherwise specified. For all other parameters, default values were used.

Callers were first benchmarked using default parameters, and callers other than Sniffles2 were separately benchmarked on GIAB by manually setting the minimum read support parameter to 2 (sensitive).

SVIM^49^ (version 1.4.2) does not include filtering steps in its main pipeline, which caused it to perform poorly (F-measure) in most benchmarks, and we were not able to identify a recommended default cutoff for the quality value that SVIM outputs along with its SV calls. Therefore, in line with previous SV caller benchmarks, we filtered the output of SVIM to include only calls with a minimum read support of 10 by default (equal to the default of cuteSV and Sniffles1) or 2 (sensitive).

For benchmarking Sniffles2 (build 2.2), we only used the default parameters with the exception of mosaic SVs, where the --*mosaic* option was supplied. For Sniffles^28^ (version 1.12), default parameters were used. For cuteSV^47^ (version 1.0.11), we used the additional parameters recommended by the authors for use with HiFi/ONT datasets in their GitHub documentation as well as the --genotype option. For pbsv^48^ (version 2.6.2), we supplied the --ccs option for analyzing HiFi data, as recommended by the authors. Both pbsv and Sniffles2 support the use of tandem repeat annotations for improving SV calling in repetitive regions. For pbsv and Sniffles2, we, therefore, supplied the tandem repeat annotations for GRCh37/GRCh38, which we obtained from the pbsv repository on GitHub: https://github.com/PacificBiosciences/pbsv.

For all SV callers that have an option for specifying the number of multi-processing threads, we set the number of threads as 8. We measured and reported the total CPU time and wall clock time using the UNIX time command. For the benchmarks including only insertions and deletions, we used SnpSift^68^ (version 4.3t) to filter the output of all SV callers to include only those types of SVs. To prepare SV caller output for benchmarking, VCF files were sorted using BEDTools, compressed and indexed using bgzip and tabix. For SVIM, SVs labeled as INS:NOVEL were re-labeled to INS, to be able to be matched to insertions in the benchmark sets by Truvari. Genotype F1 measure for the detection of insertions and deletions by genotype and SV length are shown in Supplementary Fig. 15.

#### Simulation of different SV types using SURVIVOR

SURVIVOR^56^ (version 1.0.7) was used to simulate SV types not covered by the GIAB and other benchmarks. For this benchmark, 3,000 duplications, inversions and translocations were each simulated within a length range of 500 bp to 30 kb on the human reference genome GRCh37 in diploid mode. A total sequencing depth of 30× was simulated for ONT reads, with the error profile obtained using the SURVIVOR scanreads command from the HG002 ONT Q20+ dataset. SVs were called using each SV caller for the simulated reads using the default parameters and post-processing steps also used in the GIAB and other benchmarks (see respective Methods subsections). The SURVIVOR eval command was used (matching threshold, 500 bp) to obtain true-positive (TP), false-negative (FN) and false-positive (FP) counts for each caller and simulated SV type from which precision, recall and F-measure were calculated.

#### Measurement of insertion sequence accuracy

Accuracy of insertion sequences recovered by the SV callers was measured using Biopython’s^69^ (version 1.79) pairwise2 global alignment function. First, the TP calls from all investigated SV callers on the dataset were intersected, to establish a common set of calls to benchmark. Next, the gold standard and reported insertion nucleotide sequences were aligned, and the resulting score was normalized by length of the gold standard sequence to compute the alignment identity. We measured sequence accuracy separately for the GIAB HiFi and ONT datasets (30× coverage). Results are shown in Supplementary Fig. 1. The respective script is available in the Supplementary Materials.

### Simulation of low-frequency SVs

Low-frequency SVs were simulated by combining varying coverage titrations of HG002 and HG00733 into synthetic samples with different levels of mosaicism. Recovery of SVs unique to HG002 was done based on the intersection of SVs of the same type using BEDTools with 50% coverage of the SV reciprocally against the benchmark set of HG0733 (ref. ^50^). These unique SVs were then used to benchmark to measure recall for low-frequency SVs. For benchmarking the ability of Sniffles2 to detect low-frequency SVs, we simulated synthetic datasets with 63×/5×, 63×/7×, 60×/10×, 55×/15× and 50×/20×, where the coverage refers to HG00733 and the second one to HG002. Next, we used the previously selected HG002 unique SVs overlapping the GIAB Tier 1 benchmark. To measure recall for low-frequency SVs, we ran Sniffles2 in mosaic mode on the synthetic samples and used Truvari as described in the Methods subsection on GIAB benchmarks to compute the recall for the rare HG002 SVs introduced into each HG00733 dataset. Simultaneously, we ran Sniffles2 and cuteSV with default parameters and benchmarked the results for comparison. Given that Sniffles2 mosaic mode analyzes and reports SV only within a defined VAF (5–20%), we excluded all SVs that were outside of such VAF to compute an ‘adjusted recall’. As in all the other GIAB benchmarks, analysis was limited to insertion and deletion SVs. Distribution of SVs from HG002 by their AF is shown in Supplementary Fig. 16.

### MSA patient analysis

#### Optical mapping data on MSA patient brain

Ultra-high molecular weight (UHMW) DNA was isolated from frozen human brain tissues using a Bionano Prep SP Tissue and Tumor DNA Isolation kit (no. 80038) according to the Bionano Prep SP Brain Tissue Isolation Tech Note (no. 3400). In short, approximately 20 mg of frozen tissue was homogenized using a Qiagen TissueRuptor (no. 9002755), passed through a 40-µm filter and treated sequentially with Qiagen protease (cat. no. 19155), proteinase K and RNAse A in lysis and binding buffer. The homogenate was then treated with PMSF to de-activate the protease and proteinase K, washed and eluted. The extracted DNA was mixed using an end-over-end rotator for 1 h at 5 r.p.m. and allowed to rest at room temperature until homogenous (approximately 1 week). Then, 750 ng of purified UHMW DNA was fluorescently labeled at the recognition site CTTAAG with the enzyme DLE-1 and subsequently counterstained using a Bionano Prep DLS Labeling Kit (no. 80005) following the manufacturer’s instructions (Bionano Prep Direct Label and Stain (DLS) protocol no. 30206). OGM was performed using a Saphyr Gen2 platform for a final effective coverage of 894× for the pons and 754× for the cingulate. Effective coverage is defined as the total raw coverage of molecules ≥150 kbp in length multiplied by the proportion of molecules that aligns to the reference genome.

Calling of low AF SVs was performed using the rare variant analysis pipeline (Bionano Solve version 3.6) on molecules ≥150 kbp in length. De novo assembly was performed using the longest 250× molecules of each dataset. The variant annotation pipeline (Solve 3.7) was used to detect which SV calls in the cingulate are present in the pons SV calls and/or molecules. See the Bionano Solve Theory of Operations for more details.

#### MSA sample comparison

Illumina reads were mapped to the human genome GRCh38 using bwa^70^ mem (version 0.7.17-r1188) with default parameters, including -M to mark split reads as secondary alignments. Subsequently, we identified SV using Manta^60^ (version 1.6.0).

For ONT, reads were mapped using minimap2 (ref. ^64^) (version 2.17-r941) with present parameters for ONT. Subsequently we identified SV using Sniffles2 with both germline (default) and mosaic mode. The Bionano OGM data smap file was converted by SURVIVOR smaptovcf (version 1.0.7) into a VCF file.

To compare SVs called by Sniffles2, Manta (Illumina) and OGM (Bionano), we used SURVIVOR merge using a 10-kb threshold, matching SV type and ignoring reported SV strand. We extended it to 10 kbp after testing 500 1-kbp and 5-kbp thresholds and observed that the accuracy of the breakpoints from OGM required the larger parameter.

The genotype columns in the SURVIVOR merge output were compared for each SV to determine presence or absence in the results reported by the respective method.

Subsequently, to further investigate SVs absent from the Manta call sets, we additionally genotyped the respective Sniffles2 calls against the raw Illumina read alignments for the same brain region (cingulate cortex) as well as a different brain region (cingulate white matter) using svtyper (version 0.7.1)^61^. SVs reported as having at least one supporting read by svtyper were considered as present in a sample.

#### PCR validation of selected mosaic deletions

We used the National Center for Biotechnology Information (NCBI) primer design tool to obtain primers straddling the target deletions. The primer sequences for the 240-bp deletion were TACCAAGTCTTTCTCCAAGTCCC (forward) and TTGCACAGCCTTGGCTATACTC (reverse) and, for the 127-bp deletion, ATCCTGAGAGAACCCCCTCC (forward) and GGACAGACTCGTGGTTTCGT (reverse). PCR was performed using Phusion Plus PCR Master Mix (Thermo Fisher Scientific), with 0.5 µM primers, annealing temperature 60 °C and extension time 75 s. PCR results were confirmed using Agilent TapeStation and 2% agarose gel electrophoresis, stained with GelRed (Biotium), with 100-bp DNA ladder (New England Biolabs). Initial PCR was performed using 20–40 ng of template DNA in 20 μl for 35 cycles. Repeats to obtain adequate products were performed using 100 ng of DNA in 50 μl, with 40 cycles for the second deletion, and low-melting-point agarose was used to allow relevant amplicon band excision. Extraction and purification from agarose was carried out using a QIAquick Gel Extraction Kit (Qiagen). Extracted products, which represented the wild-type, deletion and Alu insertion, underwent Sanger sequencing

### Mendelian inconsistency benchmark in population mode

#### Mendelian benchmark/inconsistency

To assess the performance of Sniffles2 population mode, we used the Ashkenazim family trio. We called SV using Sniffles2 and cuteSV. For Sniffles2, we used a minimum SV length of 50 and with the output being the SNF binary file that contains the unfiltered SV candidates and genome-wide coverage information (using the --snf option). Then, we merged the SNF files with Sniffles2 population-level calling providing the reference genome to obtain the sequences of the deletions. Here, the input is the SNF files and the output the VCF file. For the case of cuteSV, we used version 1.0.11 with recommended parameters for Oxford Nanopore data (--max_cluster_bias_INS 100 --diff_ratio_merging_INS 0.3 --max_cluster_bias_DEL 100 --diff_ratio_merging_DEL 0.3). Then, we merged the results of cuteSV using SURVIVOR version 1.0.7 with a maximum distance between breakpoints of 1 kb, a minimum support of 1 and taking into account the SV type. Next, we performed force calling with cuteSV, using as input the merged SV from SURVIVOR (-Ivcf and --genotype options). Finally, we performed a second merge with SURVIVOR with identical parameters as before.

We then tested the Mendelian inconsistency of the genotypes using the BCFtools version 1.14 Mendelian plugin^55^. The Mendelian plugin denotes a genotype consistent when the proband genotype is in concordance with the parental genotypes (for example, F 0/0, M 0/1 and P 0/0), inconsistent when the proband and parental genotypes do not match (for example, F 0/1, M 1/1 and P 0/0) and NA when the proband has a missing genotype (./.). For all analyses, time was measured using the linux time command.

### Chromosome X disorder patient analysis

Sniffles2 population mode was used to analyze 31 ONT samples that represented cases of Mendelian disorders in probands. We obtained the BAM files by running PRINCESS^29^ (version 1.0) using the default parameters and ‘ont’ flag. PRINCESS implicitly calls Minimap2 (ref. ^64^) (version 2.17) with the following parameters ‘-ax map-ont -Y --MD’. Later, we sorted the output using SAMtools^55^ (version 1.14). For all samples, unfiltered SV candidates and genome-wide coverage information are written to a specified output SNF file and then merged with Sniffles2 population-level calling. General statistics, such as SV sizes and composition (proportion of each SV type), were computed by extracting the SVLEN, SVTYPE and GT information from the VCF file.

Given the nature of the dataset, only the SV calls from chromosome X were analyzed. Additionally, for specific individuals (BH14379 and BH14413), SVs from chromosome Y were analyzed given that both aCGH and Sniffles2 called translocations to chromosome Y. Then, all SVs that were less than 10 kb were filtered, as aCGH data showed that large events were involved. Finally, we filtered out SVs that occurred in the father, as this disorder is fully penetrant in males by comparing the SUPP_VEC tag in the VCF to the sample names. Manual curation was performed for a single SV that was filtered out by the STDEV_LEN filter of Sniffles2 during development.

### Identification of cancer-specific somatic SVs by Sniffles2

We used the population-level calling (population merge) of Sniffles2 to detect cancer-specific somatic SVs by comparing a tumor/normal pair. We used the highly studied COLO829 cancer cell line with the COLO829BL blood control. SVs were called with Sniffles2 using default parameters with the --snf option to save candidate SVs to the SNF binary file, per sample. We used two tumor/normal pairs, one described in Vale-Inclan et al.^71^ and a sample provided by PacBio (see ‘Data availability’). We then merged the four files using Sniffles2 population merge. Next, we analyzed the SV presence/absence by means of the SUPP_VEC tag in the INFO field of the output VCF to extract SVs that are detected only in the tumor samples. We compared all the SVs detected by Sniffles2 to the COLO829 SV benchmark set to assess the performance of Sniffles2 somatic SV calling. For the case of mosaic SVs, we performed the same strategy as before; moreover, for the cancer datasets, we added the --*mosaic* option to get the mosaic candidate SVs in the SNF file as well. Here, we also detected somatic SVs but the presence/absence by means of the SUPP_VEC tag in the INFO field to extract cancer-specific SVs.

### Reporting summary

Further information on research design is available in the Nature Portfolio Reporting Summary linked to this article.

## Online content

Any methods, additional references, Nature Portfolio reporting summaries, source data, extended data, supplementary information, acknowledgements, peer review information; details of author contributions and competing interests; and statements of data and code availability are available at 10.1038/s41587-023-02024-y.

## Supplementary information

Supplementary Information Supplementary Sections 1–5 and Supplementary Figs. 1–16

Reporting Summary

Supplementary Tables 1. Performance metrics for correctly identifying and genotyping SVs across ONT and PacBio HiFi. Additionally, we included two examples of CLR data. 2. Performance with respect to correctly identified and genotyped insertions and deletions. 3. Evaluation with respect to the Tie r2 GIAB dataset. 4. Benchmarked Sniffles2 across a more challenging SV dataset across 386 medically relevant, but highly polymorphic/challenging, genes. 5. Insertion sequence identity. 6. Detection of large insertions. 7. Performance of Sniffles2 and other tools to three T2T assemblies using dipcall. 8. Benchmark of duplications, inversions and translocations using simulated data. 9. Timing of merges of three to 777 samples. 10. Mendelian consistency test for three merge strategies. 11. Mendelian consistency across seven complete families (proband, mother and father). 12. Benchmark of mosaic SV calling. 13. Number of detected germline and mosaic SVs for the MSA sample. 14. Thirty-four candidate mosaic SVs from the MSA sample. 15. Curated list of the 2,856 mosaic SVs that overlapped with 1,176 genes, including some that are related to the brain and its development. 16. Comparison of germline and mosaic SVs with different repeat families. 17. Agreement of all SVs detected in the same sample by the three technologies for a minimum length of 50 bp. 18. Sniffles2 germline and mosaic SV calls against a COLO829 benchmark dataset. 19. GIAB benchmark divided by SV length for ONT and HiFi data. 20. Software used.

## Data Availability

GIAB HG002 PacBio HiFi data are hosted at the GitHub server: https://ftp-trace.ncbi.nlm.nih.gov/ReferenceSamples/giab/data/AshkenazimTrio/HG002_NA24385_son/PacBio_CCS_15kb/. ONT HG002: https://labs.epi2me.io/gm24385_q20_2021.10/ ONT HG00733: https://www.internationalgenome.org/data-portal/search?q=HG00733 and https://ftp.hgsc.bcm.edu/Software/Truvari/3.1/sample_vcfs/hg19/li/HG00733.vcf.gz GIAB benchmark sets: Genome wide: https://ftp-trace.ncbi.nlm.nih.gov/ReferenceSamples/giab/release/AshkenazimTrio/HG002_NA24385_son/NIST_SV_v0.6/ Medical regions: https://ftp-trace.ncbi.nlm.nih.gov/ReferenceSamples/giab/release/AshkenazimTrio/HG002_NA24385_son/CMRG_v1.00/ The 1000 Genomes datasets of the three genomes were downloaded from https://github.com/human-pangenomics/hpgp-data. The dipcall results that we leveraged as benchmarks are deposited at https://github.com/smolkmo/Sniffles2-Supplement. The other datasets have been made available in the Sequence Read Archive (SRA). Thirty-one ONT datasets that represent cases of Mendelian disorders have SRA bioproject ID PRJNA953021 and database of Genotypes and Phenotypes (dbGaP) ID phs002999.v1.p1. MSA sample has bioproject ID PRJNA985263. The COLO829BL (normal) and COLO829 (tumor) ONT samples can be found with European Nucleotide Archive (ENA) ID PRJEB27698 (samples ERR2752451 and ERR2752452, respectively), and the Revio tumor/normal samples can be found at https://downloads.pacbcloud.com/public/revio/2023Q2/COLO829/. The individual VCF files for Sniffles across the samples that are publicly available (not dbGaP) can be found at 10.5281/zenodo.8144524. All software used (with versions) is listed in Supplementary Table 20.

## Code availability

Source code for Sniffles2 is available at https://github.com/fritzsedlazeck/Sniffles and 10.5281/zenodo.8121996. The auxiliary scripts are available at https://github.com/smolkmo/Sniffles2-Supplement and 10.5281/zenodo.8122060.
