# Publisher Correction: Detection of mosaic and population-level structural variants with Sniffles2

**DOI:** 10.1038/s41587-024-02141-2

**Published:** 2024-01-22

**Authors:** Moritz Smolka, Luis F. Paulin, Christopher M. Grochowski, Dominic W. Horner, Medhat Mahmoud, Sairam Behera, Ester Kalef-Ezra, Mira Gandhi, Karl Hong, Davut Pehlivan, Sonja W. Scholz, Claudia M. B. Carvalho, Christos Proukakis, Fritz J. Sedlazeck

**Affiliations:** 1 Human Genome Sequencing Center Baylor College of Medicine, Houston, TX USA; 2 Department of Molecular and Human Genetics, Baylor College of Medicine, Houston, TX USA; 3 Department of Clinical and Movement Neurosciences, Royal Free Campus, Queen Square Institute of Neurology, University College London, London, UK; 4 Aligning Science Across Parkinson’s (ASAP) Collaborative Research Network, Chevy Chase, MD USA; 5 Pacific Northwest Research Institute (PNRI), Seattle, WA USA; 6 Bionano Genomics, San Diego, CA USA; 7 Division of Neurology and Developmental Neuroscience, Department of Pediatrics, Baylor College of Medicine, Houston, TX USA; 8 Neurodegenerative Diseases Research Unit, National Institute of Neurological Disorders and Stroke, Bethesda, MD USA; 9 Department of Neurology, Johns Hopkins University Medical Center, Baltimore, MD USA; 10 Department of Computer Science, Rice University, Houston, TX USA

**Keywords:** Genome informatics, Genetics, Cancer, Software

Correction to: *Nature Biotechnology* 10.1038/s41587-023-02024-y, published online 2 January 2024.

In the version of the article initially published, Supplementary Tables 1–19 were missing. They are now included in the Supplementary Information.

