# Supplementary material for: Detection of mosaic and population-level structural variants with Sniffles2
Source: Nat Biotechnol. Author manuscript; Available in PMC 2024 Oct 14. (PMC11217151; doi:10.1038/s41587-023-02024-y)
Supplement: Supp_Information [file NIHMS1969667-supplement-Supp_Information.pdf]

Supplementary Section 1: GIAB and HGSC benchmarking

Across the default coverage (30x for HiFi, 50x for ONT), Sniffles2 shows the best performance with respect to correctly identified and genotyped insertions (HiFi: GT F-score 0.909, ONT: GT F-score 0.915) and deletions (HiFi: GT F-score 0.934, ONT: GT F-score 0.944) (see **Supplementary Table 2** for details). Sniffles2 achieves a better result in a fraction of the time across data sets compared to Sniffles (v1.12), being over 17 times (HiFi) and 11 times (ONT) faster in processing a 30x coverage data set, respectively. **Figure 2A+B** shows the results for PacBio HiFi and ONT, respectively. In addition, Sniffles2 is also the fastest method overall, requiring 33.33 CPU minutes for processing a 30x coverage HiFi dataset , which was twice as fast as SVIM, the 2nd fastest method. For a 30x coverage ONT dataset, Sniffles2 was also close to twice as fast (1.92x) as the second fastest caller (SVIM), while also having an over 8.4% higher GT F-score. Taking into account Sniffles2 multi-processing capability (not supported by SVIM), the speedup increases even further, to more than 5.4-fold and 7.5-fold for HiFi 30x, ONT 30x data sets, respectively. When reducing the coverage from 30x to 10x we observe only a slight reduction in F-score for Sniffles2 (HiFi: reduction GT F-score 0.041, ONT: reduction F-score 0.058). This is in stark contrast to other programs such as cuteSV, where using default parameters, F-score dropped by an average of over 60% (HiFi: reduction F-score 0.56, ONT: reduction F-score 0.58). Even when using only 5x for Sniffles2, we still observe a high accuracy ONT (F-score: 0.74) and HiFi (F-score: 0.77). This is achieved as Sniffles2 includes an automated parameter selection for filtering of SV candidates based on the available coverage. In contrast, other SV callers rely on manual adjustment of these parameters to retrieve acceptable results across coverages and sequencing technologies. **Figure 2A-D** shows this clearly as all other SV callers show a decreased performance across lower coverage. Even

when tuning the parameters for other SV callers (**Figure 2A-D,** Tuned parameters), Sniffles2 remains the highest accuracy (see **Supplementary Table 1** for details).

Additionally, we tested Sniffles2 with an additional mapper (LRA) to showcase its versatility. When mapping HiFi data with LRA we observe a slight increase in performance when comparing the genotype F-scores (HiFi GT F-Score increase by 0.006), moreover when using LRA to map ONT data we observe a slight decrease in performance of Sniffles2 (ONT GT F-Score decrease 0.0136). Interestingly, wWhen testing Sniffles with CLR data the error rates of this outdated data impacts our variant calling and thus we suggest other SV callers (see **Supplementary Table 1**).

Next, we performed an evaluation with respect to the Tier2 GIAB dataset, which is a more challenging region of the GIAB benchmark set as it includes repeats and GIAB cannot guarantee the accuracy of the variants in these regions (see **Supplementary Table 3** for details). Again, Sniffles2 even increases the performance difference compared to other SV caller. Lastly, we benchmarked Sniffles2 across a more challenging SV data set across 386 medically relevant, but highly polymorphic/challenging genes[33]. GIAB has recently released this call set of ~200 SV covering around 70% of these genes[33]. **Figure 2E-H** shows the results. Again Sniffles2 outperforms the other SV callers in terms of accuracy and speed using default parameters. The next best performing SV caller (pbsv for HiFi, cuteSV for ONT) both achieved 3.6% and 5.5% lower genotyping accuracy (GT F1-Score) even at 30x coverage.

Supplementary Table 4 contains the detailed results across all SV callers. Overall, Sniffles2 outperforms other state-of-the-art SV caller across the entire genome including the most challenging regions/genes.

Next, Sniffles2 improves insertion identification through two additional methods: First, the consensus module corrects sequencing-related errors in the recovered insertion sequences using a fast pseudo-alignment based approach. This allows Sniffles2 to attain the second highest mean sequence identity of (HiFi: 0.948, ONT: 0.939), after pbsv (HiFi: 0.953, ONT:

0.949), while Sniffles2 is over 14x (HiFi) and 36x (ONT) faster (see **Supplementary Figure 1, Supplementary Table 5** containing insertion sequence accuracy across all callers) at 30x coverage. Second, Sniffles2 increases the sensitivity for the detection of large insertions by recording additional supporting alignment signals in the affected regions (see **Supplementary Figure 2, Supplementary Table 6**) at much higher speed than pbsv, the only SV caller with a comparable accuracy for long insertions.

Lastly, GIAB only represents one individual benchmarked across most studies (HG002). Thus next, we used Dipcall[53] together with three T2T assemblies [54] (HG01243, HG02055, HG02080) to further assess the performance of Sniffles2. Clearly, we give Dipcall the benefit of the doubt, knowing that the accuracy will be lower than the GIAB vetted benchmark set. Overall, Sniffles2 performs the best across all samples having on average a F-Score of 0.79 at 30x coverage ONT and HiFi compared to 0.77 F-score for the next best SVcaller (cuteSV), at nearly 3.5 times the speed (74.03 CPU minutes versus 256.65 CPU minutes on average) for default parameters. **Supplementary Table 7** contains the detailed results for each benchmarked program across these three samples. Besides the here benchmarked insertions and deletions, we also benchmarked Sniffles2 on duplications, inversions and translocations using simulated data as no benchmark exists. Overall, Sniffles2 again outperformed all other methods in speed and accuracy (see **Supplementary Figure 3 and Supplementary Table 8**) (see methods for details).

Supplementary Section 2: spike in experiments for mosaic SV benchmarking

To assess the performance of Sniffles2 across mosaic SVs, we first synthetically merged HG002 (at low concentrations: 5x (7%), 7x (10%), 10x (14%), 15x (21%) or 20x (28%)) with the 50-63x coverage read data from the sample of an unrelated individual (HG00733). This yielded multiple, synthetic samples with constant total coverage of ~70x (68x in one case), but varying concentrations of HG002 in them (7-28%). We only assessed this for ONT data as this technology often is sequenced at higher sequence depth than Pacbio HiFi. The latter can still be used, but is not benchmarked here. **Figure 4 A&B** highlights the results across this synthetic data set across different concentrations of HG002 (x-axis, **Supplementary Table 12**). **Figure 4A and 4B** show the precision and recall of SVs across the different concentrations. In blue, we highlight the performance of Sniffles2 default germline-mode, which shows low precision and increased recall with increase in concentration of HG002 in the mix. In red we show cuteSV which has lower precision and recall in both cases, when compared to sniffles2 germline-mode. In yellow we highlight the performance of Sniffles2 mosaic mode. Sniffles2 achieves high precision that increases with higher proportions of HG002 (**Figure 4A**) with the highest being at 21% HG002 proportion (84.12% precision). Recall also increases with higher proportions of HG002, moreover it only reaches a maximum of 54.05%. This is because Sniffles2 mosaic-mode only calls SV where the variant allele frequency (VAF) is between 5 and 20%.. We computed the adjusted recall by only using the SVs where the VAF matches in the range that mosaic-mode uses (**Figure 4B, Supplementary Table 12**). This adjusted recall is shown in green, which averages 94.47% across all comparisons (highest 96.39% at 7% mix of HG002). Thus following the default recall for germline variants.

Supplementary Section 3: MSA analysis

We identified 3,049 Sniffles2 non-germline SVs that have read support unique to the cingulate cortex region from which 2,856 of these SV overlapped with 1,176 genes including at least 80 that are related to the brain and its development (**Supplementary Table 15**). Some examples are *GABRB3* which is implicated in many human neurodevelopmental disorders and syndromes, *NRXN3* which is involved in synaptic plasticity and Netrin receptor DCC which is involved in neuron migration and axonogenesis. *GRIN2A*, which encodes for NMDA receptor subunit 2a. Of these 29 SVs, 27 overlapped introns and 2 affected at least one exon. Furthermore, 4 SVs disturb regulatory regions associated with at least one gene, including *PEX26*, *DLL1* and *ABCA2*. This further highlights Sniffles2's ability to not only detect SVs that cannot be identified using Illumina data alone but also the likely unique presence of a subset of these calls within a brain region.

As highlighted above the role of repeat elements (Alu/L1) are important for the formation of germline and non-germline SV (**Supplementary Table 16**, **Figure 5C**). In summary, we found a similar fraction of germline and non-germline SVs was associated with repeat elements (60.61% and 59.70% respectively). When comparing specific repeat elements, we found Alu being more abundant in germline SV (4.82% difference) and simple repeats more abundant in non-germline/mosaic SVs (6.54% difference). The patterns of repeat association only shifted in duplication where LTR, L2 and other repeats differed from the norm and with inversions where no SV was detected in the mosaic SVs (**Figure 5C** and **Supplementary Figure 9**). For duplications, the fraction of non-germline SVs associated with LINE1 and simple repeats showed the highest difference over germline SVs. This highlights the different ways in which repeat elements are associated with somatic structural variants. As we could show above Alu insertion mediated mosaic deletions. **Extended Figures 3** and **4** shows two examples of read

alignments for a non-germline duplication SV that was solely called by Sniffles2, alongside with its relation to nearby and overlapping repeat elements. For deletions and insertions, we observe similar to slightly lower fractions of non-germline SVs associated with most repeat types, with the exception of simple repeats. For this repeat family, especially non-germline duplication and insertion SVs had a higher fraction associated. Repetitive elements may be associated with neurodegenerative disorders, through increased expression and / or de novo somatic genomic integration[41]. The observance of a higher fraction of non-germline insertion and deletion SVs being associated with simple repeat elements could suggest a further correlation for this on the level of an individual brain region. Overall, this also highlights the differences between repeat families in their effects on somatic SV generation[63].

Supplement Section 4: Technology comparison for Brain MSA sample

From the total of 22,619 merged SV calls, only 214 are shared across all methods and 19,254 (85.12%%) were detected by Sniffles2 together with either Manta, OGM or both. Sniffles2 uniquely detected 10,720 (47.39%) SVs, while Manta uniquely detected 2,599 (11.49%) and OGM 749 (3.31%). We next identified the most abundant SV type per method for the unique calls. We found that Sniffles2 has a higher number of insertions and deletions (94.87%, 70.98% respectively%), while for Manta are duplications and inversion (98.35%, 92.12% respectively, **Supplementary table 17.2**,). At the same time, Illumina showed a higher overlap with Sniffles2 (8,022 SVs) compared to OGM (298 SVs), likely also because of the minimum length for OGM. The overlap between Sniffles2 and the other technologies was also much higher for deletions (4,801) than for insertions (3,607). These differences are likely explained by the individual difficulty in detecting larger (i.e., Illumina) and smaller (i.e., OGM, recommended threshold approximately 5kbp or larger) insertion events, respectively. Next, we took a closer look at putatively mosaic SVs detected by Sniffles2. For this, we separated Sniffles2 detected SVs by their reported variant allele frequencies (VAF) into germline (VAF > 0.3) and likely mosaic (VAF

≤ 0.2) calls. A total of 58 insertions were shared uniquely by the germline and mosaic calls of Sniffles2. No other SV types were observed overlapping between mosaic and germline. Interestingly wWe do observe size differences for these 58 SV but share the same start location.. Next, we compared the mosaic Illumina and OGM calls. Only 702 mosaic SVs reported by Sniffles2 could be detected by either Illumina or OGM or both (24 OGM, 666 illumina and 12 both), highlighting the difficulty in identifying rare SVs. For deletions, only 473 of SVs were also found by either OGMand/or Illumina. Only 218 mosaic insertions reported by Sniffles2 were detected by the other methods. For duplications and inversions, only the Illumina data showed overlap (one and ten, respectively) with Sniffles2 mosaic SVs.

We further noted a shift in the allele frequencies across the Manta calls compared to the Sniffles2 calls **(Figure 5B)**. As expected, for Sniffles2, we observe a multimodal distribution with three peaks, representing homozygous, heterozygous and non-germline SVs, respectively. In contrast, Manta shows two main peaks in their allele frequency distribution. A homozygous (~0.9-1 AF) and a broad peak around 0.3 AF, which would be below the typical expected heterozygous AF peak. For Sniffles2, we furthermore observe a ~140% increase in the area under the curve in the putative non-germline range of allele frequencies (0.0-0.2) when compared to the illumina data, thus showing the potential of Sniffles2 to detect low frequency SV.

Supplementary Section 5: Identification of cancer-specific somatic SVs by Sniffles2

We have shown that Sniffles2 can accurately identify SVs across the whole range of allele frequencies. Furthermore, we showed that Sniffles2 enables fast and accurate population SV calling. Next, we made use of the population merge strategy of Sniffles2 to investigate its ability to identify tumor-specific somatic SVs using paired tumor/normal samples. For this endeavor we used the well-known and highly studied COLO829 cancer cell line (with blood control COLO829BL). By merging the SV calls from both tumor and normal we are able to identify tumor-specific somatic SVs based on the genotype, alternative read count and and the support vector. **Figure 5D** shows shared and unique SV between the COLO829/COLO829BL cell-lines for two long-read technologies: ONT MinION and Pacbio Revio.

First, we tested both germline and mosaic SV calls against a COLO829 benchmark dataset[66]. This benchmark was done by analyzing the COLO928 sample using multiple technologies. We filtered out all SV smaller than 50 bases, and divided the analysis in two parts: translocations (13 BNDs in the benchmark) and the rest of the SVs (49 SVs in the benchmark). For the case of the translocations (BND) Sniffles2 reported five BND, from which four are present in the benchmark with all within five bases of either breakpoint. Nine BNDs from the benchmark were not detected by Sniffles2, and one BND was called but not present in the benchmark (**Supplementary Table 18**). For the rest of the 49 SVs (INS, DEL, INV, DUP), Sniffles2 was able to identify 36 in either the ONT, Revio datasets or both with breakpoints within 300 bases (max. allowed distance between breakpoints = 1kb, **Supplementary Figure 10**), moreover in some cases the technologies didn't agree on the genotype (het vs. homozygous alt.). This highlights potential changes across the cell lines as the ONT and Pacbio data were released a few years apart. From the remaining unidentified SV, four had no reads for the alternative allele (0% AF), three were identified by Sniffles as mosaic (8.2-21% AF), four had heterozygous genotype

(31-41% AF) and two had homozygous alternative genotype (81-100% AF, **Supplementary table 18, Supplementary figure 12).** For Sniffles2 it shows that we missed only six germline and three mosaic SVs from the benchmark (81.63% recall, **Supplementary table 18**). These results show the difficulty of defining a benchmark dataset for an ever-evolving cancer cell-line such as COLO829 or any other.

We further annotated 79 tumor specific somatic SVs identified by Sniffles (see Methods). These SVs overlap with cancer related genes such as *PTEN, PMS2, ARHGEF5, PAK2, WWOX* to mention some. Additionally, some SVs overlap with olfactory receptor pseudogenes. **Figure 5E** and **Supplementary Figure 11** shows examples of cancer-specific germline SV. Manual inspection in IGV identified homozygous, heterozygous and LoH events.

Supplementary figures

A)

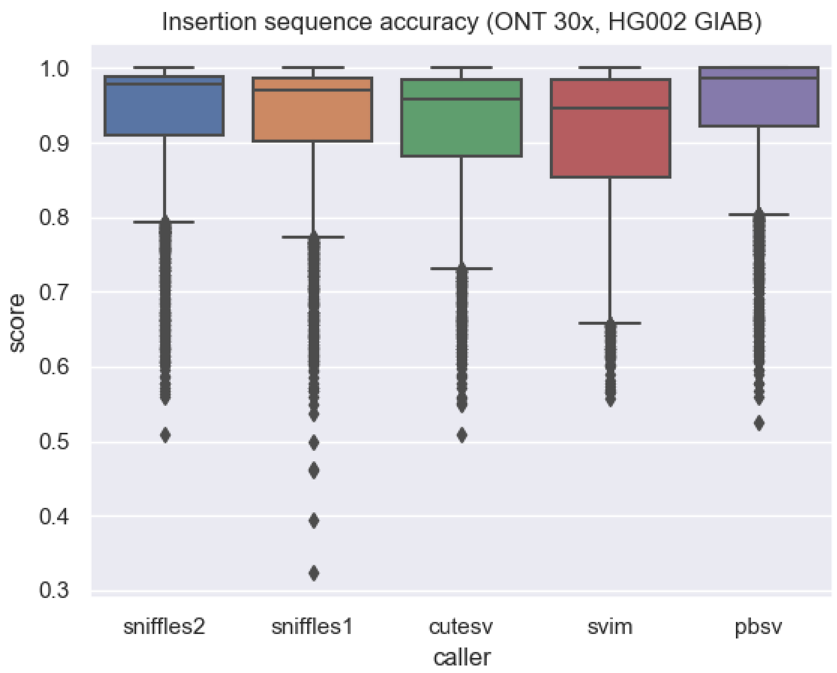

B)

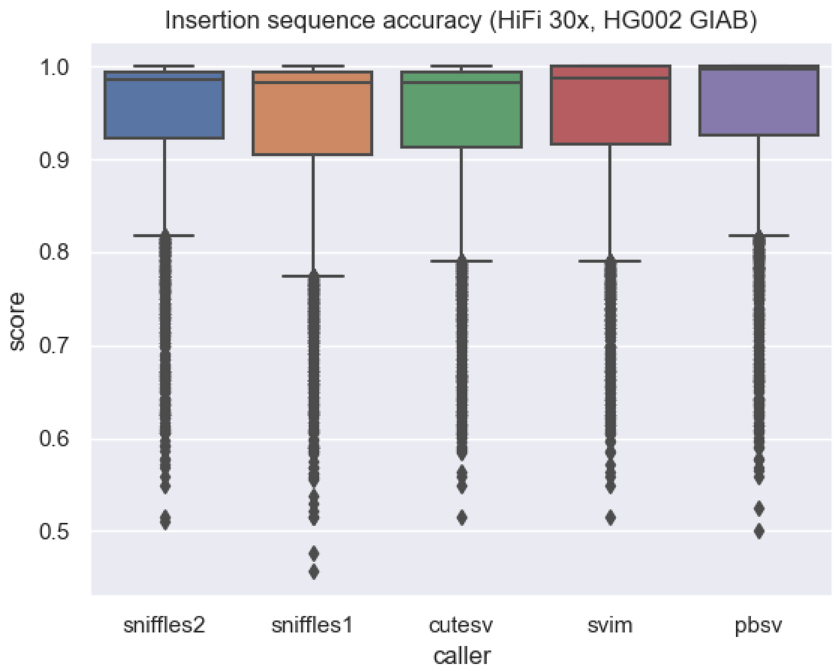

Supplementary Figure 1

Accuracy of recovered insertion sequences. All callers were benchmarked using default parameters as described in the methods section on benchmarking methodology. For ONT the number of insertions (INS) analyzed is 3,048 for every tool, which is the intersection of all TP from all callers. For the case of PacBio HiFi, the number of INs is 3440 for every tool, following the same strategy as in ONT.

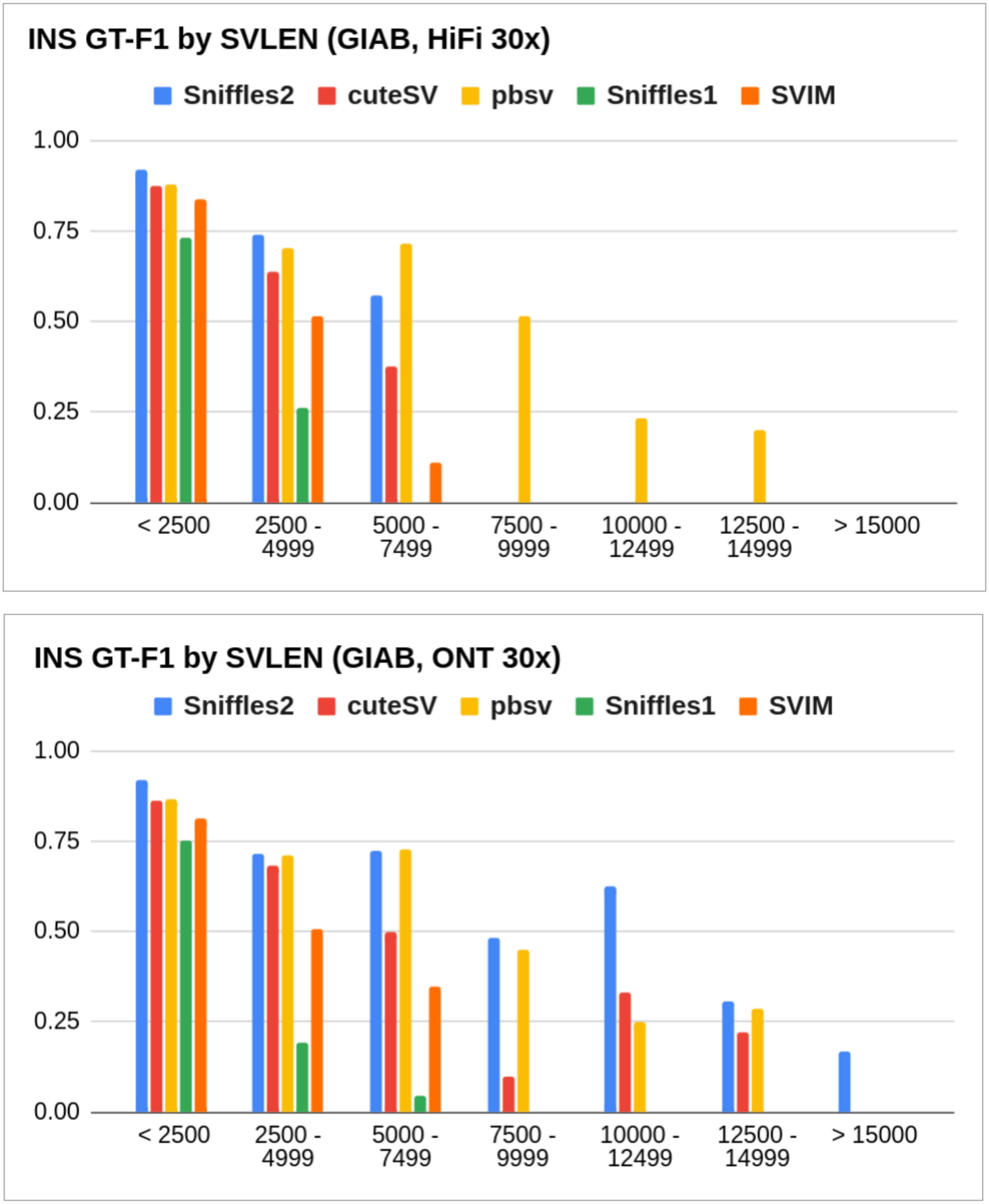

Supplementary Figure 2

Genotype F1-measure for the detection of insertions by SV length. Measured by using the Truvari summary output files for the evaluation of each caller on the GIAB Pacific Biosciences HiFi and Oxford Nanopore 30x coverage benchmark. All callers were benchmarked using

default parameters. For those cases that no data is shown means that the result was not defined (NA). **Supplementary Table 6** also shows the number of SVs tested by SV length (Insertions only)

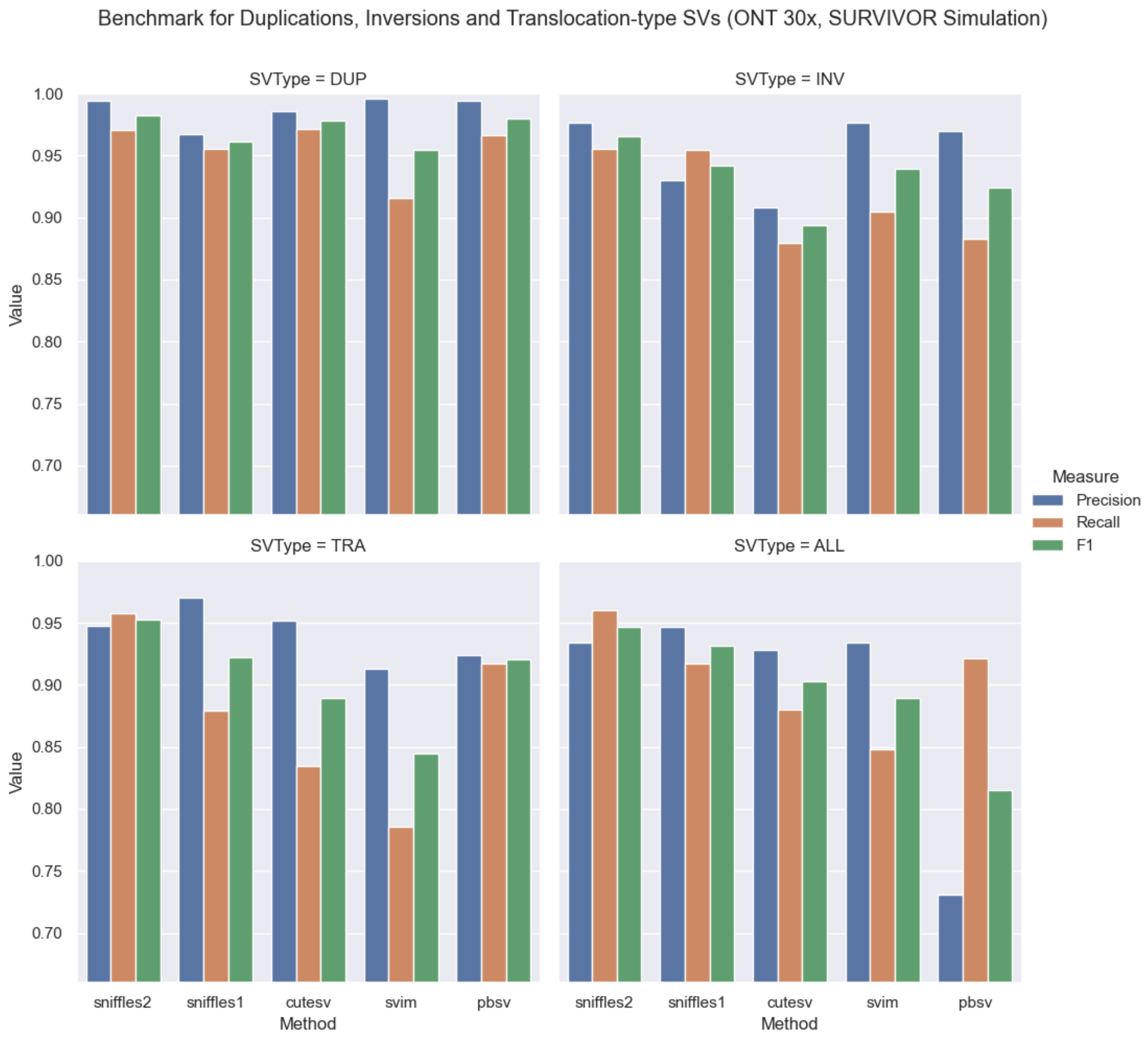

Supplementary Figure 3

Benchmark for duplications, inversions and translocations (simulation generated and evaluated using SURVIVOR). Precision, recall and F-measure were calculated from the SURVIVOR eval command output. All callers were benchmarked using the default parameters described in the methods section on benchmarking methodology.

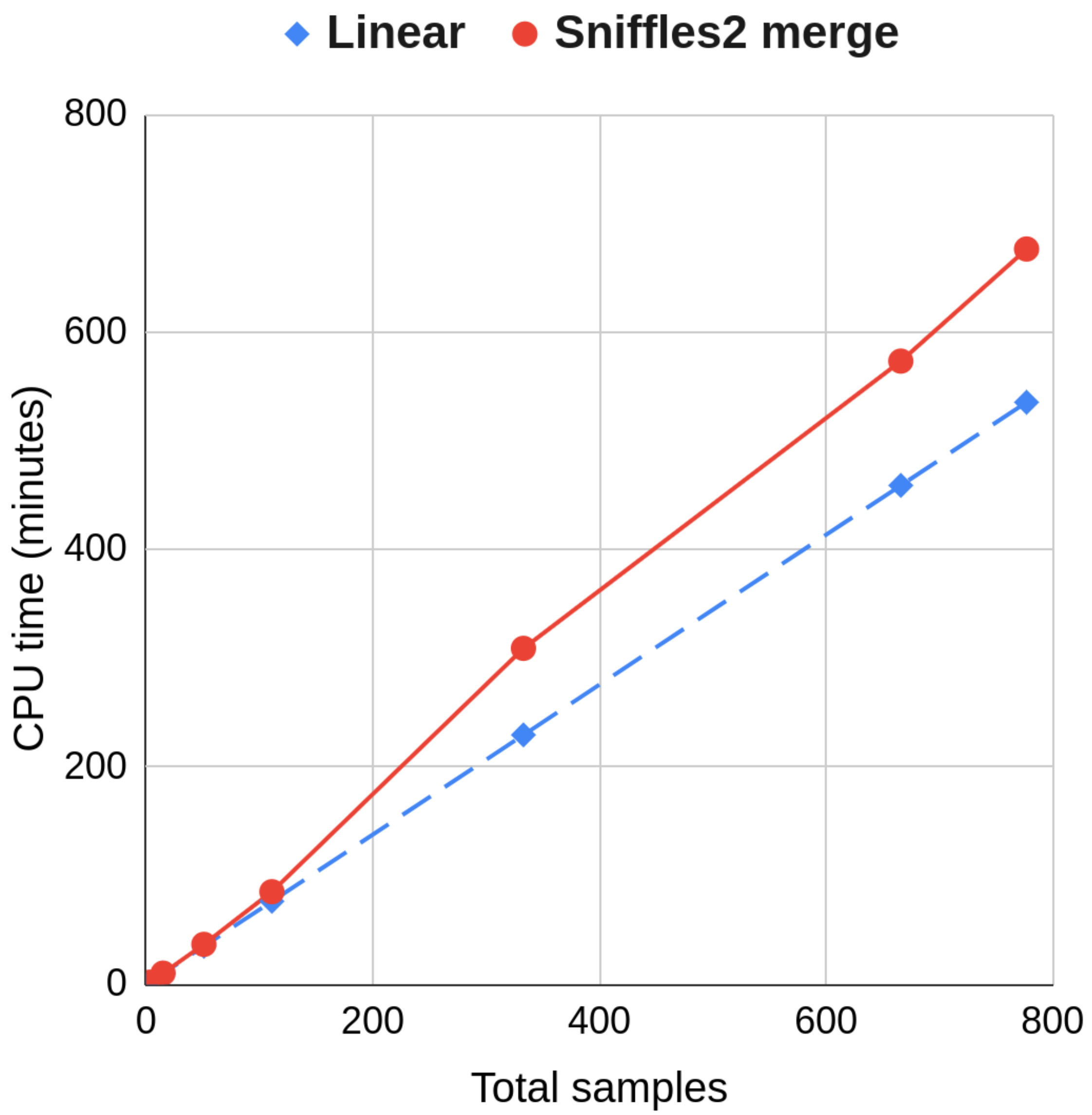

Supplementary Figure 4

Performance of Sniffles2 population / multi-sample merge as a function of the number of samples combined. Performance is near-linear for the shown range of 3 to 777 samples. As input data, multiples of the HG002, HG003 and HG004 trio were merged (representing 1 to 259 families).

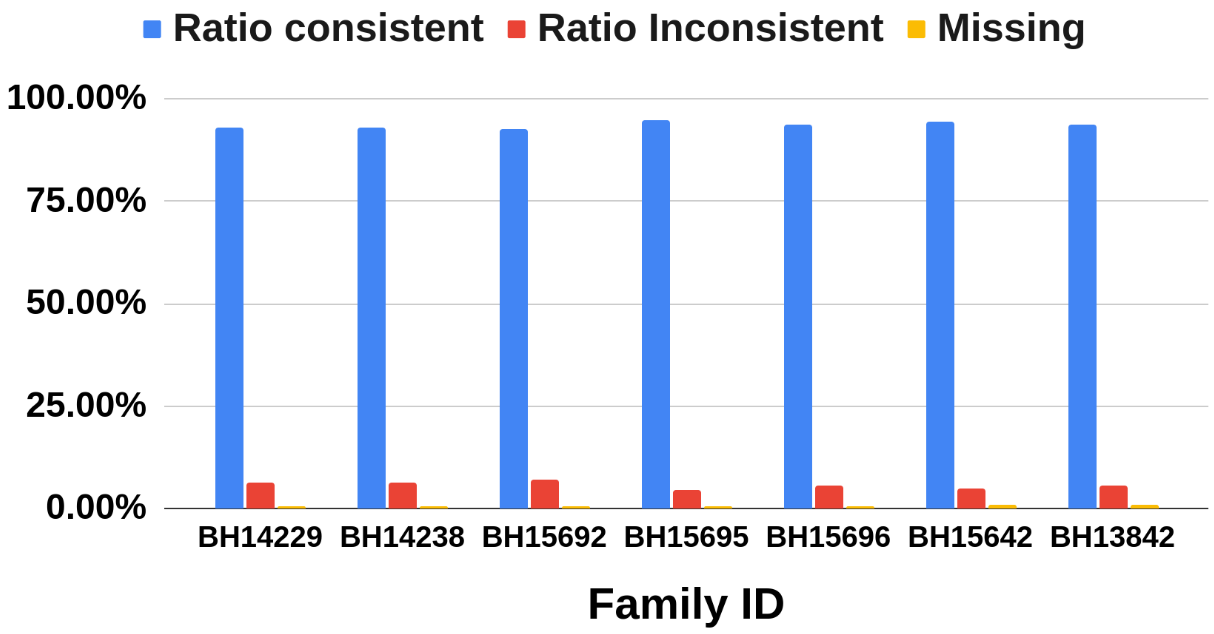

Supplementary Figure 5

Mendelian consistency, inconsistency and missing genotypes (NA) from a fully genotyped population VCF of 31 genomes. We show the information of 7 complete families (father, mother, proband, 21 genomes). We measured an average of 5.82% Mendelian inconsistency rate and 0.56% of missingness across the seven families (See **supplementary table 11**).

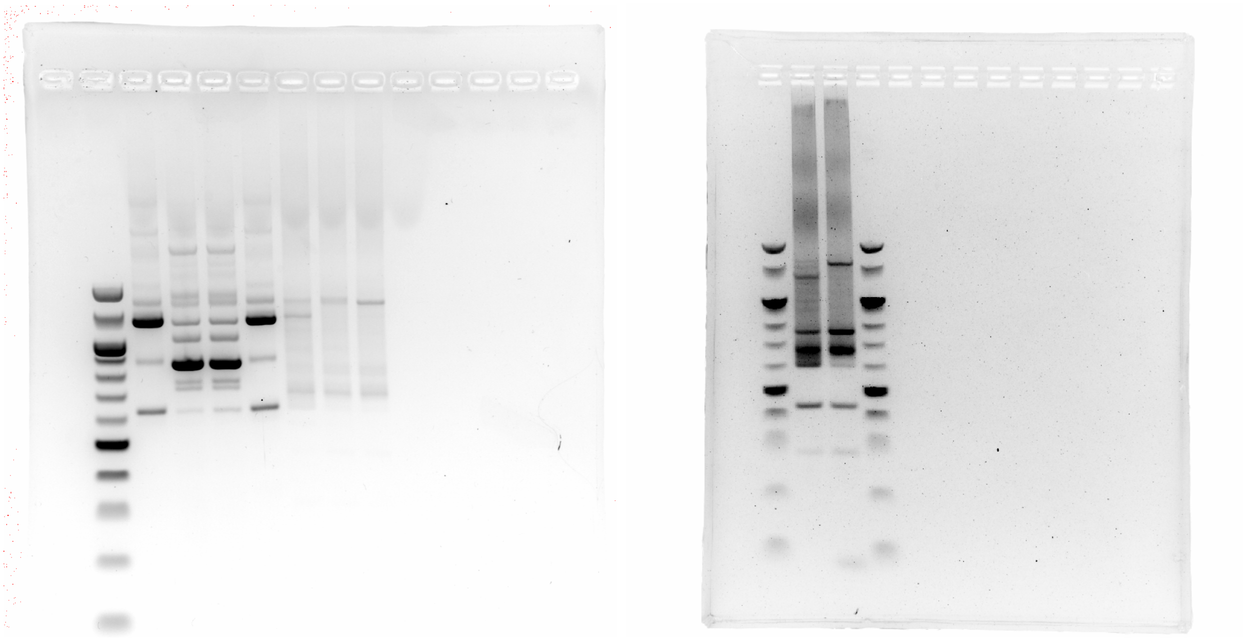

Supplementary Figure 6

Complete PCR gel confirmation of the two mosaic SVs shown in **Figure 4D** and **4E**.

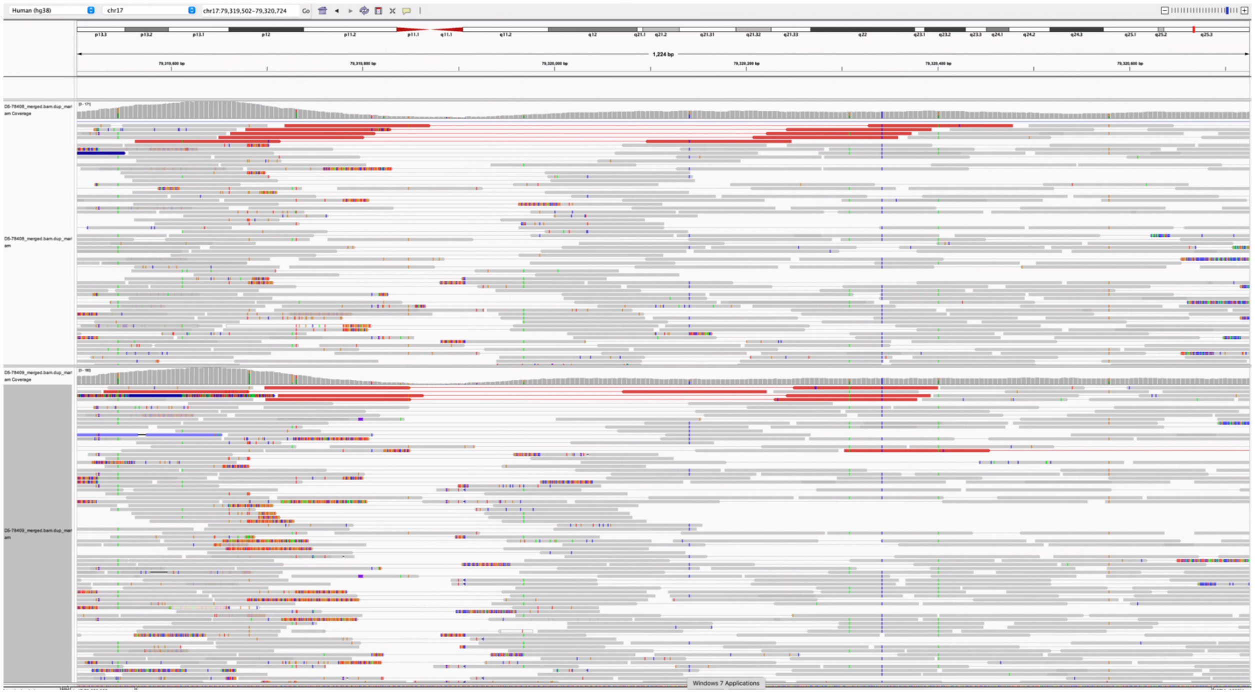

Supplementary Figure 7

IGV screenshot of a 1,224 bases overlapping a mosaic DEL shown in **Figure 4E** in chr17. Here are shown five and four read support of the mosaic SV in Illumina data for two brain regions: cingulate cortex and adjacent white matter for the same brain.

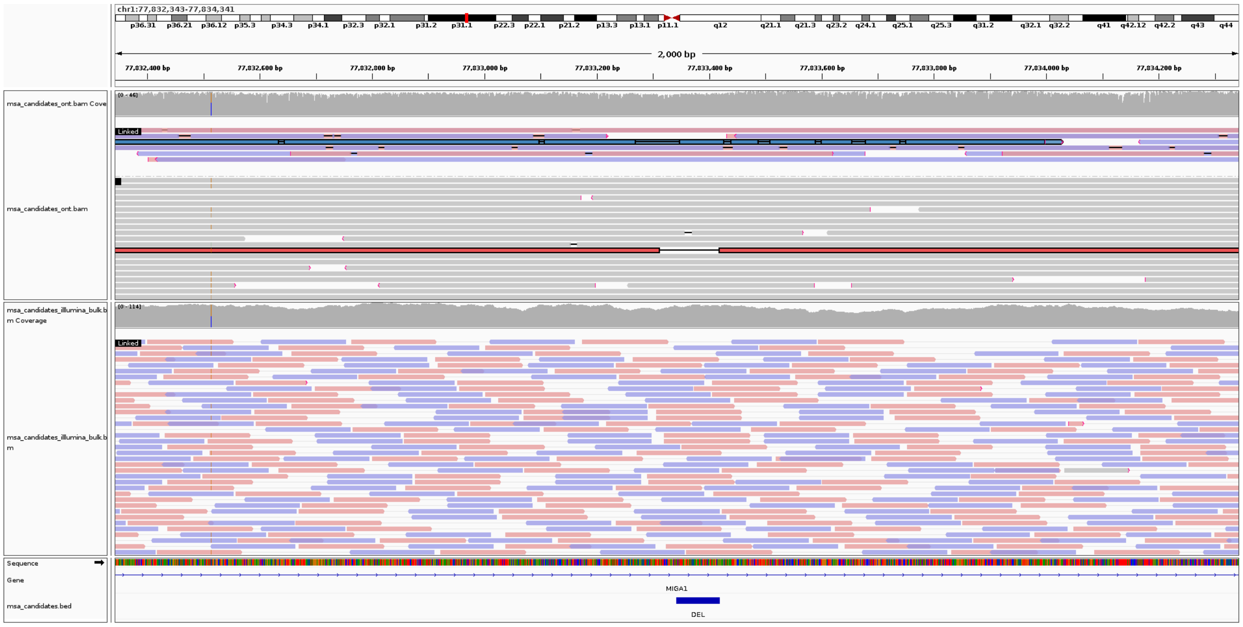
chr1:77,832,343-77,834,341
2,000 bp
msa_candidates_ont.bam Coverage
msa_candidates_ont.bam
msa_candidates_illumina_bulk.bam Coverage
msa_candidates_illumina_bulk.bam
Sequence
Gene
msa_candidates.bed
MIGA1
DEL

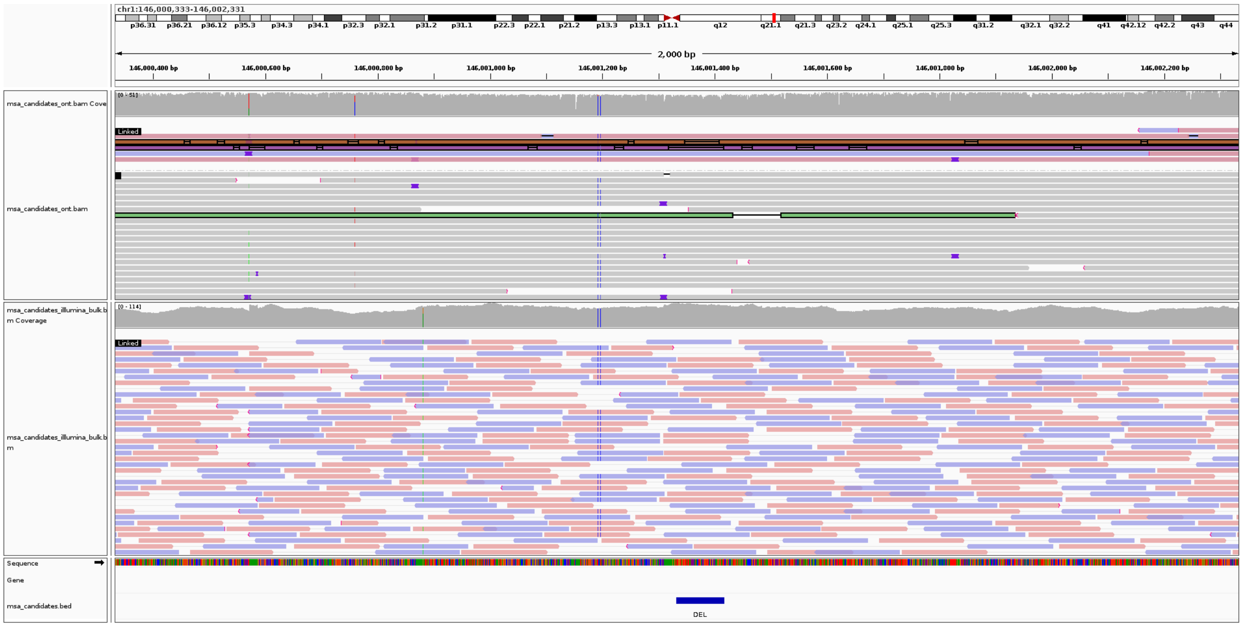
chr1:146,000,333-146,002,331
2,000 bp
msa_candidates_ont.bam Coverage
msa_candidates_ont.bam
msa_candidates_illumina_bulk.bam Coverage
msa_candidates_illumina_bulk.bam
Sequence
Gene
msa_candidates.bed
DEL

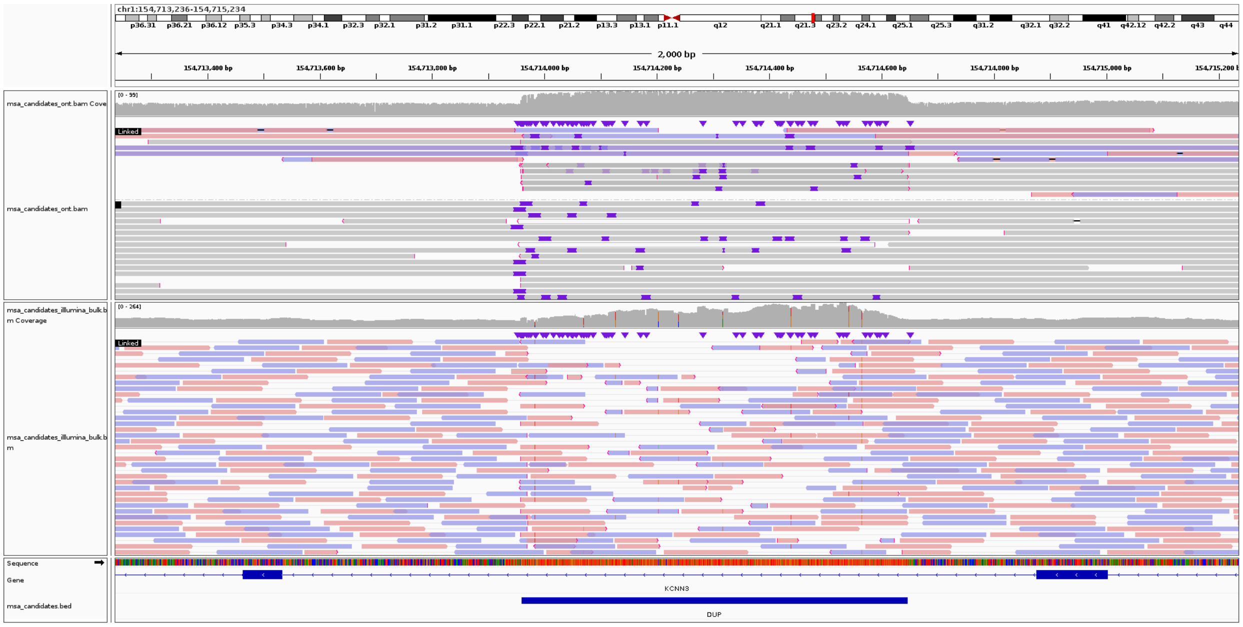
chr1:154,713,236-154,715,234
2,000 bp
msa_candidates_ont.bam Cove
msa_candidates_ont.bam
msa_candidates_illumina_bulk.bam Coverage
msa_candidates_illumina_bulk.bam
Sequence
Gene
msa_candidates.bed
Linked
KCNN3
DUP

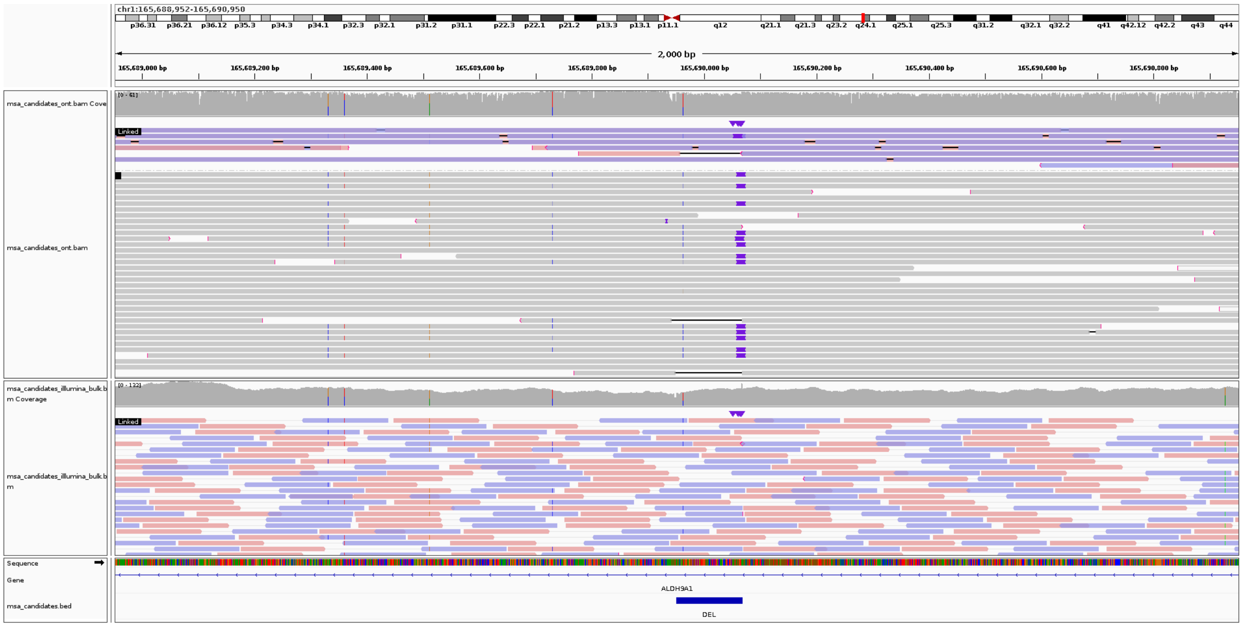
chr1:165,688,952-165,690,950
2,000 bp
msa_candidates_ont.bam Cove
msa_candidates_ont.bam
msa_candidates_illumina_bulk.bam Coverage
msa_candidates_illumina_bulk.bam
Sequence
Gene
msa_candidates.bed
Linked
ALDH9A1
DEL

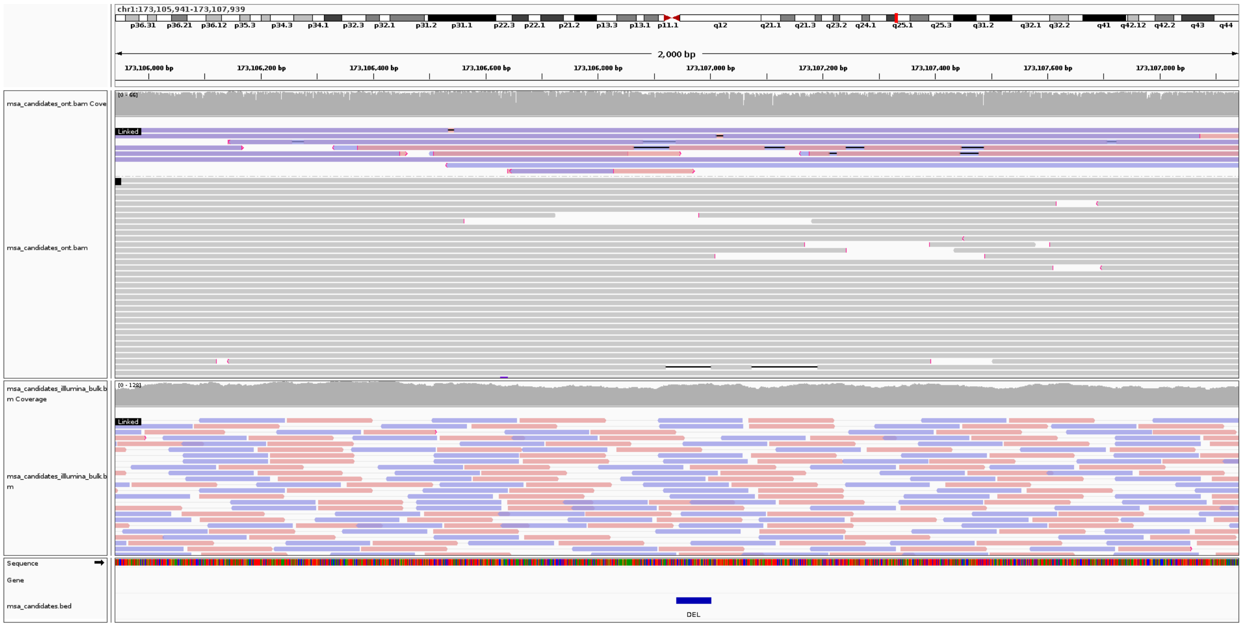
chr1:173,105,941-173,107,939
2,000 bp
msa_candidates_ont.bam Cove
msa_candidates_ont.bam
msa_candidates_illumina_bulk.bam Coverage
msa_candidates_illumina_bulk.bam
Sequence
Gene
msa_candidates.bed
DEL

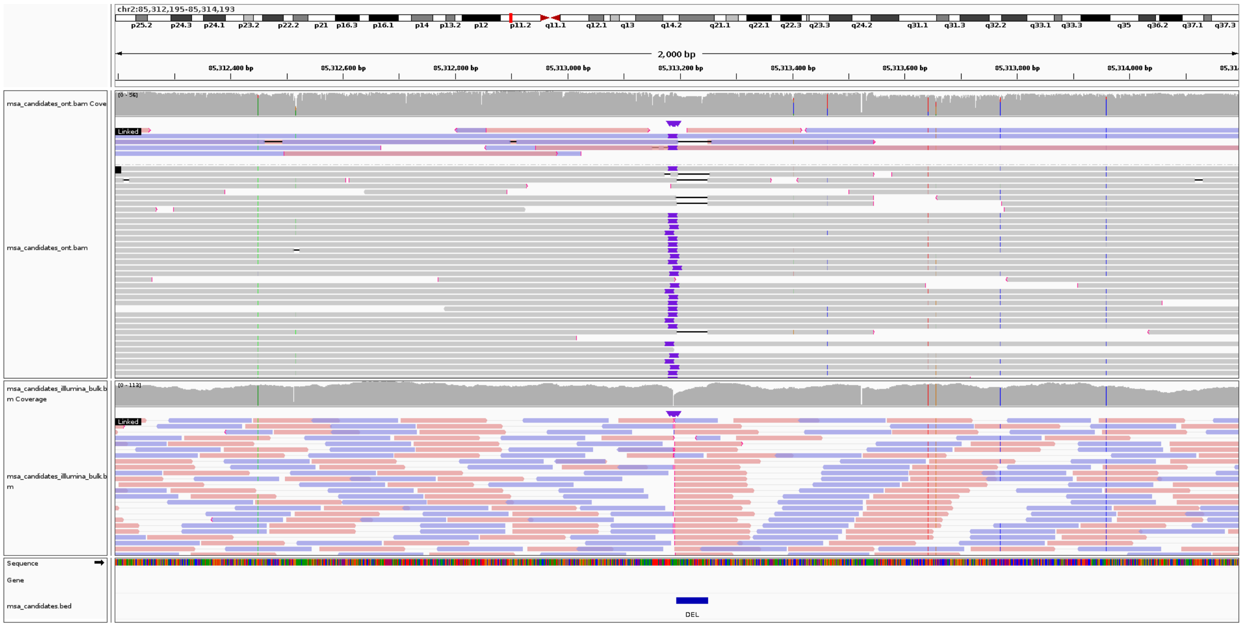
chr2:85,312,195-85,314,193
2,000 bp
msa_candidates_ont.bam Cove
msa_candidates_ont.bam
msa_candidates_illumina_bulk.bam Coverage
msa_candidates_illumina_bulk.bam
Sequence
Gene
msa_candidates.bed
DEL

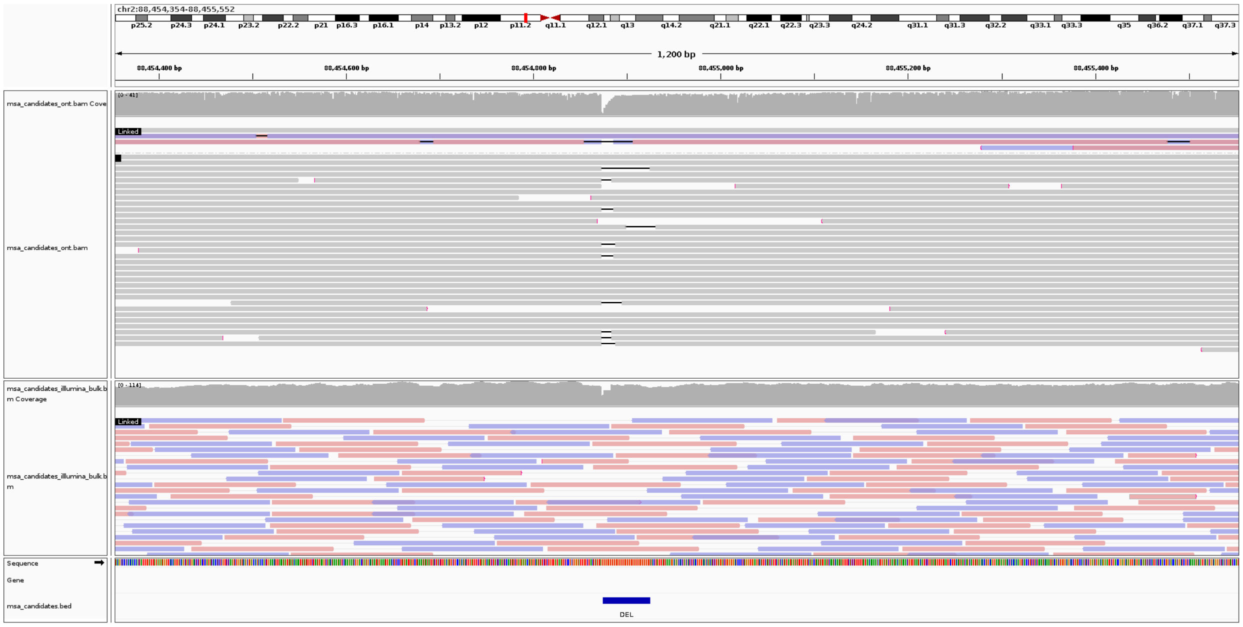
chr2:88,454,354-88,455,552
1,200 bp
88,454,400 bp
88,454,600 bp
88,454,800 bp
88,455,000 bp
88,455,200 bp
88,455,400 bp
msa_candidates_ont.bam Coverage
msa_candidates_ont.bam
msa_candidates_illumina_bulk.bam Coverage
msa_candidates_illumina_bulk.bam
Sequence
Gene
msa_candidates.bed
DEL

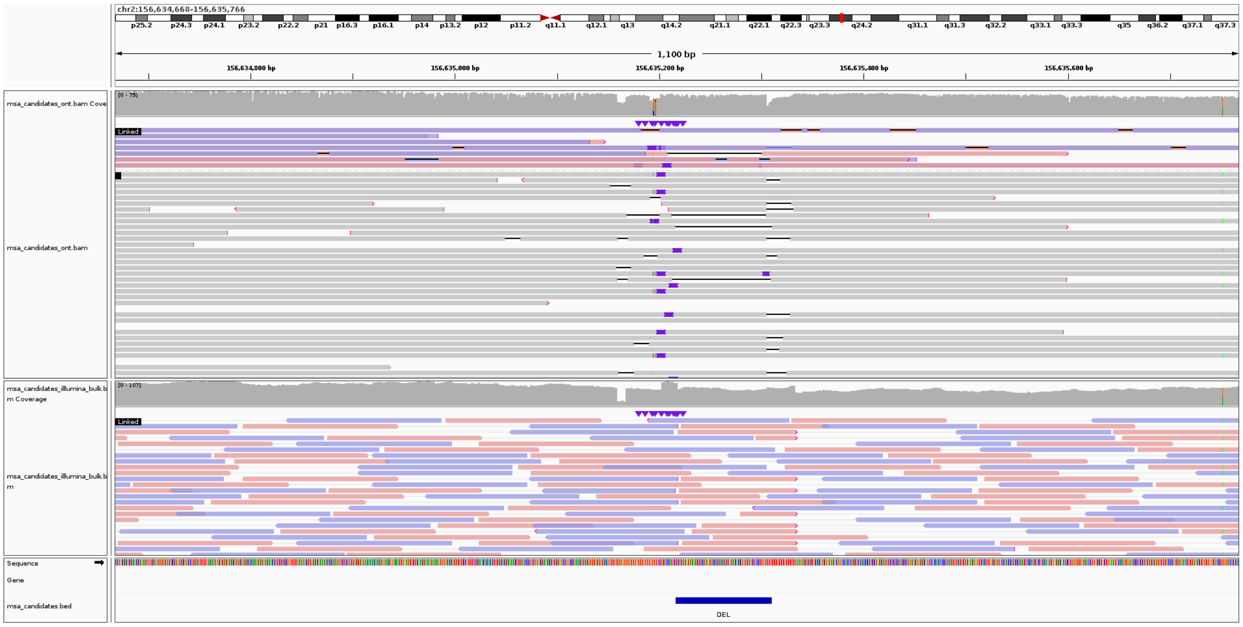
chr2:156,634,668-156,635,766
1,100 bp
156,634,800 bp
156,635,000 bp
156,635,200 bp
156,635,400 bp
156,635,600 bp
msa_candidates_ont.bam Coverage
msa_candidates_ont.bam
msa_candidates_illumina_bulk.bam Coverage
msa_candidates_illumina_bulk.bam
Sequence
Gene
msa_candidates.bed
DEL

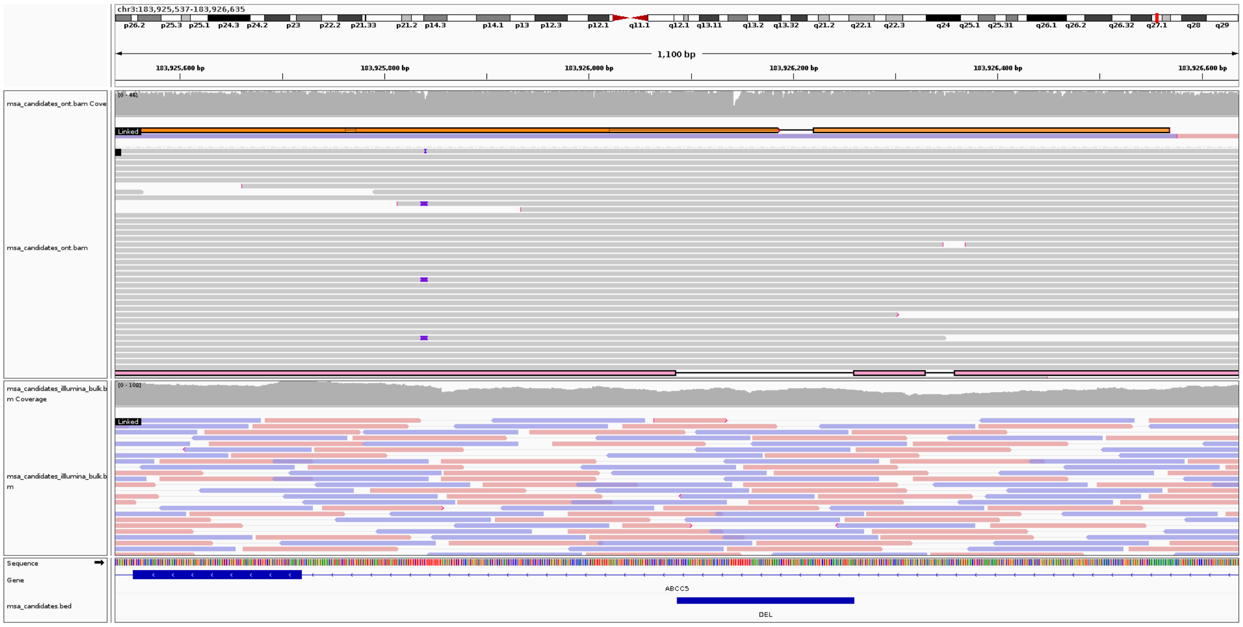
chr3:183,925,537-183,926,635
1,100 bp
183,925,600 bp
183,925,800 bp
183,926,000 bp
183,926,200 bp
183,926,400 bp
183,926,600 bp
msa_candidates_ont.bam Cove
msa_candidates_ont.bam
msa_candidates_illumina_bulk.bam Coverage
msa_candidates_illumina_bulk.bam
Sequence
Gene
msa_candidates.bed
ABCC5
DEL

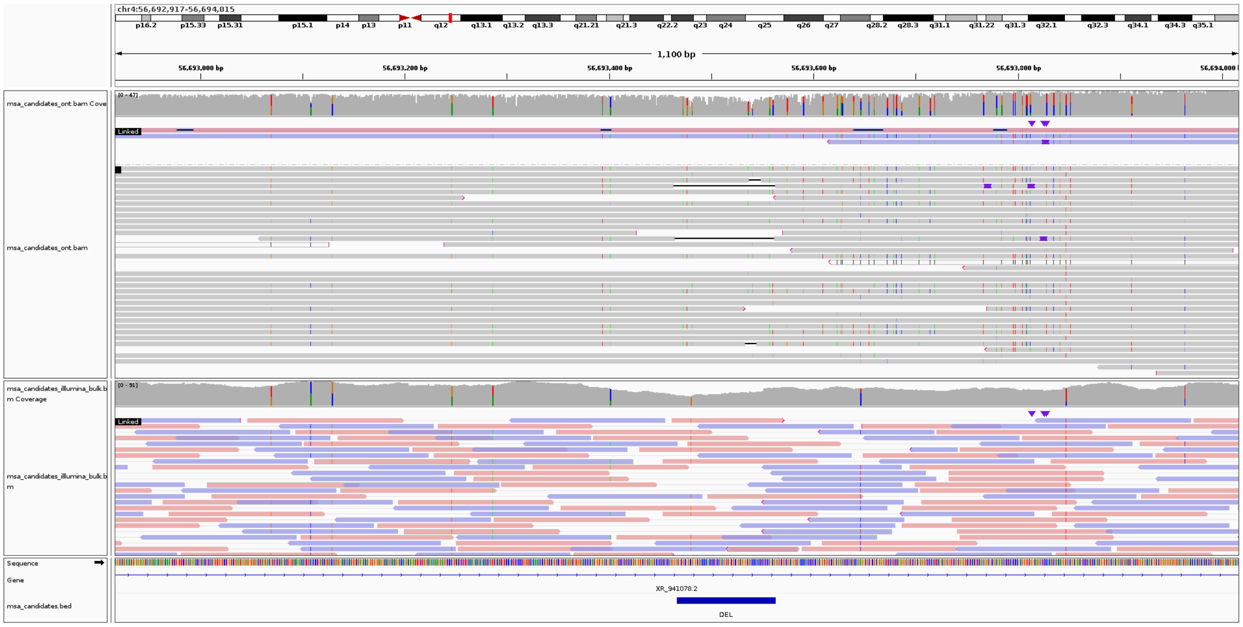
chr4:56,692,917-56,694,015
1,100 bp
56,693,000 bp
56,693,200 bp
56,693,400 bp
56,693,600 bp
56,693,800 bp
56,694,000
msa_candidates_ont.bam Cove
msa_candidates_ont.bam
msa_candidates_illumina_bulk.bam Coverage
msa_candidates_illumina_bulk.bam
Sequence
Gene
msa_candidates.bed
XR_941078.2
DEL

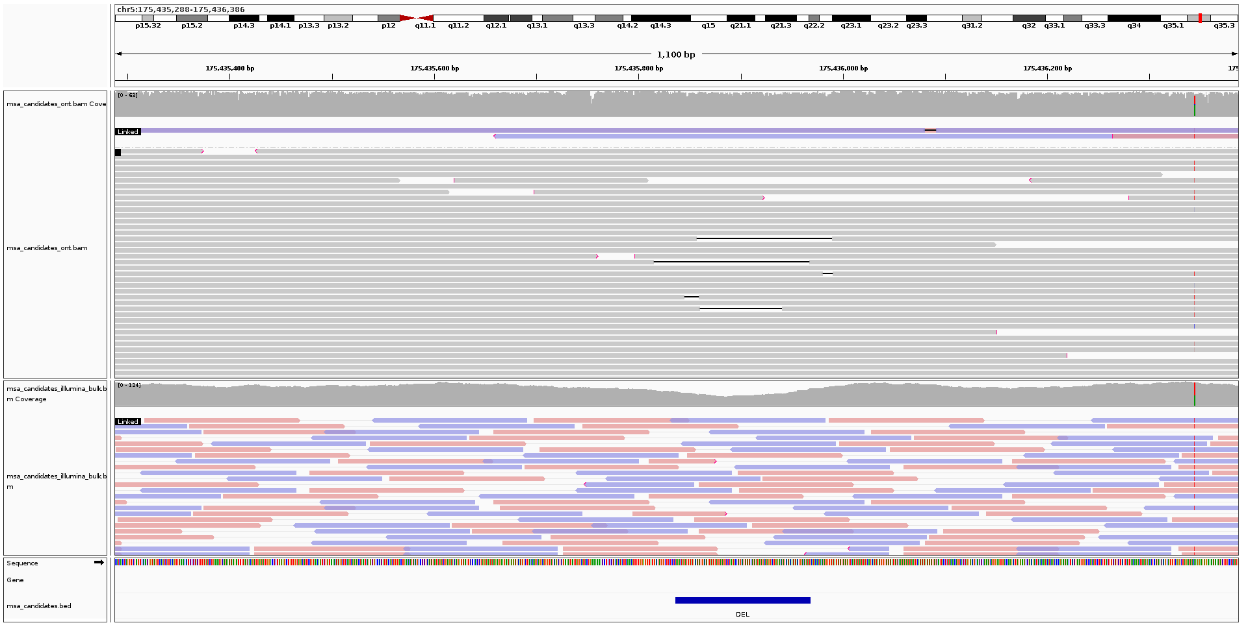
chr5:175,435,288-175,436,386
1,100 bp
175,435,400 bp
175,435,600 bp
175,435,800 bp
175,436,000 bp
175,436,200 bp
msa_candidates_ont.bam Coverage
msa_candidates_ont.bam
msa_candidates_illumina_bulk.bam Coverage
msa_candidates_illumina_bulk.bam
Linked
Sequence
Gene
msa_candidates.bed
DEL

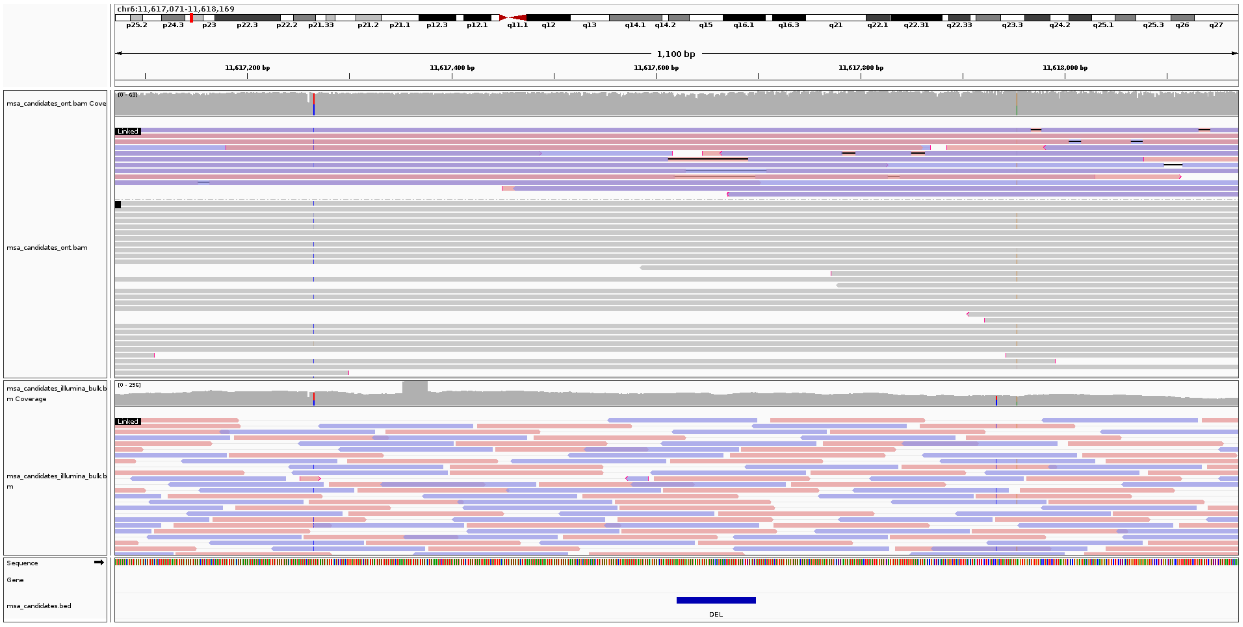
chr6:11,617,071-11,618,169
1,100 bp
11,617,200 bp
11,617,400 bp
11,617,600 bp
11,617,800 bp
11,618,000 bp
msa_candidates_ont.bam Coverage
msa_candidates_ont.bam
msa_candidates_illumina_bulk.bam Coverage
msa_candidates_illumina_bulk.bam
Linked
Sequence
Gene
msa_candidates.bed
DEL

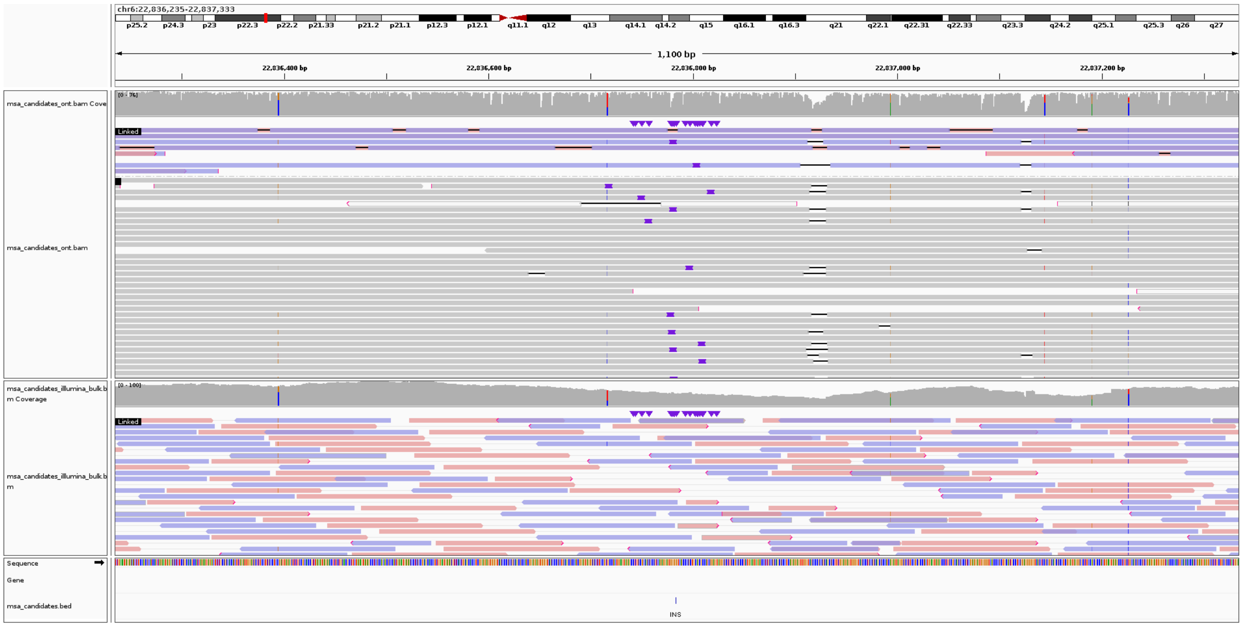
chr6:22,836,235-22,837,333
1,100 bp
22,836,400 bp
22,836,600 bp
22,836,800 bp
22,837,000 bp
22,837,200 bp
msa_candidates_ont.bam Coverage
msa_candidates_ont.bam
msa_candidates_illumina_bulk.bam Coverage
msa_candidates_illumina_bulk.bam
Sequence
Gene
msa_candidates.bed
INS

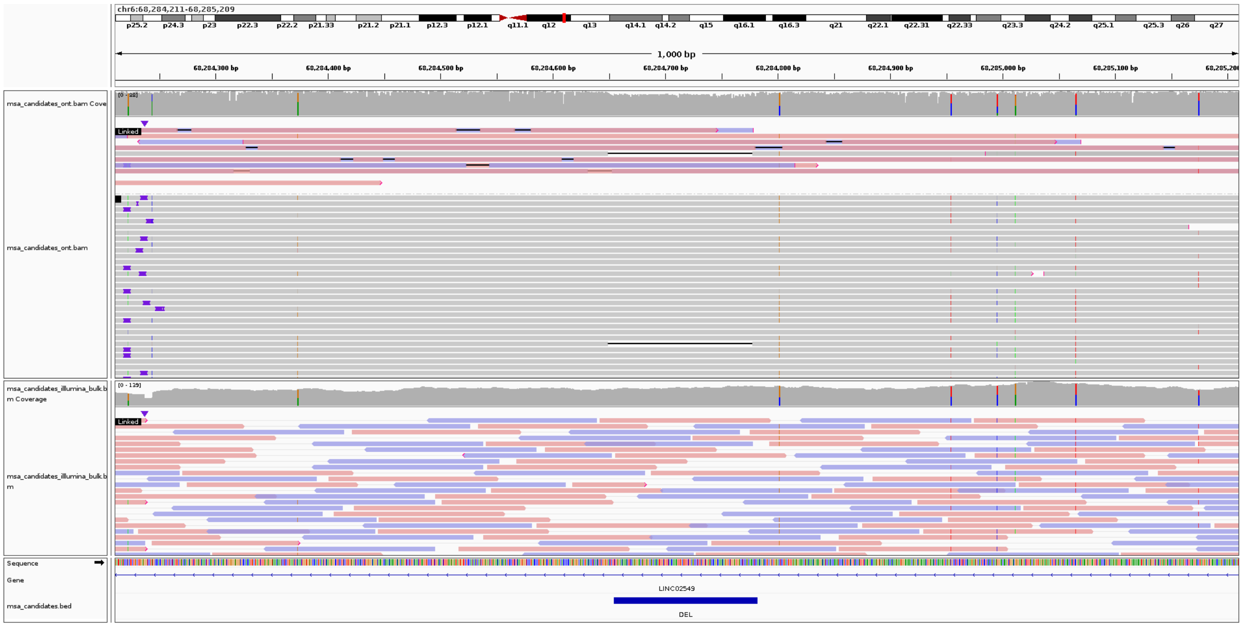
chr6:68,284,211-68,285,209
1,000 bp
68,284,300 bp
68,284,400 bp
68,284,500 bp
68,284,600 bp
68,284,700 bp
68,284,800 bp
68,284,900 bp
68,285,000 bp
68,285,100 bp
msa_candidates_ont.bam Coverage
msa_candidates_ont.bam
msa_candidates_illumina_bulk.bam Coverage
msa_candidates_illumina_bulk.bam
Sequence
Gene
msa_candidates.bed
LINC02549
DEL

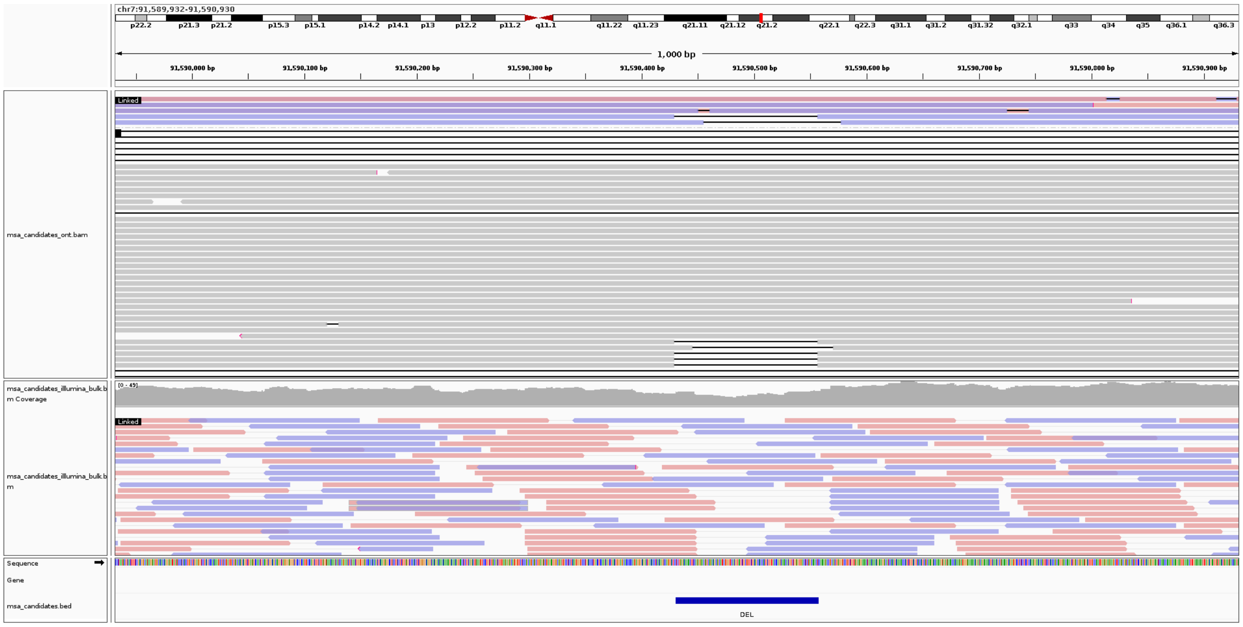
chr7:91,589,932-91,590,930
p22.2
p21.3
p21.2
p15.3
p15.1
p14.2
p14.1
p13
p12.2
p11.2
q11.1
q11.22
q11.23
q21.11
q21.12
q21.2
q22.1
q22.3
q31.1
q31.2
q31.32
q32.1
q33
q34
q35
q36.1
q36.3
1,000 bp
91,590,000 bp
91,590,100 bp
91,590,200 bp
91,590,300 bp
91,590,400 bp
91,590,500 bp
91,590,600 bp
91,590,700 bp
91,590,800 bp
91,590,900 bp
Linked
msa_candidates_ont.bam
msa_candidates_illumina_bulk.bam Coverage
[0 - 49]
msa_candidates_illumina_bulk.bam
Sequence
Gene
msa_candidates.bed
DEL

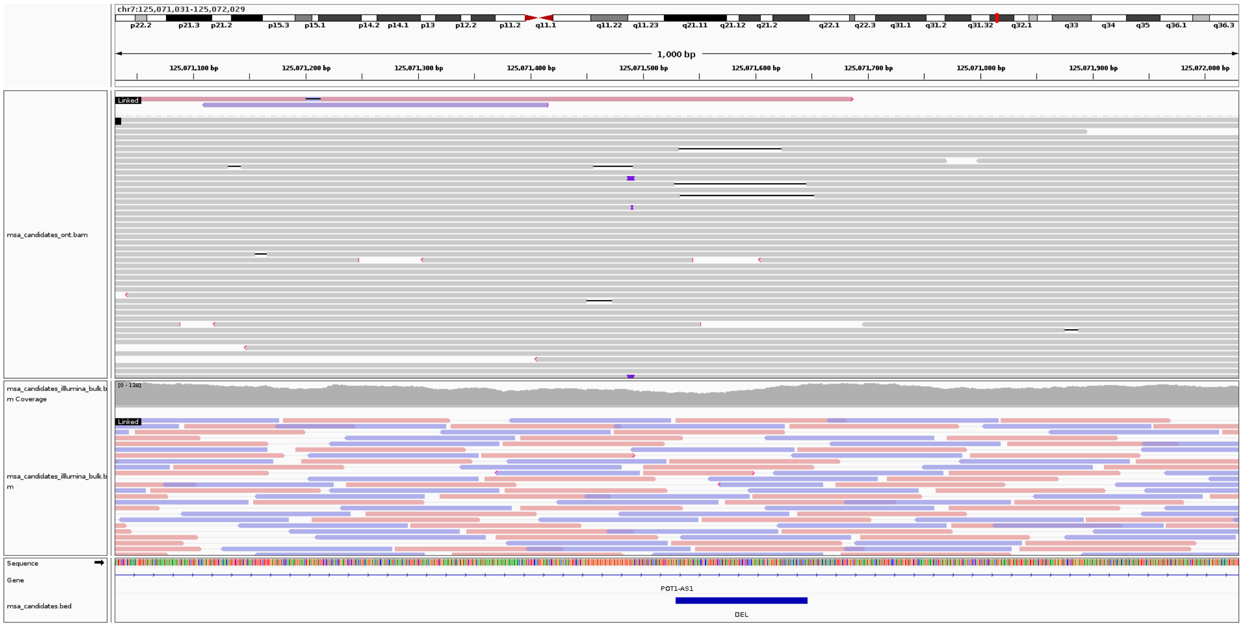
chr7:125,071,031-125,072,029
p22.2
p21.3
p21.2
p15.3
p15.1
p14.2
p14.1
p13
p12.2
p11.2
q11.1
q11.22
q11.23
q21.11
q21.12
q21.2
q22.1
q22.3
q31.1
q31.2
q31.32
q32.1
q33
q34
q35
q36.1
q36.3
1,000 bp
125,071,100 bp
125,071,200 bp
125,071,300 bp
125,071,400 bp
125,071,500 bp
125,071,600 bp
125,071,700 bp
125,071,800 bp
125,071,900 bp
125,072,000 bp
Linked
msa_candidates_ont.bam
msa_candidates_illumina_bulk.bam Coverage
[0 - 126]
msa_candidates_illumina_bulk.bam
Sequence
Gene
msa_candidates.bed
POT1-AS1
DEL

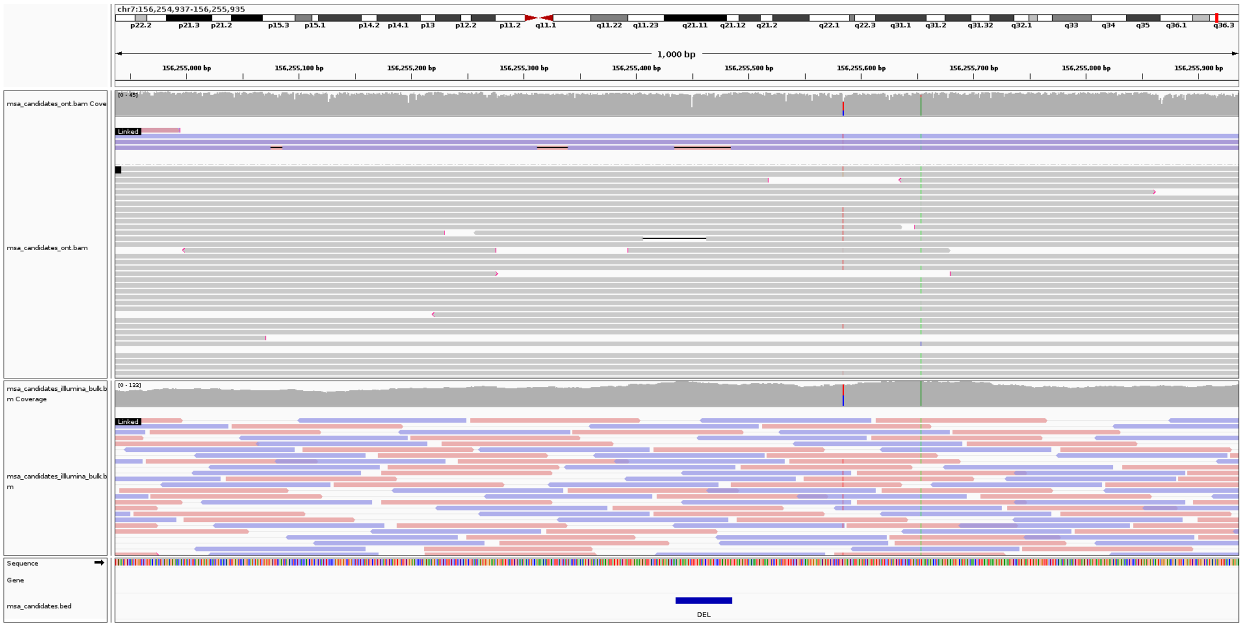
chr7:156,254,937-156,255,935
1,000 bp
msa_candidates_ont.bam Coverage
msa_candidates_ont.bam
msa_candidates_illumina_bulk.bam Coverage
msa_candidates_illumina_bulk.bam
Sequence
Gene
msa_candidates.bed
DEL

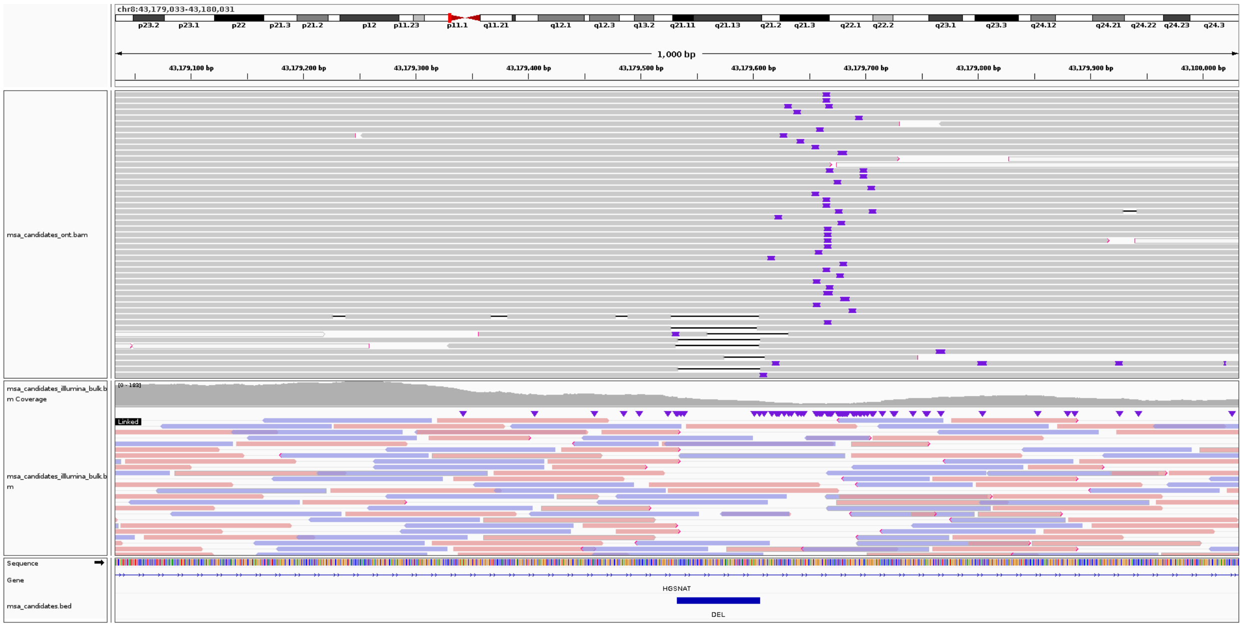
chr8:43,179,033-43,180,031
1,000 bp
msa_candidates_ont.bam
msa_candidates_illumina_bulk.bam Coverage
msa_candidates_illumina_bulk.bam
Sequence
Gene
msa_candidates.bed
HGSNAT
DEL

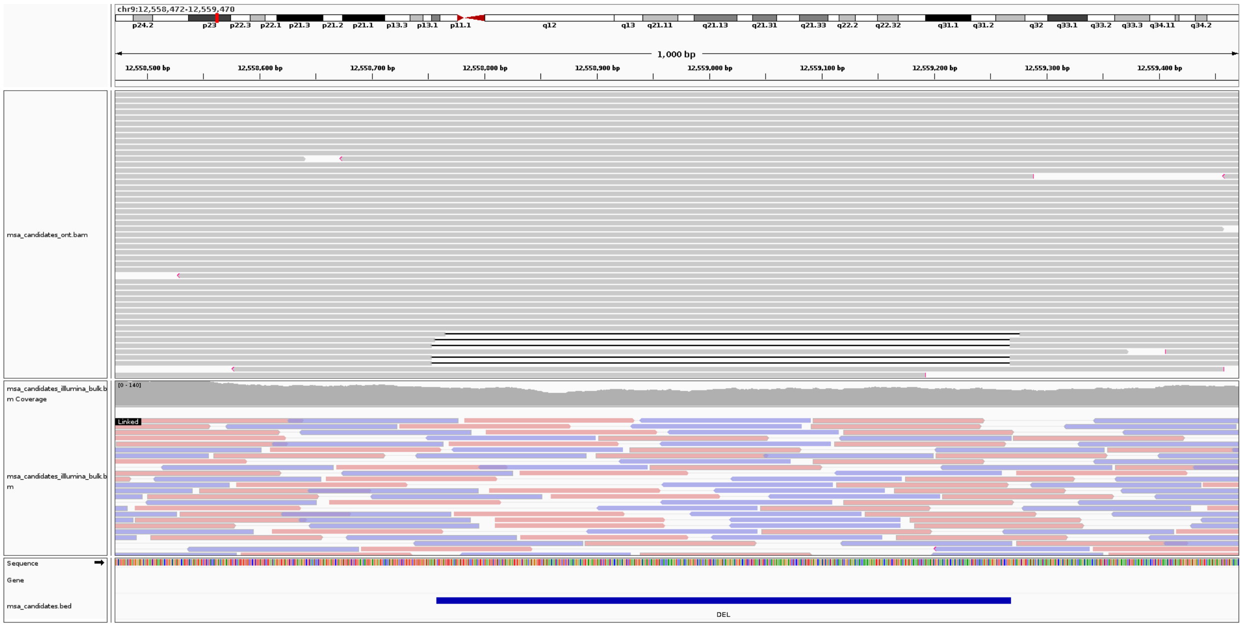
chr9:12,558,472-12,559,470
p24.2 p23 p22.3 p22.1 p21.3 p21.2 p21.1 p13.3 p13.1 p11.1 q12 q13 q21.11 q21.13 q21.31 q21.33 q22.2 q22.32 q31.1 q31.2 q32 q33.1 q33.2 q33.3 q34.11 q34.2
1,000 bp
12,558,500 bp
12,558,600 bp
12,558,700 bp
12,558,800 bp
12,558,900 bp
12,559,000 bp
12,559,100 bp
12,559,200 bp
12,559,300 bp
12,559,400 bp
msa_candidates_ont.bam
msa_candidates_illumina_bulk.bam Coverage
[0 - 140]
Linked
msa_candidates_illumina_bulk.bam
Sequence
Gene
msa_candidates.bed
DEL

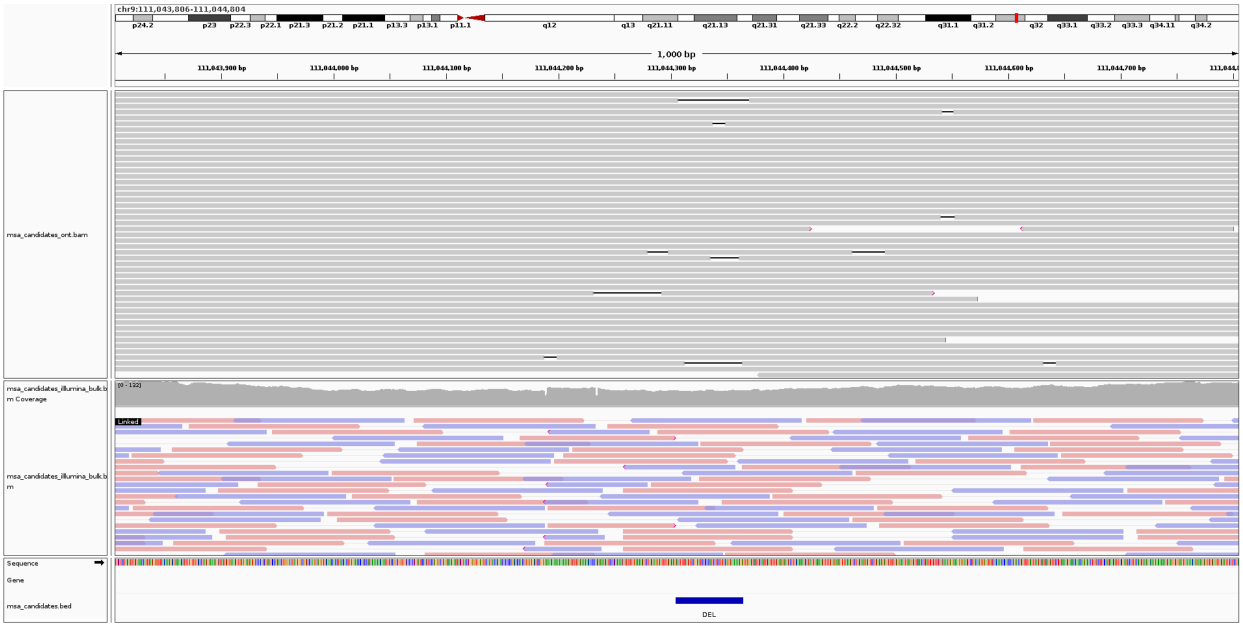
chr9:111,043,806-111,044,804
p24.2 p23 p22.3 p22.1 p21.3 p21.2 p21.1 p13.3 p13.1 p11.1 q12 q13 q21.11 q21.13 q21.31 q21.33 q22.2 q22.32 q31.1 q31.2 q32 q33.1 q33.2 q33.3 q34.11 q34.2
1,000 bp
111,043,900 bp
111,044,000 bp
111,044,100 bp
111,044,200 bp
111,044,300 bp
111,044,400 bp
111,044,500 bp
111,044,600 bp
111,044,700 bp
msa_candidates_ont.bam
msa_candidates_illumina_bulk.bam Coverage
[0 - 132]
Linked
msa_candidates_illumina_bulk.bam
Sequence
Gene
msa_candidates.bed
DEL

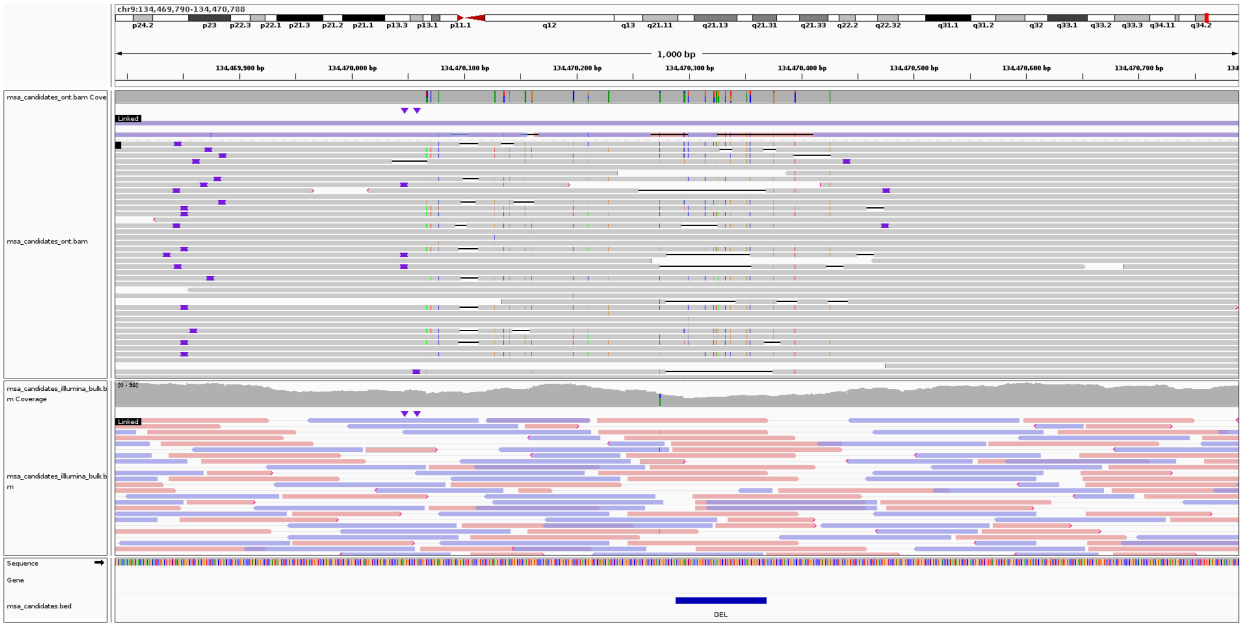
chr9:134,469,790-134,470,788
1,000 bp
134,469,900 bp
134,470,000 bp
134,470,100 bp
134,470,200 bp
134,470,300 bp
134,470,400 bp
134,470,500 bp
134,470,600 bp
134,470,700 bp
msa_candidates_ont.bam Coverage
msa_candidates_ont.bam
msa_candidates_illumina_bulk.bam Coverage
msa_candidates_illumina_bulk.bam
Linked
Sequence
Gene
msa_candidates.bed
DEL

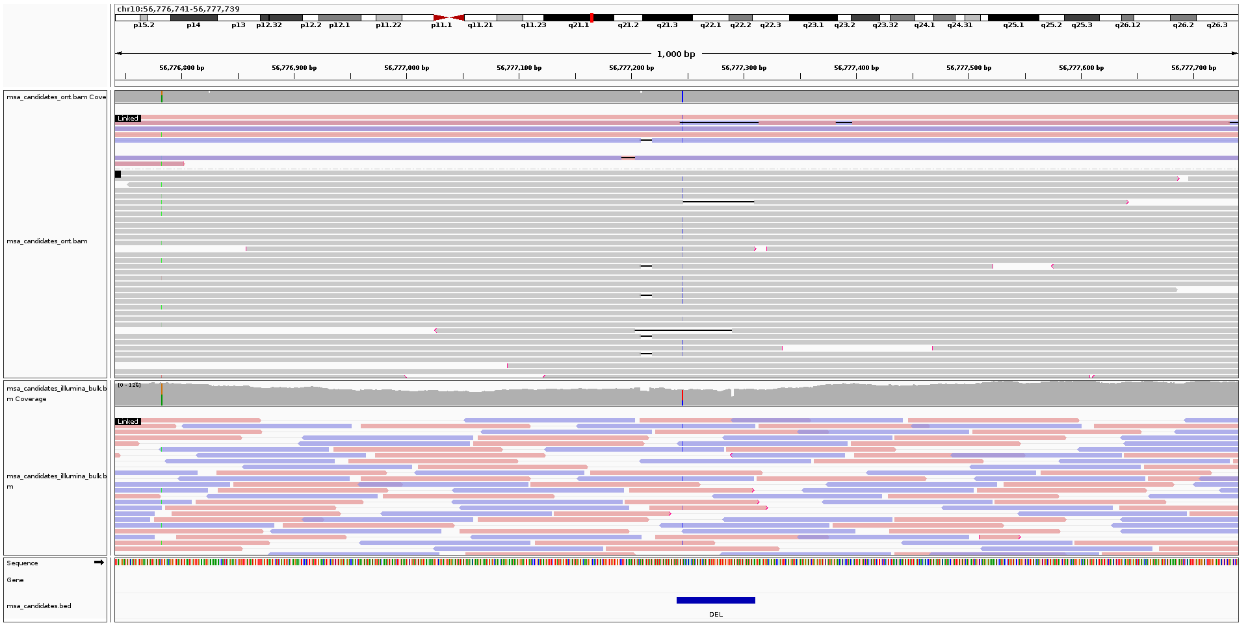
chr10:56,776,741-56,777,739
1,000 bp
56,776,800 bp
56,776,900 bp
56,777,000 bp
56,777,100 bp
56,777,200 bp
56,777,300 bp
56,777,400 bp
56,777,500 bp
56,777,600 bp
56,777,700 bp
msa_candidates_ont.bam Coverage
msa_candidates_ont.bam
msa_candidates_illumina_bulk.bam Coverage
msa_candidates_illumina_bulk.bam
Linked
Sequence
Gene
msa_candidates.bed
DEL

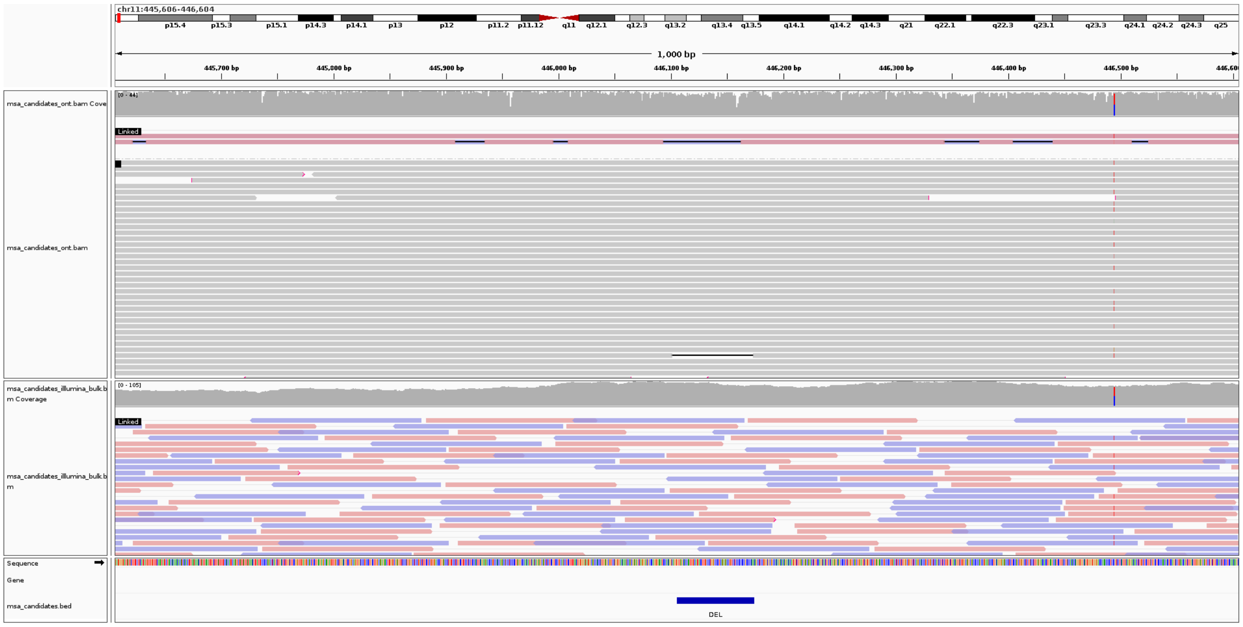
chr11:445,606-446,604
1,000 bp
445,700 bp
445,800 bp
445,900 bp
446,000 bp
446,100 bp
446,200 bp
446,300 bp
446,400 bp
446,500 bp
msa_candidates_ont.bam Coverage
msa_candidates_ont.bam
msa_candidates_illumina_bulk.bam Coverage
msa_candidates_illumina_bulk.bam
Linked
Sequence
Gene
msa_candidates.bed
DEL

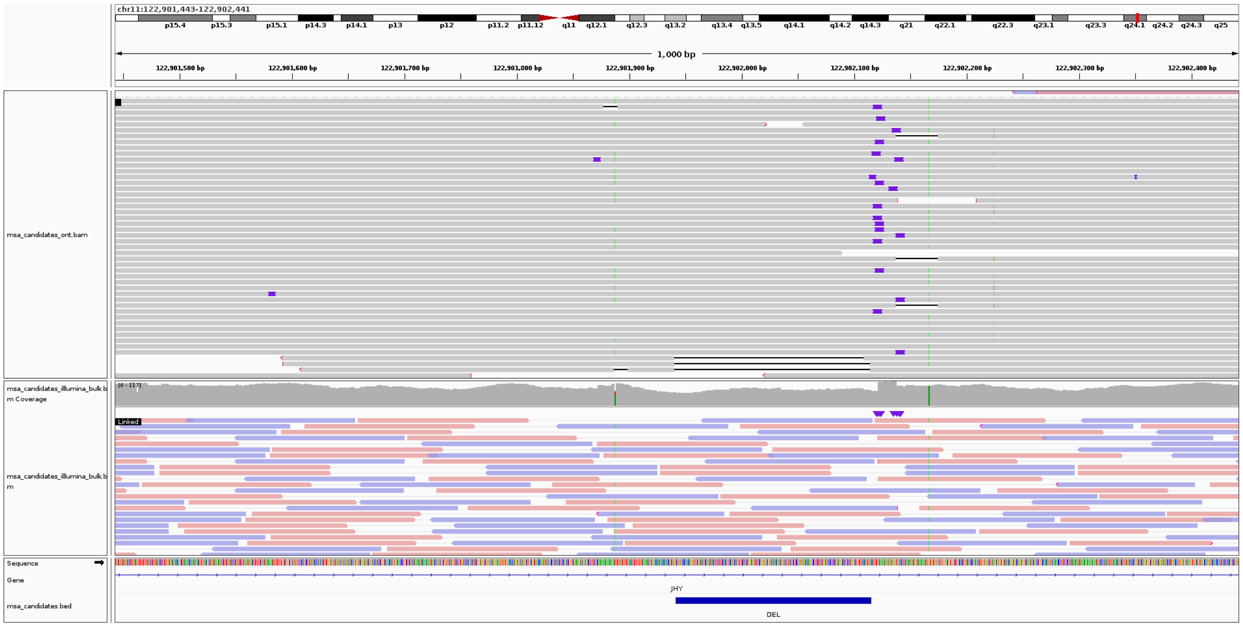
chr11:122,901,443-122,902,441
1,000 bp
122,901,500 bp
122,901,600 bp
122,901,700 bp
122,901,800 bp
122,901,900 bp
122,902,000 bp
122,902,100 bp
122,902,200 bp
122,902,300 bp
122,902,400 bp
msa_candidates_ont.bam
msa_candidates_illumina_bulk.bam Coverage
msa_candidates_illumina_bulk.bam
Linked
Sequence
Gene
JHY
msa_candidates.bed
DEL

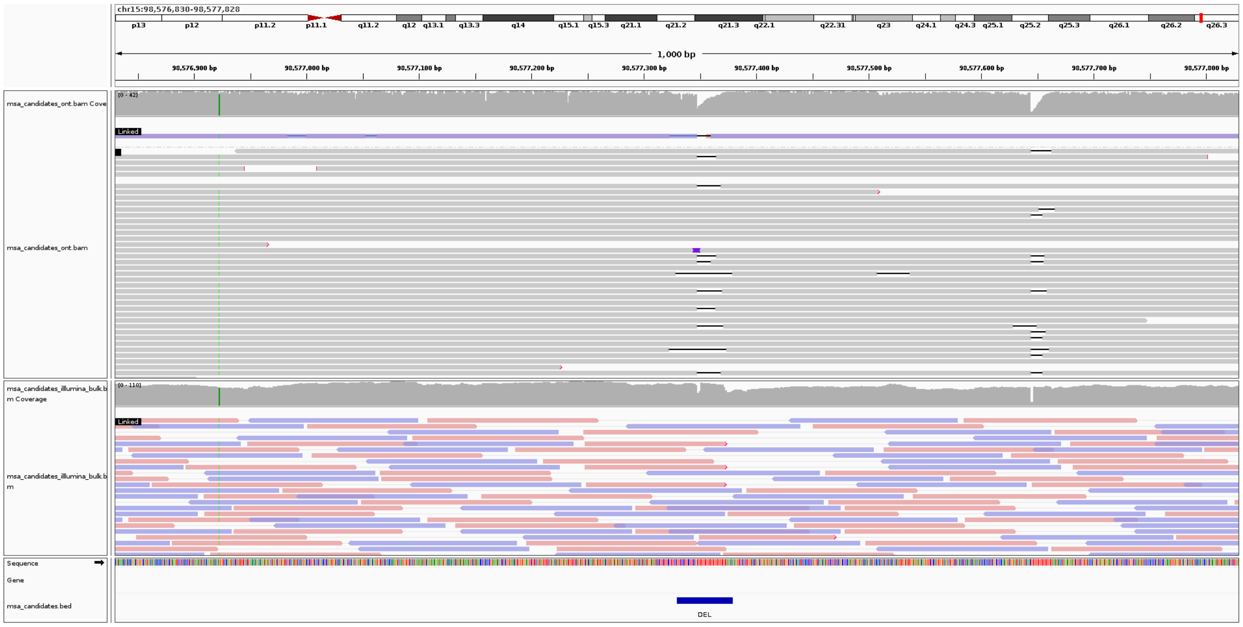
chr15:98,576,830-98,577,828
p13 p12 p11.2 p11.1 q11.2 q12 q13.1 q13.3 q14 q15.1 q15.3 q21.1 q21.2 q21.3 q22.1 q22.31 q23 q24.1 q24.3 q25.1 q25.2 q25.3 q26.1 q26.2 q26.3
1,000 bp
98,576,900 bp 98,577,000 bp 98,577,100 bp 98,577,200 bp 98,577,300 bp 98,577,400 bp 98,577,500 bp 98,577,600 bp 98,577,700 bp 98,577,800 bp
msa_candidates_ont.bam Cove
[0 - 42]
Linked
msa_candidates_ont.bam
msa_candidates_illumina_bulk.bam Coverage
[0 - 110]
Linked
msa_candidates_illumina_bulk.bam
Sequence
Gene
msa_candidates.bed
DEL

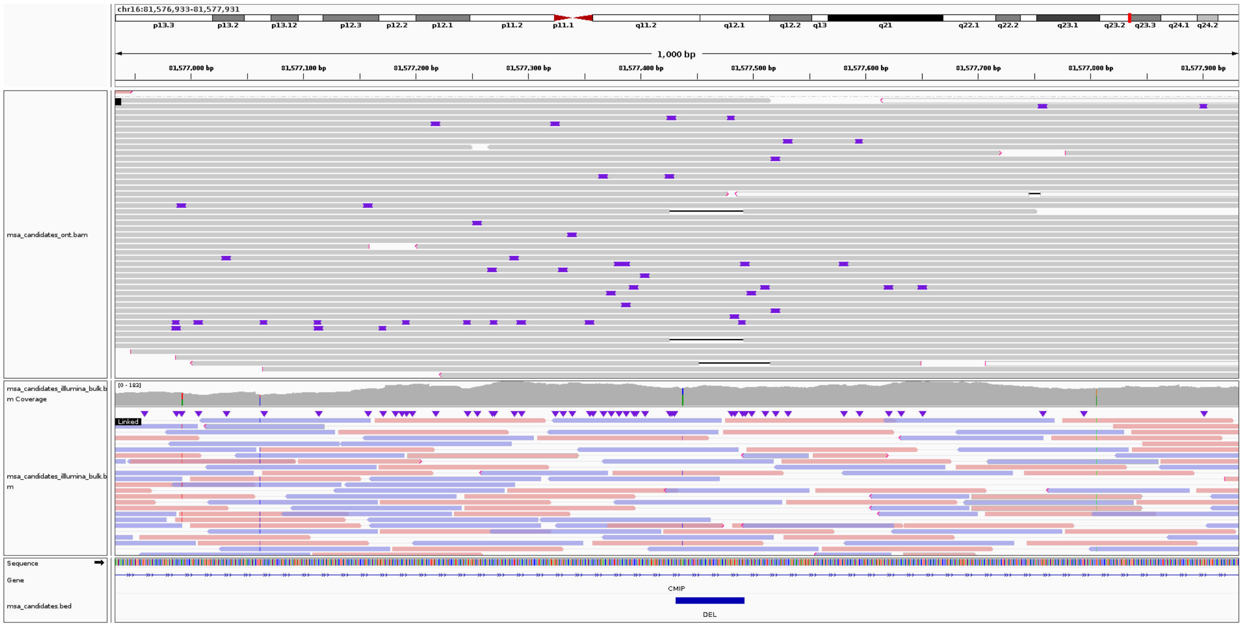
chr16:81,576,933-81,577,931
p13.3 p13.2 p13.12 p12.3 p12.2 p12.1 p11.2 p11.1 q11.2 q12.1 q12.2 q13 q21 q22.1 q22.2 q23.1 q23.2 q23.3 q24.1 q24.2
1,000 bp
81,577,000 bp 81,577,100 bp 81,577,200 bp 81,577,300 bp 81,577,400 bp 81,577,500 bp 81,577,600 bp 81,577,700 bp 81,577,800 bp 81,577,900 bp
msa_candidates_ont.bam
msa_candidates_illumina_bulk.bam Coverage
[0 - 182]
Linked
msa_candidates_illumina_bulk.bam
Sequence
Gene
msa_candidates.bed
CMIP
DEL

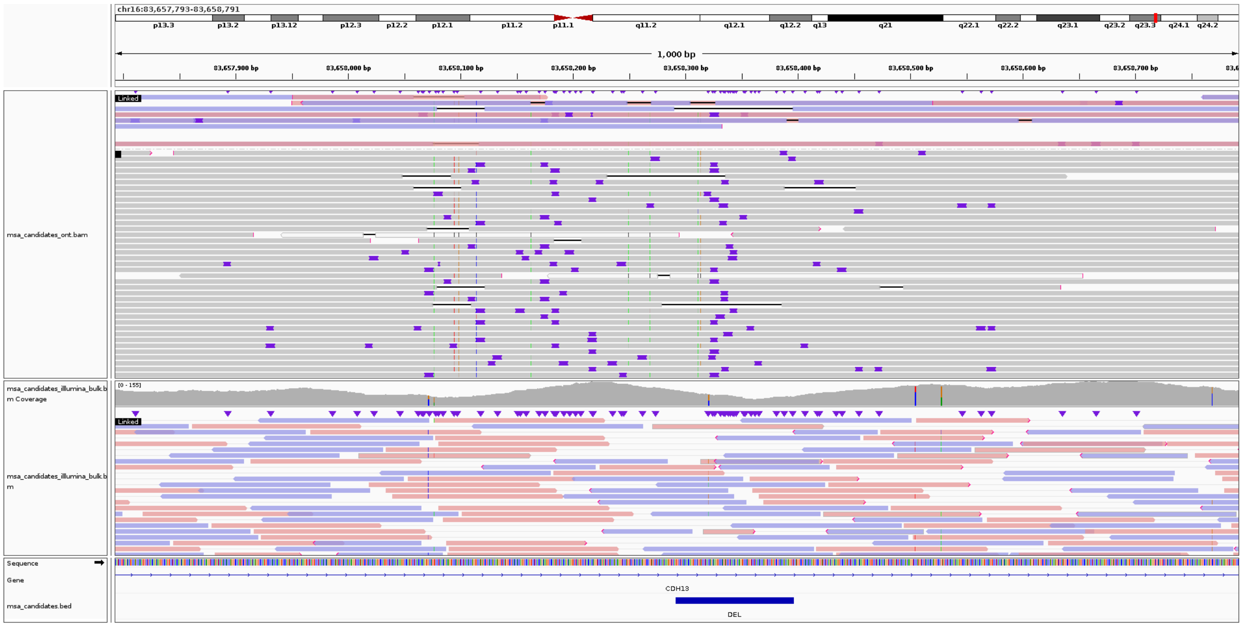
chr16:83,657,793-83,658,791
1,000 bp
83,657,900 bp
83,658,000 bp
83,658,100 bp
83,658,200 bp
83,658,300 bp
83,658,400 bp
83,658,500 bp
83,658,600 bp
83,658,700 bp
msa_candidates_ont.bam
msa_candidates_illumina_bulk.bam Coverage
[0 - 155]
msa_candidates_illumina_bulk.bam
Sequence
Gene
msa_candidates.bed
CDH13
DEL

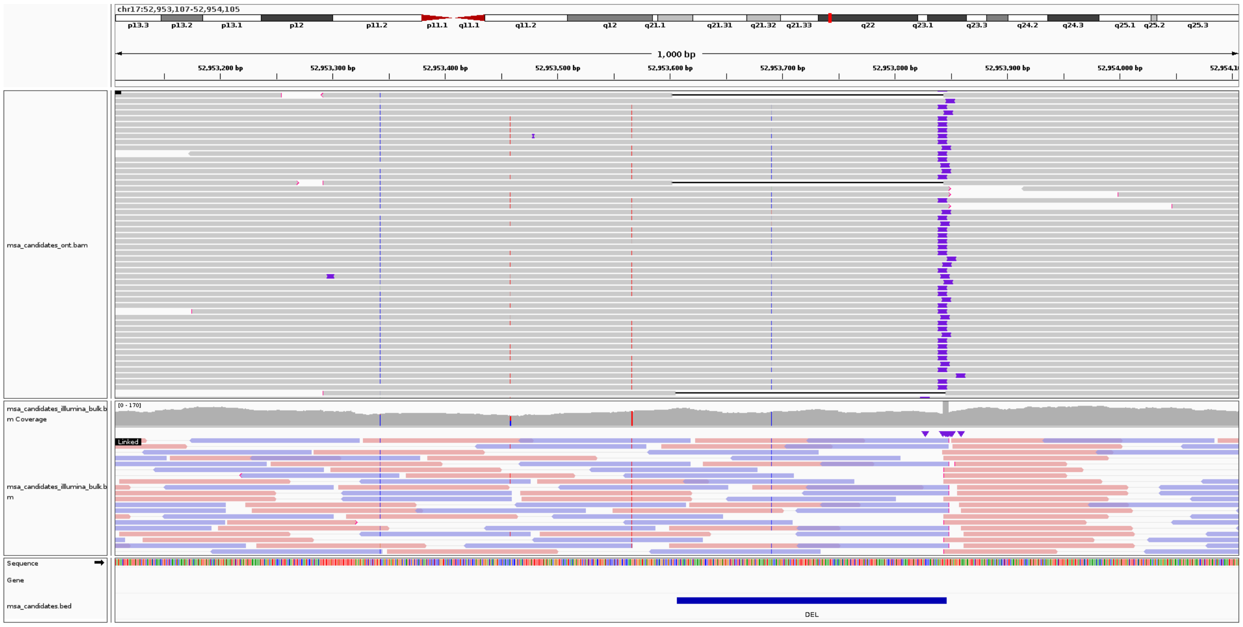
chr17:52,953,107-52,954,105
1,000 bp
52,953,200 bp
52,953,300 bp
52,953,400 bp
52,953,500 bp
52,953,600 bp
52,953,700 bp
52,953,800 bp
52,953,900 bp
52,954,000 bp
msa_candidates_ont.bam
msa_candidates_illumina_bulk.bam Coverage
[0 - 170]
msa_candidates_illumina_bulk.bam
Sequence
Gene
msa_candidates.bed
DEL

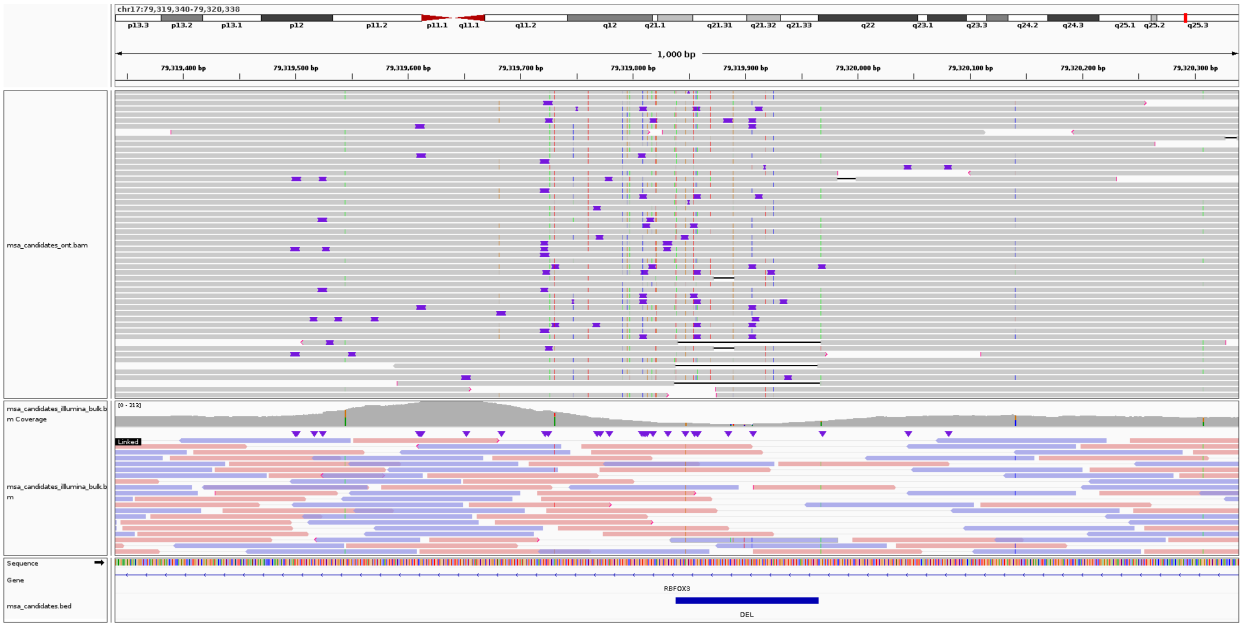
chr17:79,319,340-79,320,338
1,000 bp
79,319,400 bp
79,319,500 bp
79,319,600 bp
79,319,700 bp
79,319,800 bp
79,319,900 bp
79,320,000 bp
79,320,100 bp
79,320,200 bp
79,320,300 bp
msa_candidates_ont.bam
msa_candidates_illumina_bulk.bam Coverage
[0 - 213]
Linked
msa_candidates_illumina_bulk.bam
Sequence
Gene
msa_candidates.bed
RBFOX3
DEL

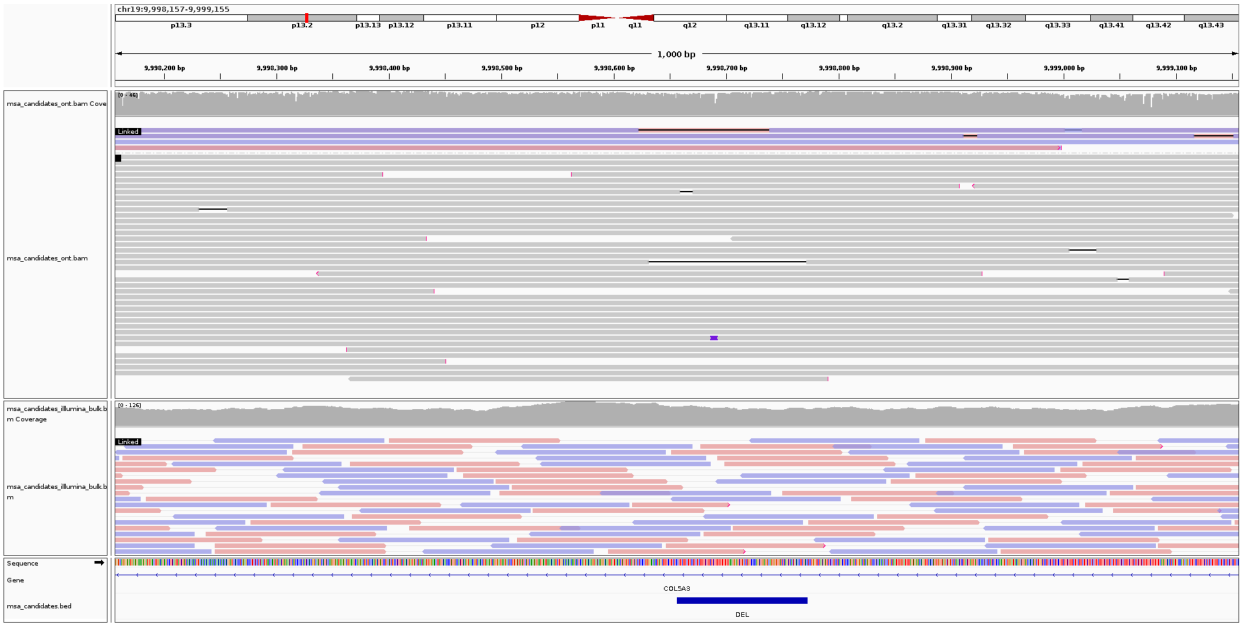
chr19:9,998,157-9,999,155
1,000 bp
9,998,200 bp
9,998,300 bp
9,998,400 bp
9,998,500 bp
9,998,600 bp
9,998,700 bp
9,998,800 bp
9,998,900 bp
9,999,000 bp
9,999,100 bp
msa_candidates_ont.bam Cove
[0 - 46]
Linked
msa_candidates_ont.bam
msa_candidates_illumina_bulk.bam Coverage
[0 - 126]
Linked
msa_candidates_illumina_bulk.bam
Sequence
Gene
msa_candidates.bed
COL5A3
DEL

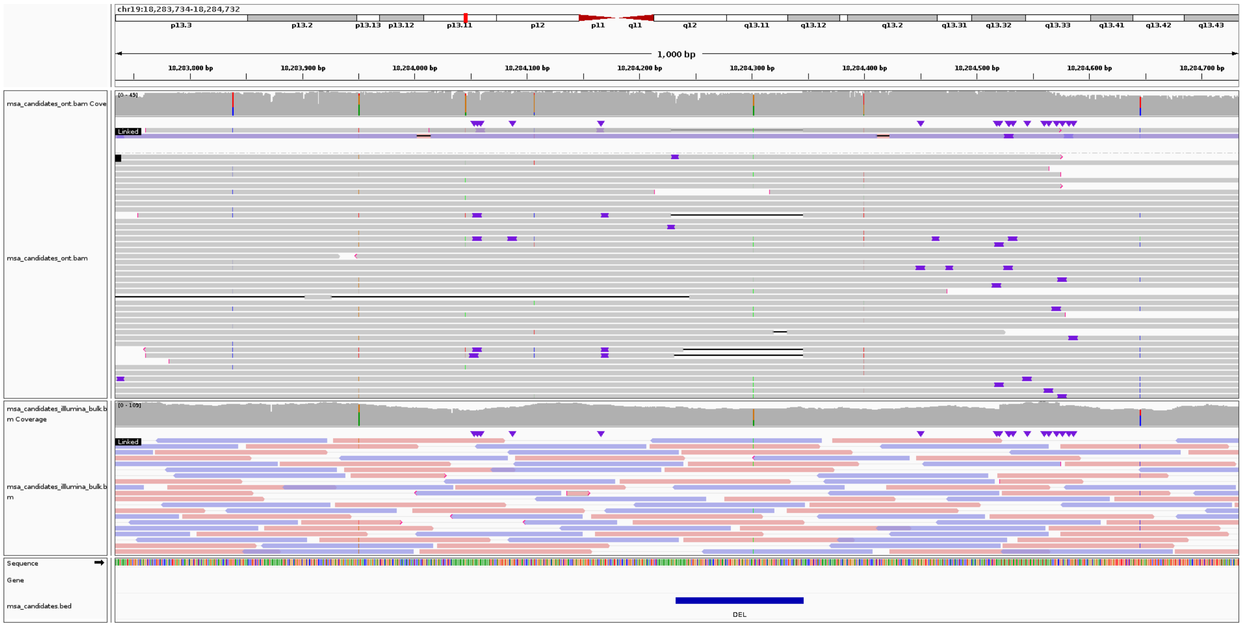
chr19:18,283,734-18,284,732
1,000 bp
msa_candidates_ont.bam Coverage
msa_candidates_ont.bam
msa_candidates_illumina_bulk.bam Coverage
msa_candidates_illumina_bulk.bam
Linked
Sequence
Gene
msa_candidates.bed
DEL

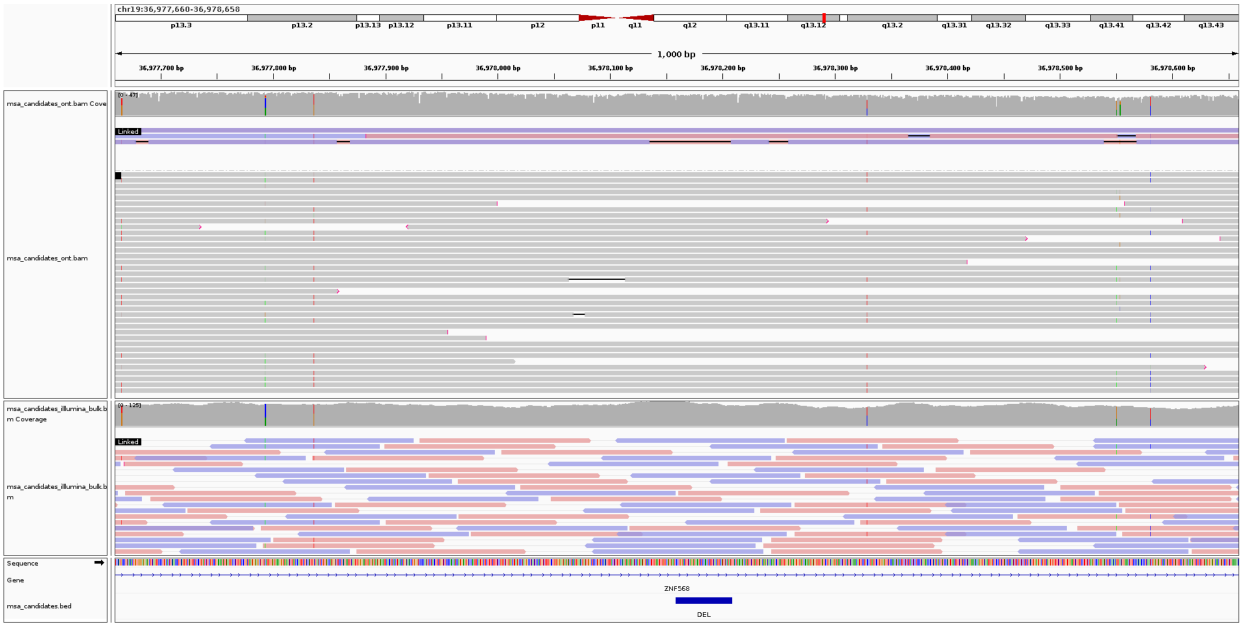
chr19:36,977,660-36,978,658
1,000 bp
msa_candidates_ont.bam Coverage
msa_candidates_ont.bam
msa_candidates_illumina_bulk.bam Coverage
msa_candidates_illumina_bulk.bam
Linked
Sequence
Gene
msa_candidates.bed
ZNF568
DEL

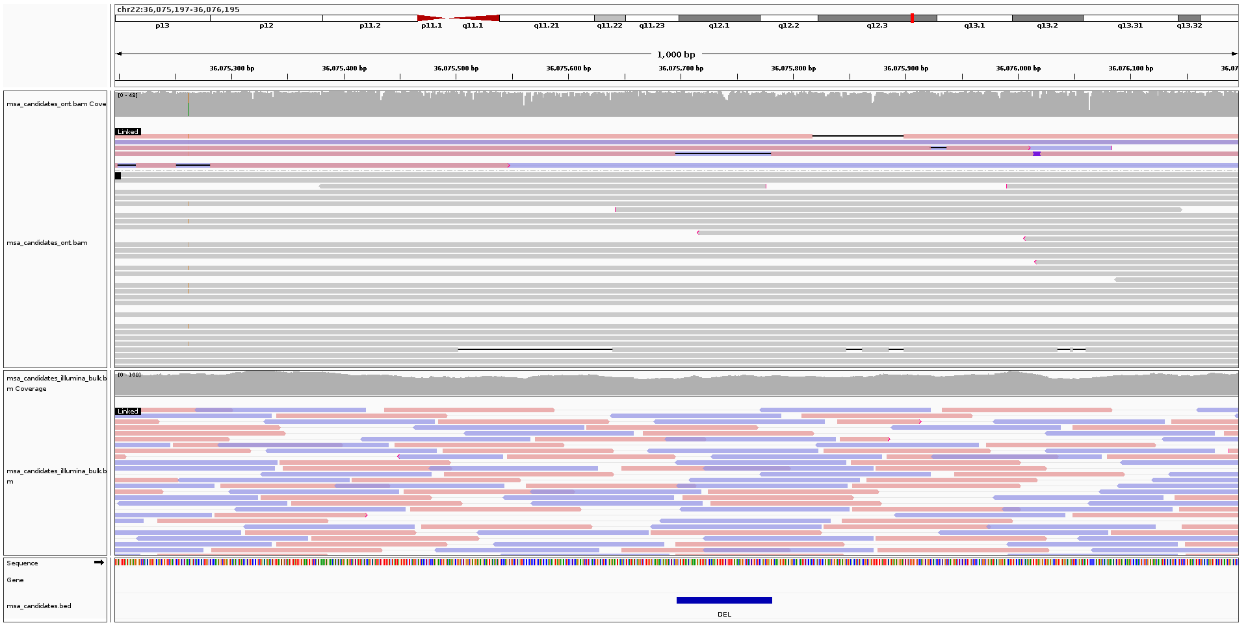

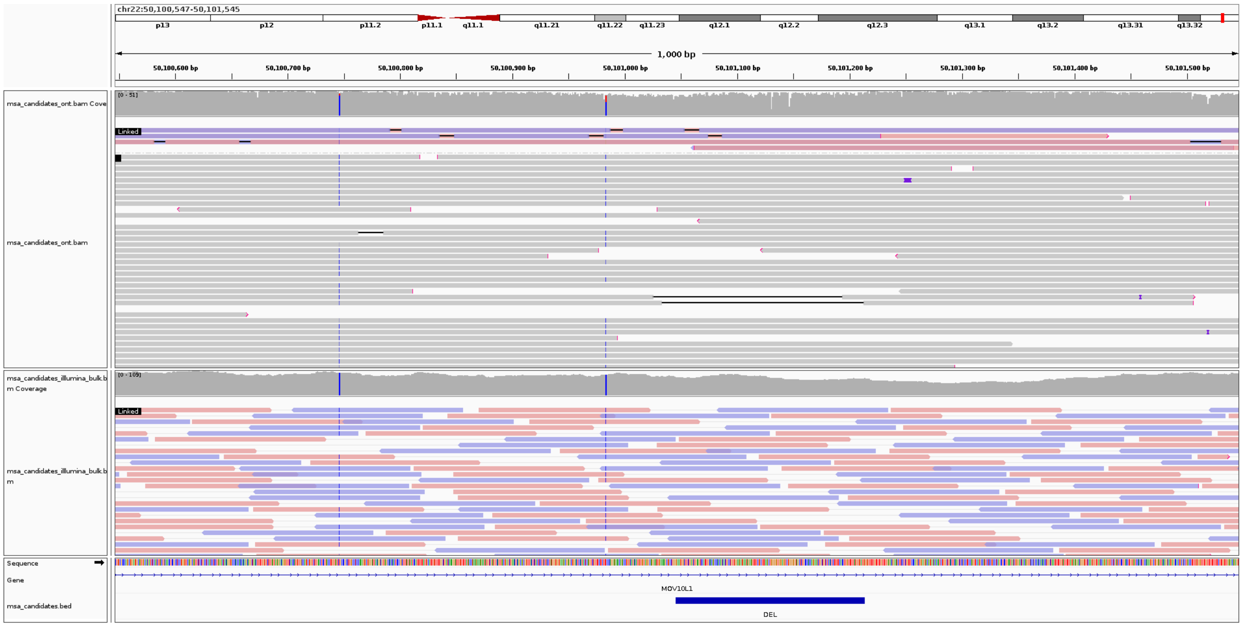

Supplementary figure 8 A-AH: IGV screenshots of 34 mosaic SVs from the MSA sample. See **Supplementary table 14** for more details.

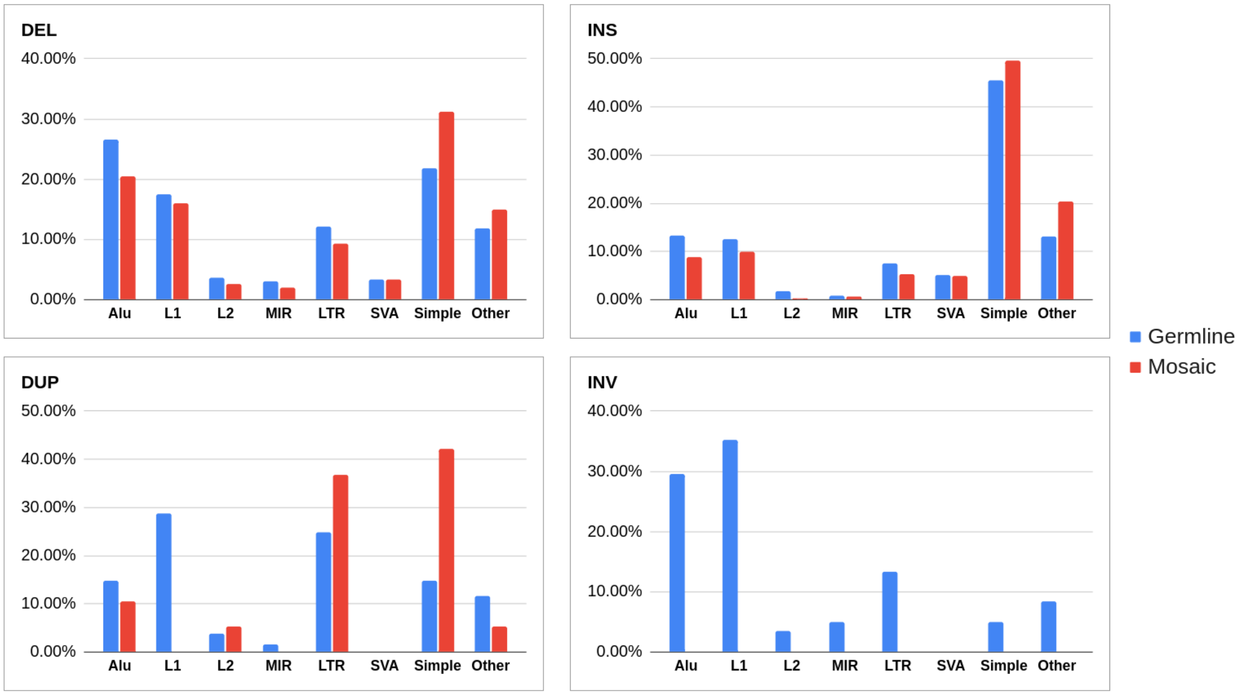

Supplementary Figure 9

Fraction of germline and non-germline/ mosaic SVs associated to different elements of repeat families in the cingulate cortex sample (ONT), split by SV type.

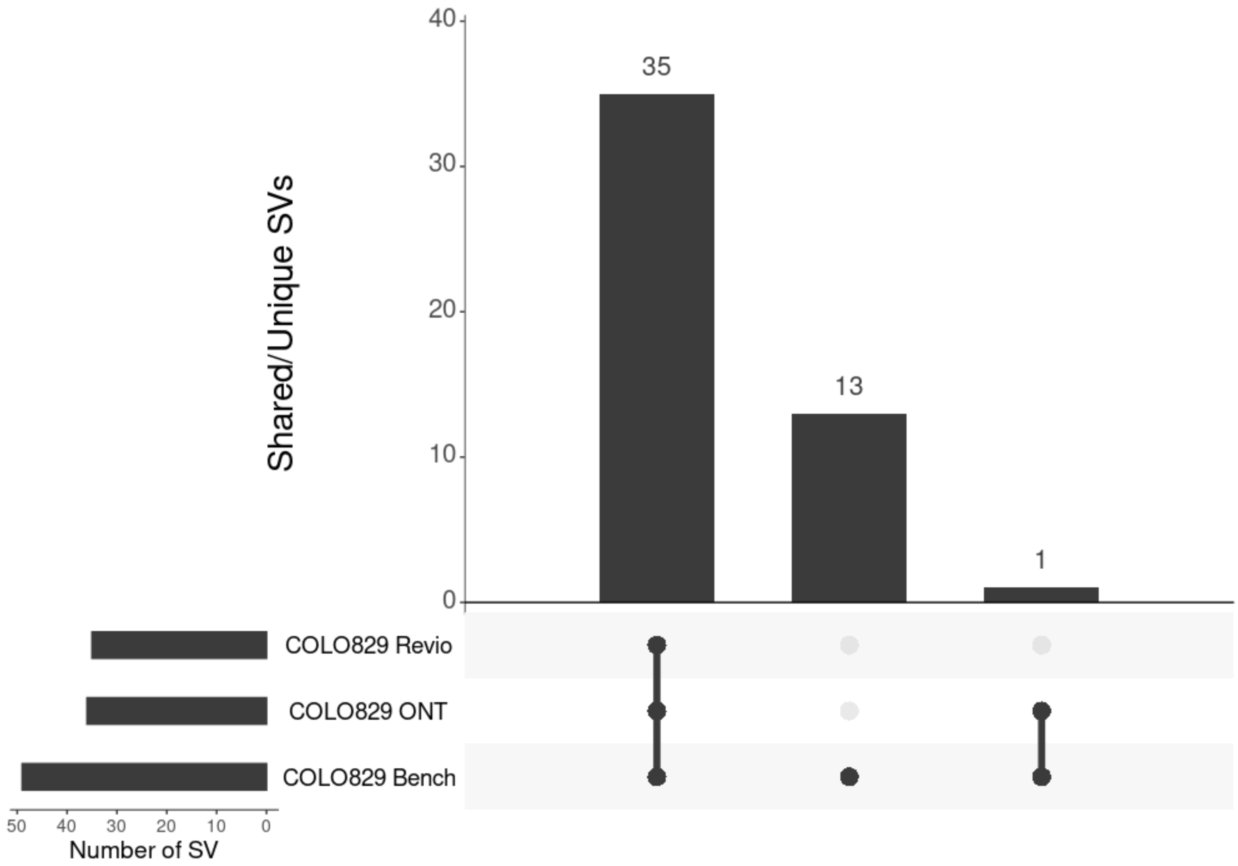

Supplementary Figure 10

Germline and mosaic SV called by Sniffles2 in the COLO829 cell line compared to a benchmark containing 49 SV (no BND). In total 36 SVs from the benchmark are detected and 13 are missed. From those 13: four had no reads for the alternative allele (0% AF), three were identified by Sniffles as mosaic (8.2-21% AF), four had heterozygous genotype (31-41% AF) and two had homozygous alternative genotype (81-100% AF)

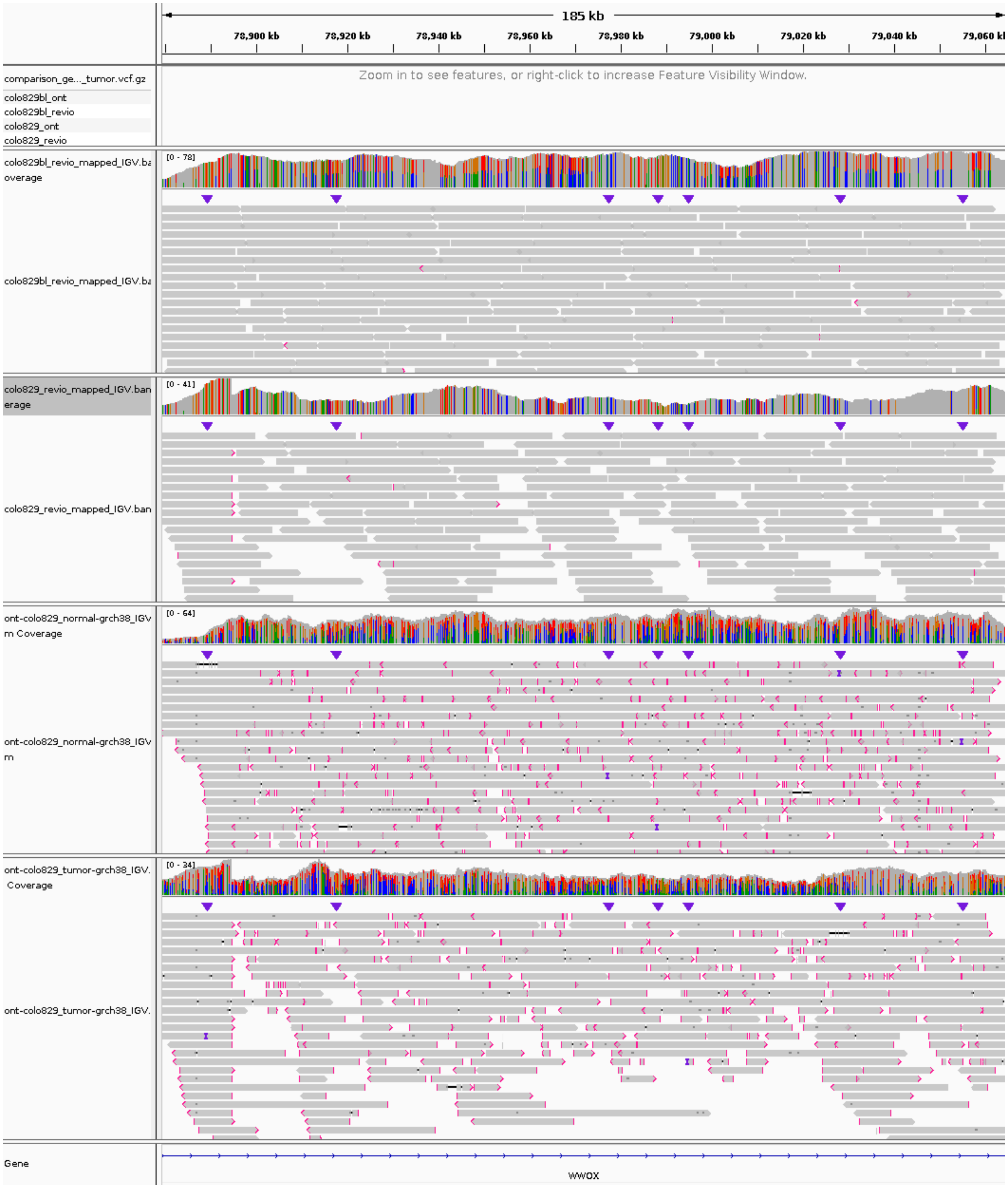

Supplementary Figure 11

Cancer specific germline SV call identified by sniffles2. We call germline SV for both the normal sample and cancer sample. After the merge we selected SV that were only detected in the cancer sample. Here is shown an example of an heterozygous deletion in an intron of the WWOX gene.

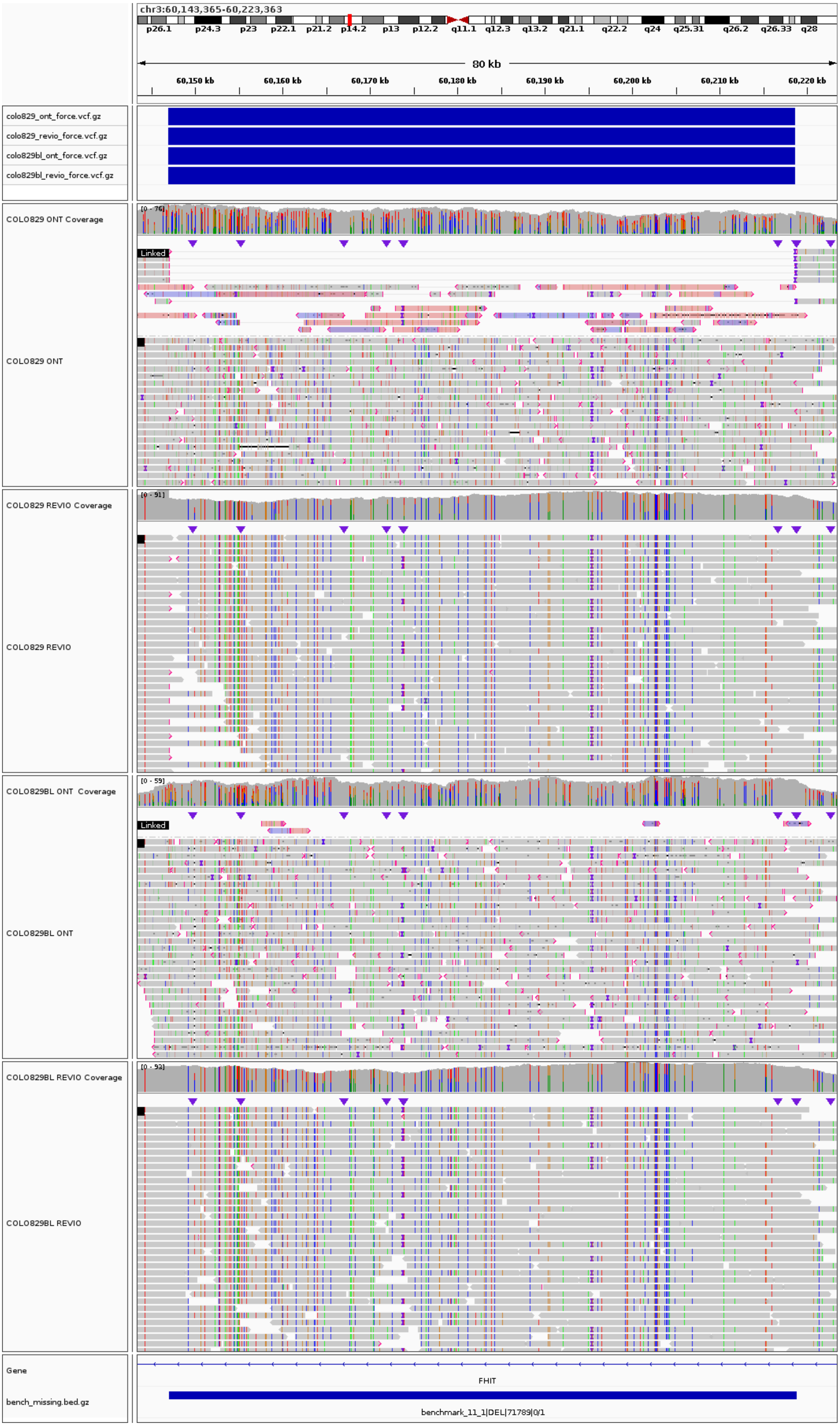

chr3:60,143,365-60,223,363
p26.1 p24.3 p23 p22.1 p21.2 p14.2 p13 p12.2 q11.1 q12.3 q13.2 q21.1 q22.2 q24 q25.31 q26.2 q26.33 q28
80 kb
60,150 kb 60,160 kb 60,170 kb 60,180 kb 60,190 kb 60,200 kb 60,210 kb 60,220 kb
colo829_ont_force.vcf.gz
colo829_revio_force.vcf.gz
colo829bl_ont_force.vcf.gz
colo829bl_revio_force.vcf.gz
COLO829 ONT Coverage
[0 - 76]
Linked
COLO829 ONT
COLO829 REVIO Coverage
[0 - 91]
COLO829 REVIO
COLO829BL ONT Coverage
[0 - 59]
Linked
COLO829BL ONT
COLO829BL REVIO Coverage
[0 - 53]
COLO829BL REVIO
Gene
FHIT
bench_missing.bed.gz
benchmark_11_1|DEL|71789|0/1

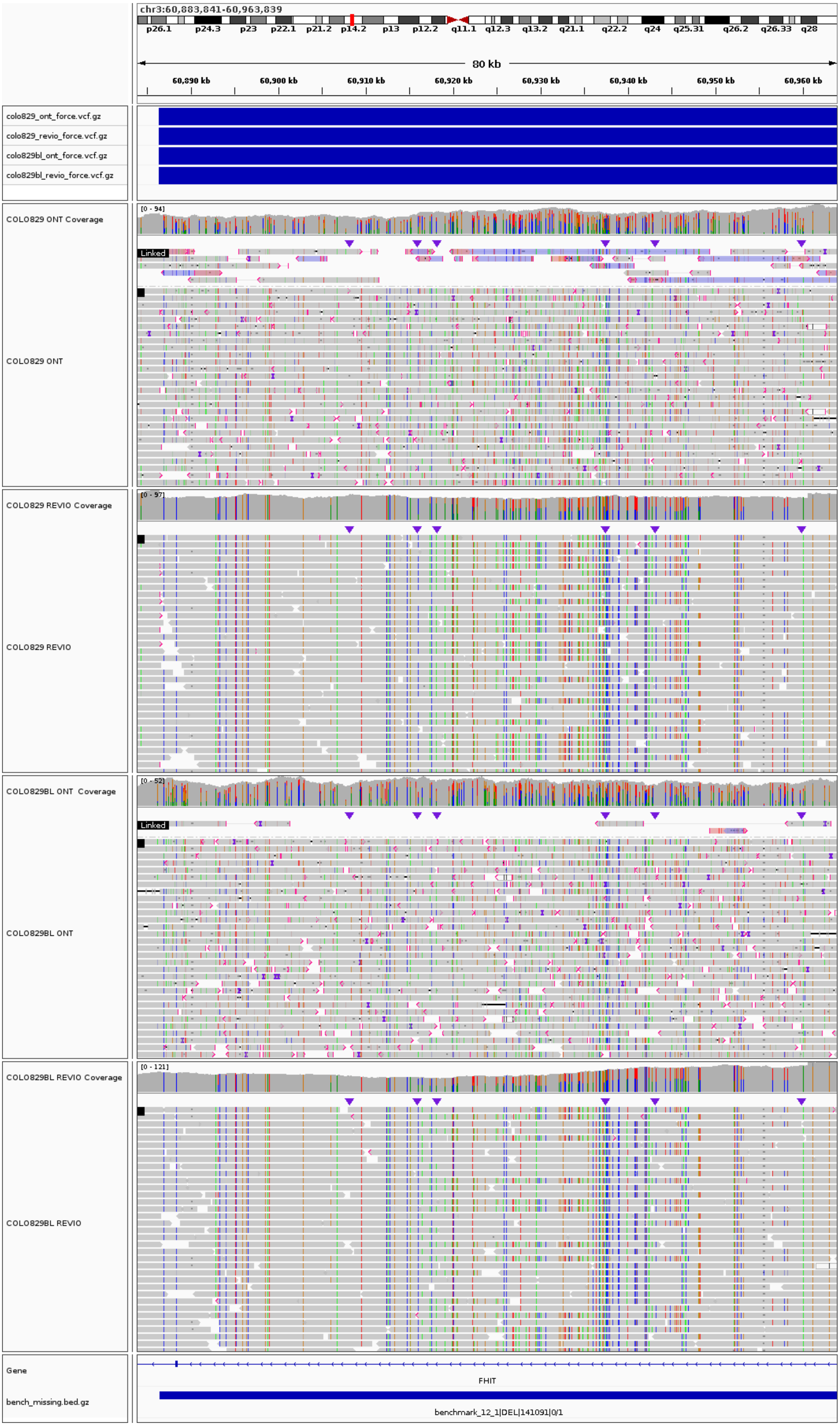
chr3:60,883,841-60,963,839
p26.1 p24.3 p23 p22.1 p21.2 p14.2 p13 p12.2 q11.1 q12.3 q13.2 q21.1 q22.2 q24 q25.31 q26.2 q26.33 q28
80 kb
60,890 kb 60,900 kb 60,910 kb 60,920 kb 60,930 kb 60,940 kb 60,950 kb 60,960 kb
colo829_ont_force.vcf.gz
colo829_revio_force.vcf.gz
colo829bl_ont_force.vcf.gz
colo829bl_revio_force.vcf.gz
COLO829 ONT Coverage
[0 - 94]
Linked
COLO829 ONT
COLO829 REVIO Coverage
[0 - 97]
COLO829 REVIO
COLO829BL ONT Coverage
[0 - 52]
Linked
COLO829BL ONT
COLO829BL REVIO Coverage
[0 - 121]
COLO829BL REVIO
Gene
FHIT
bench_missing.bed.gz
benchmark_12_1|DEL|141091|0/1

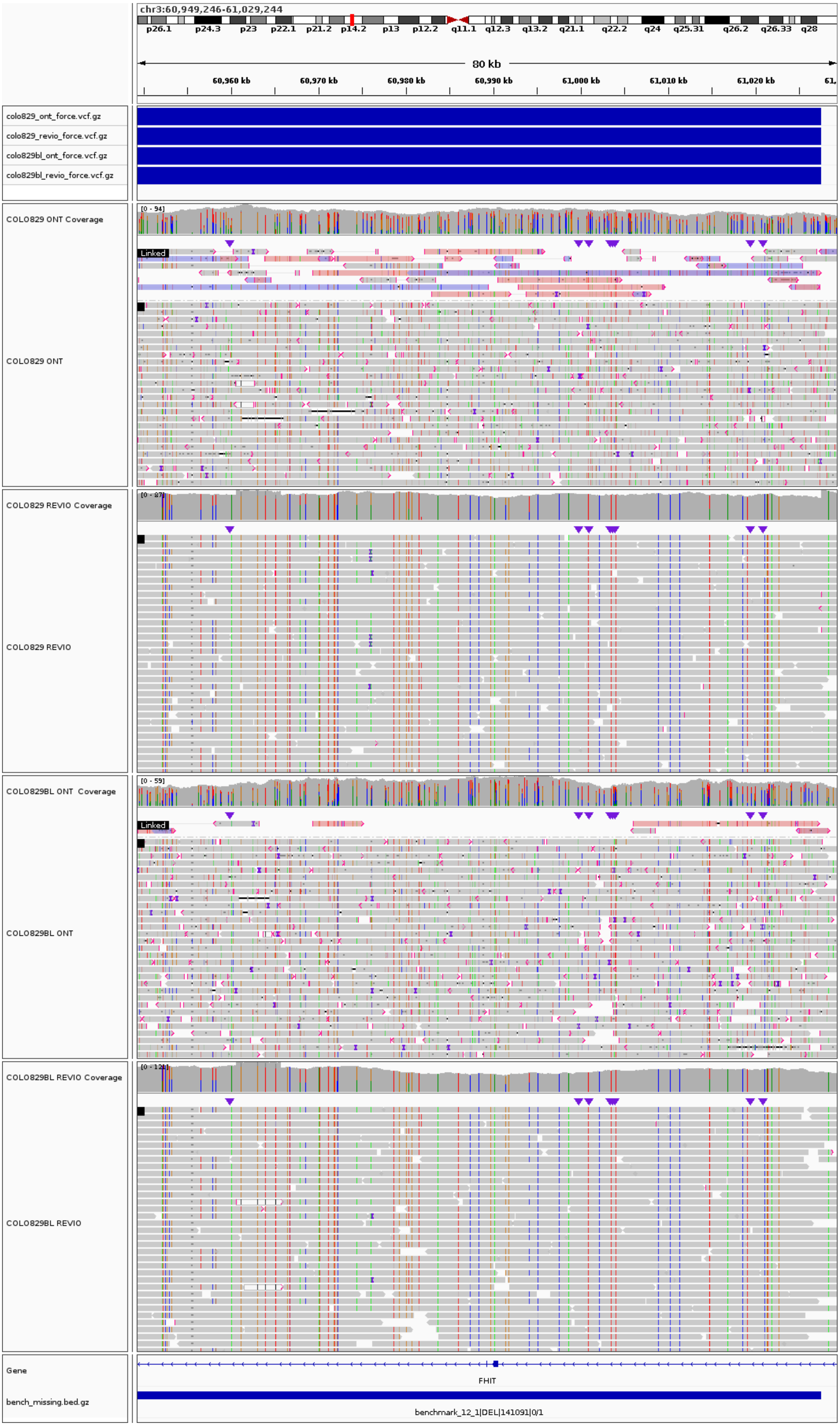

chr3:60,949,246-61,029,244
p26.1 p24.3 p23 p22.1 p21.2 p14.2 p13 p12.2 q11.1 q12.3 q13.2 q21.1 q22.2 q24 q25.31 q26.2 q26.33 q28
80 kb
60,960 kb 60,970 kb 60,980 kb 60,990 kb 61,000 kb 61,010 kb 61,020 kb
colo829_ont_force.vcf.gz
colo829_revio_force.vcf.gz
colo829bl_ont_force.vcf.gz
colo829bl_revio_force.vcf.gz
COLO829 ONT Coverage
[0 - 94]
Linked
COLO829 ONT
COLO829 REVIO Coverage
[0 - 87]
COLO829 REVIO
COLO829BL ONT Coverage
[0 - 59]
Linked
COLO829BL ONT
COLO829BL REVIO Coverage
[0 - 121]
COLO829BL REVIO
Gene
FHIT
bench_missing.bed.gz
benchmark_12_1|DEL|141091|0/1

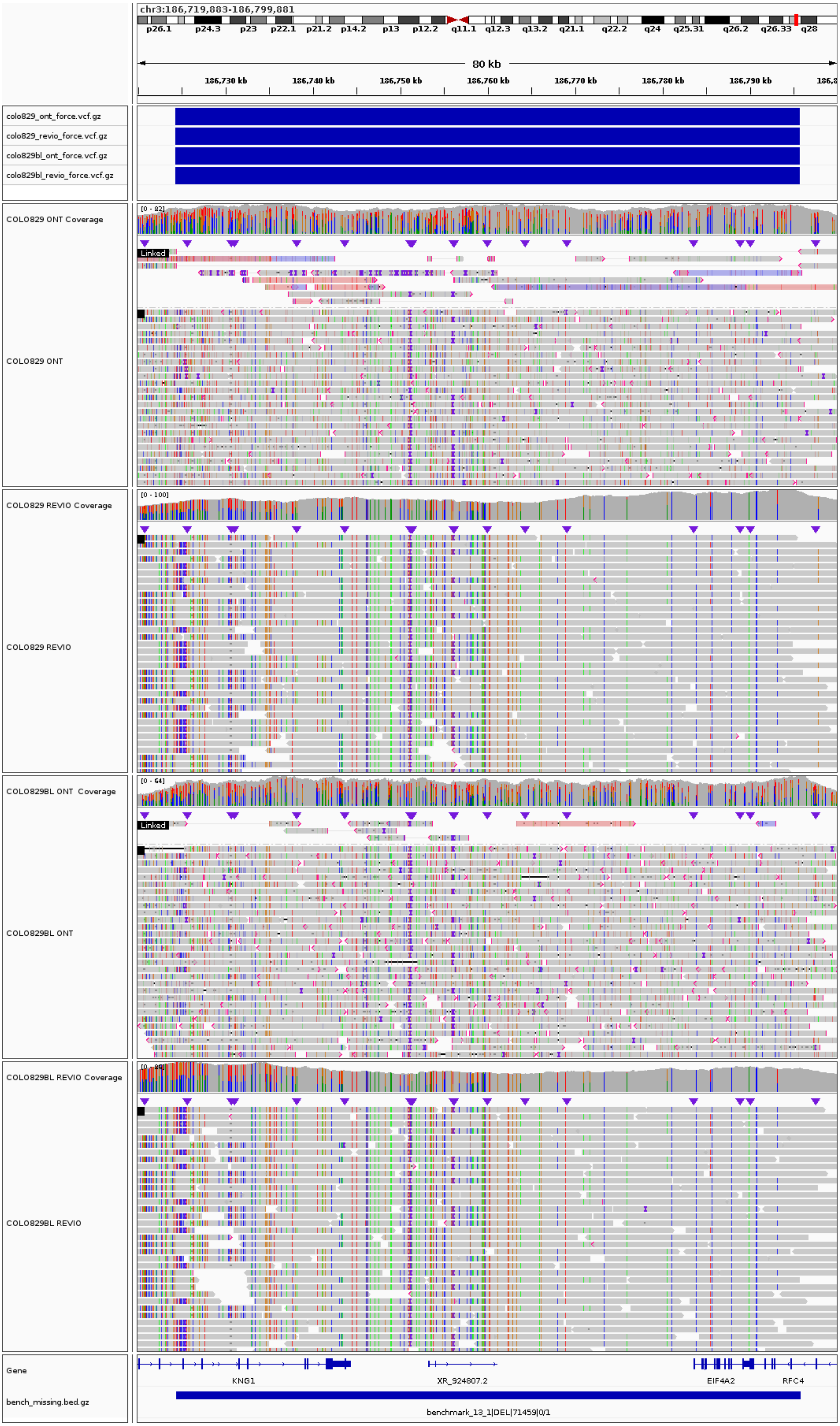

chr3:186,719,883-186,799,881
p26.1 p24.3 p23 p22.1 p21.2 p14.2 p13 p12.2 q11.1 q12.3 q13.2 q21.1 q22.2 q24 q25.31 q26.2 q26.33 q28
80 kb
186,730 kb 186,740 kb 186,750 kb 186,760 kb 186,770 kb 186,780 kb 186,790 kb
colo829_ont_force.vcf.gz
colo829_revio_force.vcf.gz
colo829bl_ont_force.vcf.gz
colo829bl_revio_force.vcf.gz
COLO829 ONT Coverage
[0 - 82]
Linked
COLO829 ONT
COLO829 REVIO Coverage
[0 - 100]
COLO829 REVIO
COLO829BL ONT Coverage
[0 - 64]
Linked
COLO829BL ONT
COLO829BL REVIO Coverage
COLO829BL REVIO
Gene
KNG1
XR_924807.2
EIF4A2
RFC4
bench_missing.bed.gz
benchmark_13_1|DEL|71459|0/1

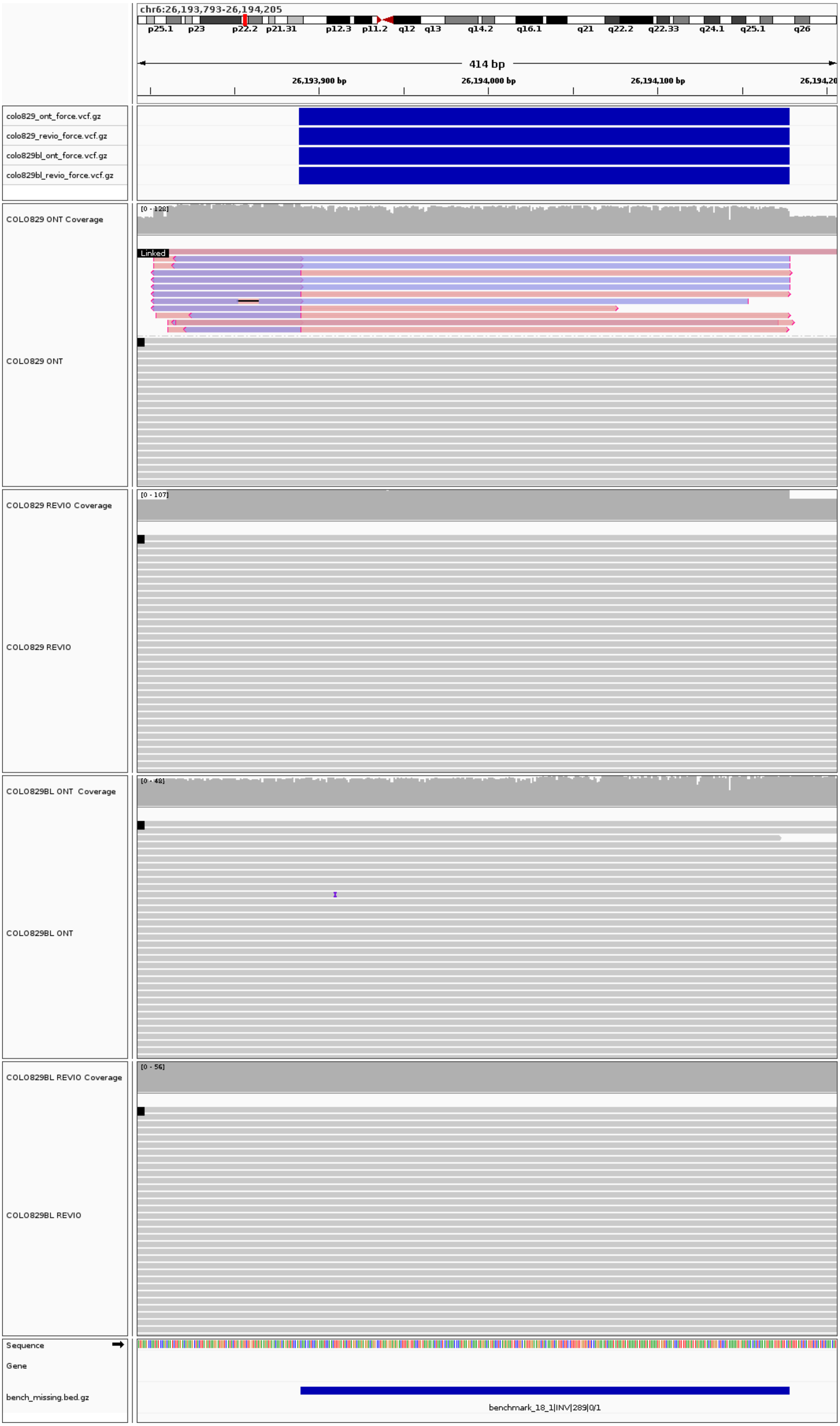
chr6:26,193,793-26,194,205
p25.1 p23 p22.2 p21.31 p12.3 p11.2 q12 q13 q14.2 q16.1 q21 q22.2 q22.33 q24.1 q25.1 q26
414 bp
26,193,900 bp
26,194,000 bp
26,194,100 bp
colo829_ont_force.vcf.gz
colo829_revio_force.vcf.gz
colo829bl_ont_force.vcf.gz
colo829bl_revio_force.vcf.gz
COLO829 ONT Coverage
[0 - 128]
Linked
COLO829 ONT
COLO829 REVIO Coverage
[0 - 107]
COLO829 REVIO
COLO829BL ONT Coverage
[0 - 48]
COLO829BL ONT
COLO829BL REVIO Coverage
[0 - 56]
COLO829BL REVIO
Sequence
Gene
bench_missing.bed.gz
benchmark_18_1|INV|289|0/1

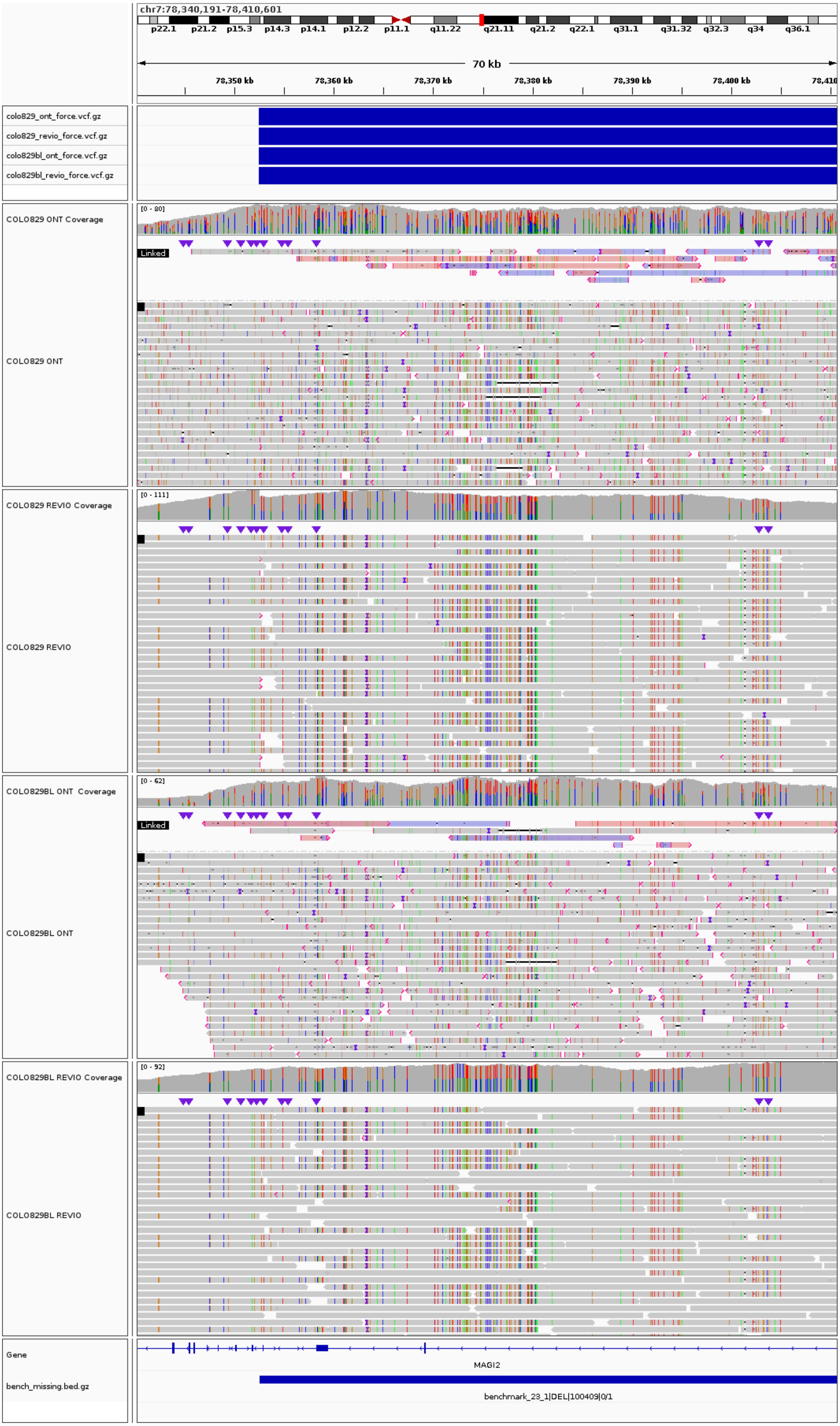

chr7:78,340,191-78,410,601
p22.1
p21.2
p15.3
p14.3
p14.1
p12.2
p11.1
q11.22
q21.11
q21.2
q22.1
q31.1
q31.32
q32.3
q34
q36.1
70 kb
78,350 kb
78,360 kb
78,370 kb
78,380 kb
78,390 kb
78,400 kb
78,410
colo829_ont_force.vcf.gz
colo829_revio_force.vcf.gz
colo829bl_ont_force.vcf.gz
colo829bl_revio_force.vcf.gz
COLO829 ONT Coverage
[0 - 80]
Linked
COLO829 ONT
COLO829 REVIO Coverage
[0 - 111]
COLO829 REVIO
COLO829BL ONT Coverage
[0 - 62]
Linked
COLO829BL ONT
COLO829BL REVIO Coverage
[0 - 92]
COLO829BL REVIO
Gene
MAGI2
bench_missing.bed.gz
benchmark_23_1|DEL|100409|0/1

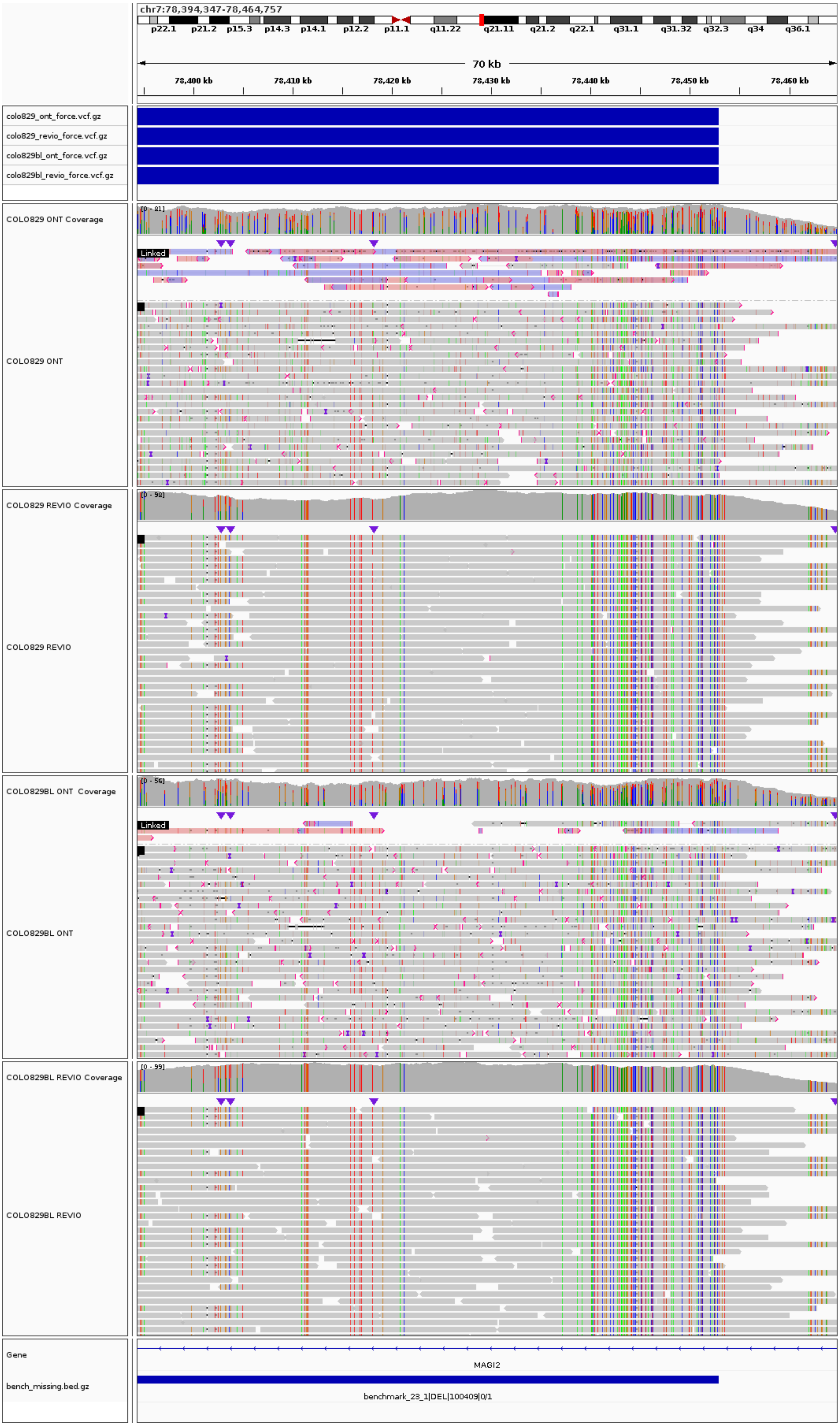
chr7:78,394,347-78,464,757
p22.1 p21.2 p15.3 p14.3 p14.1 p12.2 p11.1 q11.22 q21.11 q21.2 q22.1 q31.1 q31.32 q32.3 q34 q36.1
70 kb
78,400 kb 78,410 kb 78,420 kb 78,430 kb 78,440 kb 78,450 kb 78,460 kb
colo829_ont_force.vcf.gz
colo829_revio_force.vcf.gz
colo829bl_ont_force.vcf.gz
colo829bl_revio_force.vcf.gz
COLO829 ONT Coverage
[0 - 81]
Linked
COLO829 ONT
COLO829 REVIO Coverage
[0 - 98]
COLO829 REVIO
COLO829BL ONT Coverage
[0 - 56]
Linked
COLO829BL ONT
COLO829BL REVIO Coverage
[0 - 99]
COLO829BL REVIO
Gene
MAGI2
bench_missing.bed.gz
benchmark_23_1|DEL|100409|0/1

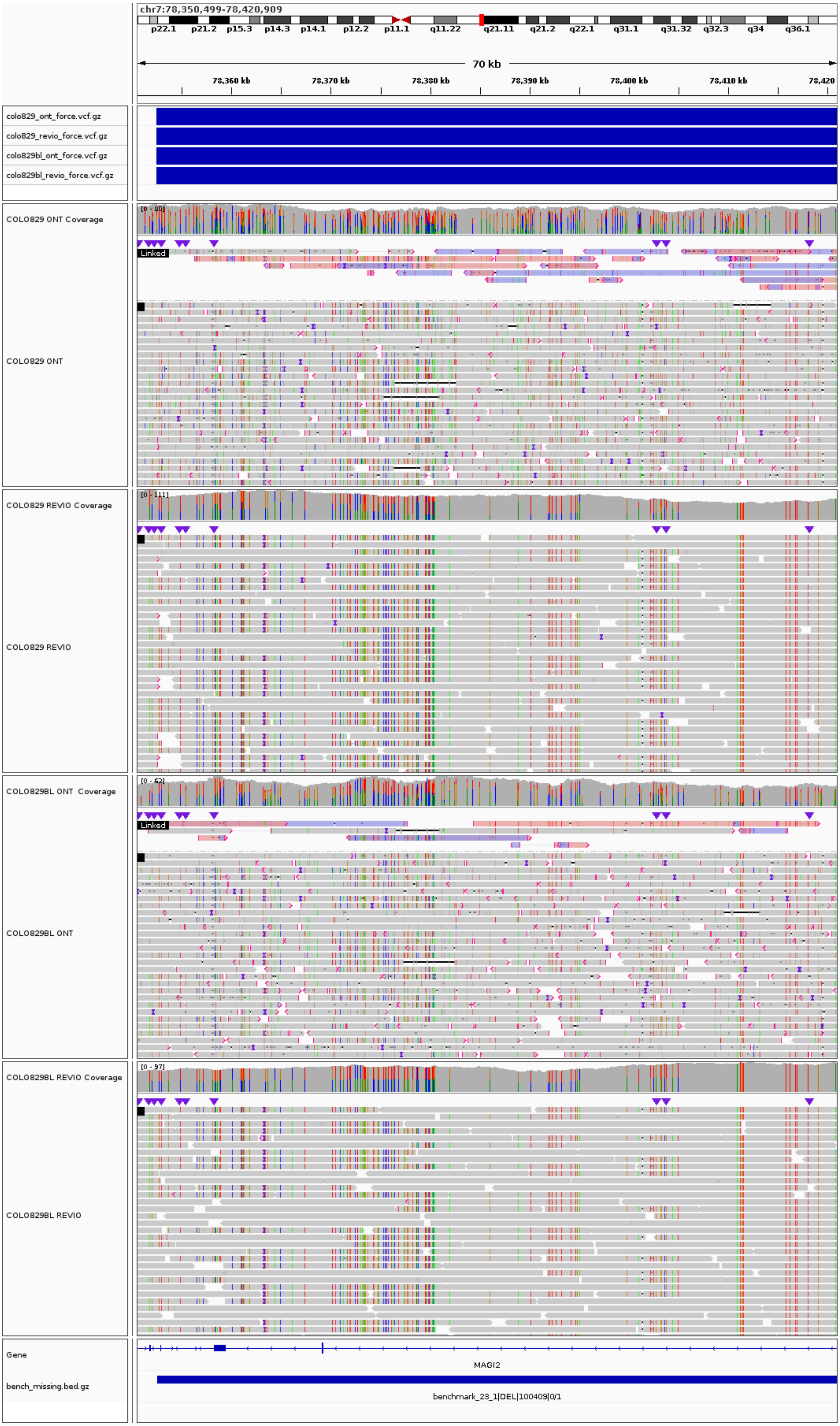
chr7:78,350,499-78,420,909
p22.1 p21.2 p15.3 p14.3 p14.1 p12.2 p11.1 q11.22 q21.11 q21.2 q22.1 q31.1 q31.32 q32.3 q34 q36.1
70 kb
78,360 kb
78,370 kb
78,380 kb
78,390 kb
78,400 kb
78,410 kb
78,420
colo829_ont_force.vcf.gz
colo829_revio_force.vcf.gz
colo829bl_ont_force.vcf.gz
colo829bl_revio_force.vcf.gz
COLO829 ONT Coverage
[0 - 80]
Linked
COLO829 ONT
COLO829 REVIO Coverage
[0 - 111]
COLO829 REVIO
COLO829BL ONT Coverage
[0 - 62]
Linked
COLO829BL ONT
COLO829BL REVIO Coverage
[0 - 97]
COLO829BL REVIO
Gene
MAGI2
bench_missing.bed.gz
benchmark_23_1|DEL|100409|0/1

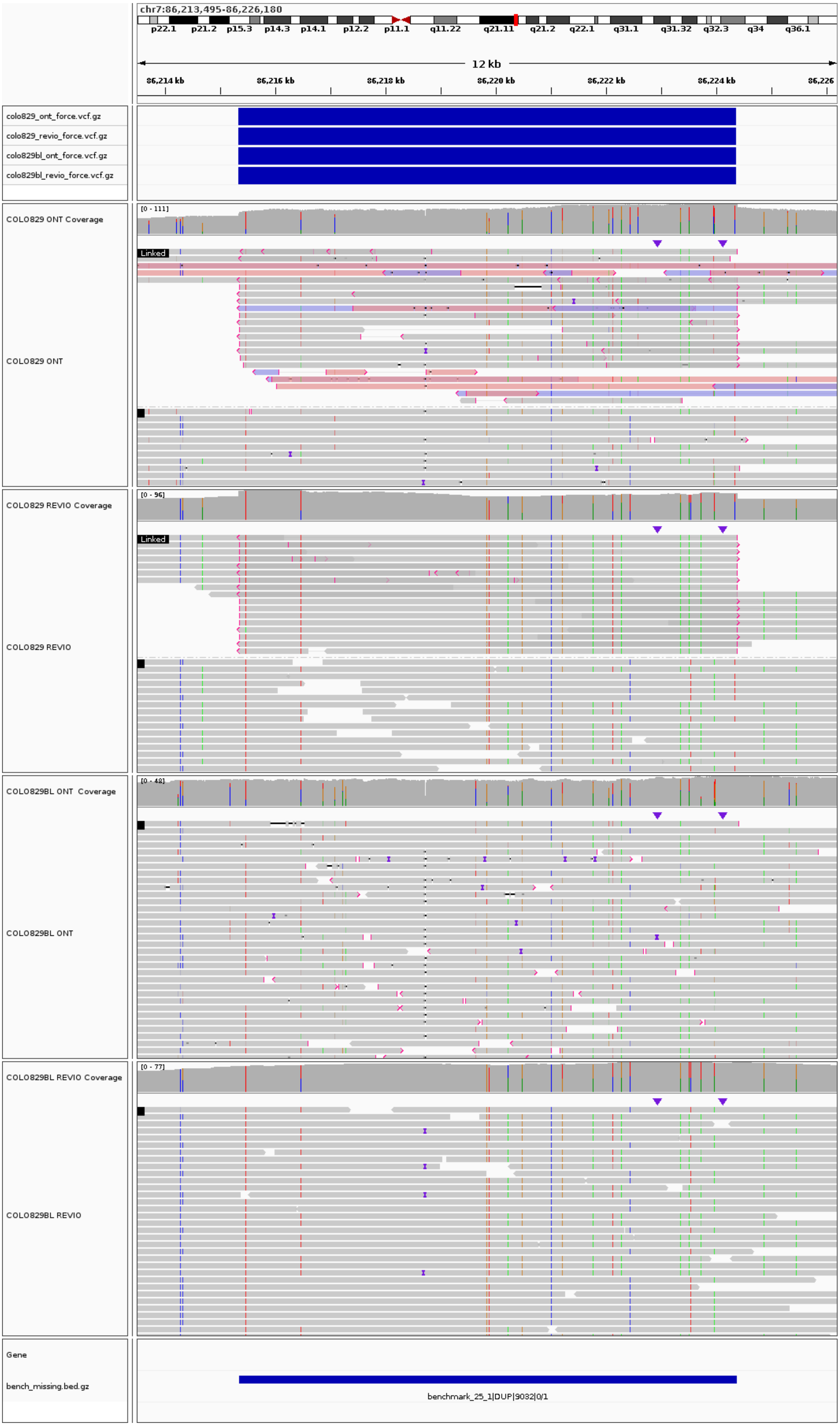
chr7:86,213,495-86,226,180
p22.1 p21.2 p15.3 p14.3 p14.1 p12.2 p11.1 q11.22 q21.11 q21.2 q22.1 q31.1 q31.32 q32.3 q34 q36.1
12 kb
86,214 kb 86,216 kb 86,218 kb 86,220 kb 86,222 kb 86,224 kb 86,226
colo829_ont_force.vcf.gz
colo829_revio_force.vcf.gz
colo829bl_ont_force.vcf.gz
colo829bl_revio_force.vcf.gz
COLO829 ONT Coverage
[0 - 111]
Linked
COLO829 ONT
COLO829 REVIO Coverage
[0 - 96]
Linked
COLO829 REVIO
COLO829BL ONT Coverage
[0 - 48]
COLO829BL ONT
COLO829BL REVIO Coverage
[0 - 77]
COLO829BL REVIO
Gene
bench_missing.bed.gz
benchmark_25_1|DUP|9032|0/1

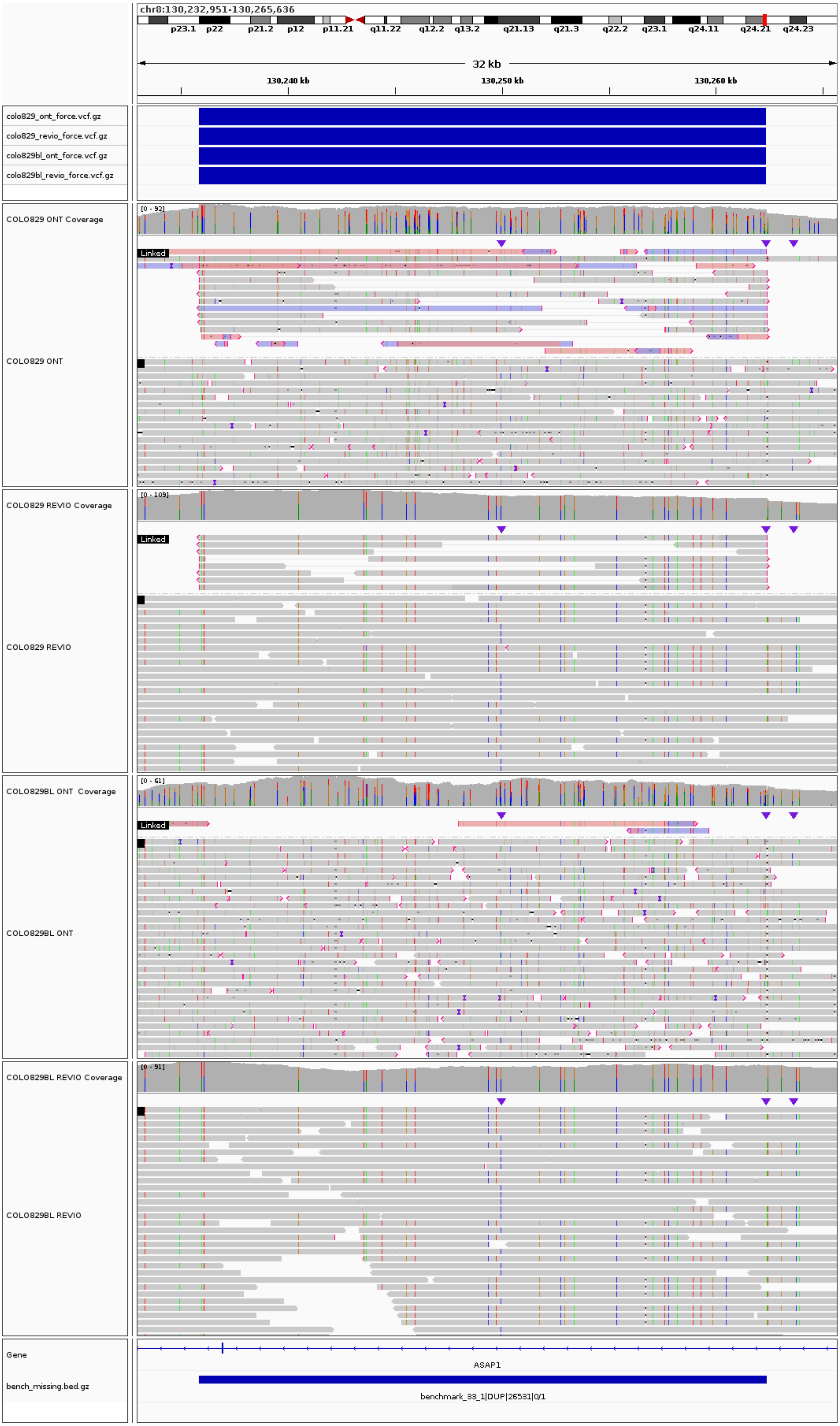
chr8:130,232,951-130,265,636
p23.1 p22 p21.2 p12 p11.21 q11.22 q12.2 q13.2 q21.13 q21.3 q22.2 q23.1 q24.11 q24.21 q24.23
32 kb
130,240 kb
130,250 kb
130,260 kb
colo829_ont_force.vcf.gz
colo829_revio_force.vcf.gz
colo829bl_ont_force.vcf.gz
colo829bl_revio_force.vcf.gz
COLO829 ONT Coverage
[0 - 92]
Linked
COLO829 ONT
COLO829 REVIO Coverage
[0 - 109]
Linked
COLO829 REVIO
COLO829BL ONT Coverage
[0 - 61]
Linked
COLO829BL ONT
COLO829BL REVIO Coverage
[0 - 51]
COLO829BL REVIO
Gene
ASAP1
bench_missing.bed.gz
benchmark_33_1|DUP|26531|0/1

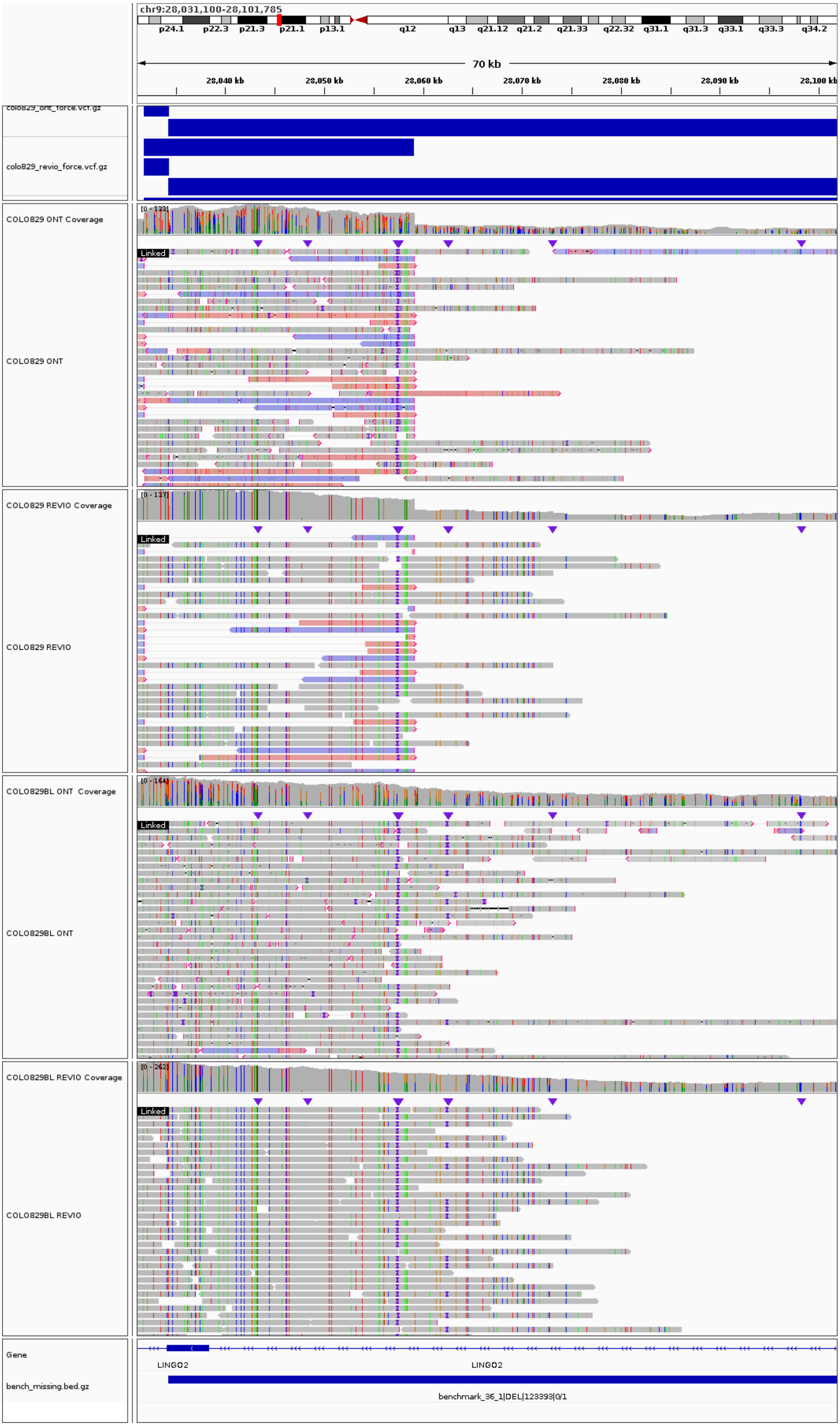
chr9:28,031,100-28,101,785
p24.1
p22.3
p21.3
p21.1
p13.1
q12
q13
q21.12
q21.2
q21.33
q22.32
q31.1
q31.3
q33.1
q33.3
q34.2
70 kb
28,040 kb
28,050 kb
28,060 kb
28,070 kb
28,080 kb
28,090 kb
28,100 kb
colo829_ont_force.vcf.gz
colo829_revio_force.vcf.gz
COLO829 ONT Coverage
[0 - 133]
Linked
COLO829 ONT
COLO829 REVIO Coverage
[0 - 137]
Linked
COLO829 REVIO
COLO829BL ONT Coverage
[0 - 164]
Linked
COLO829BL ONT
COLO829BL REVIO Coverage
[0 - 262]
Linked
COLO829BL REVIO
Gene
LINGO2
LINGO2
bench_missing.bed.gz
benchmark_36_1|DEL|123393|0/1

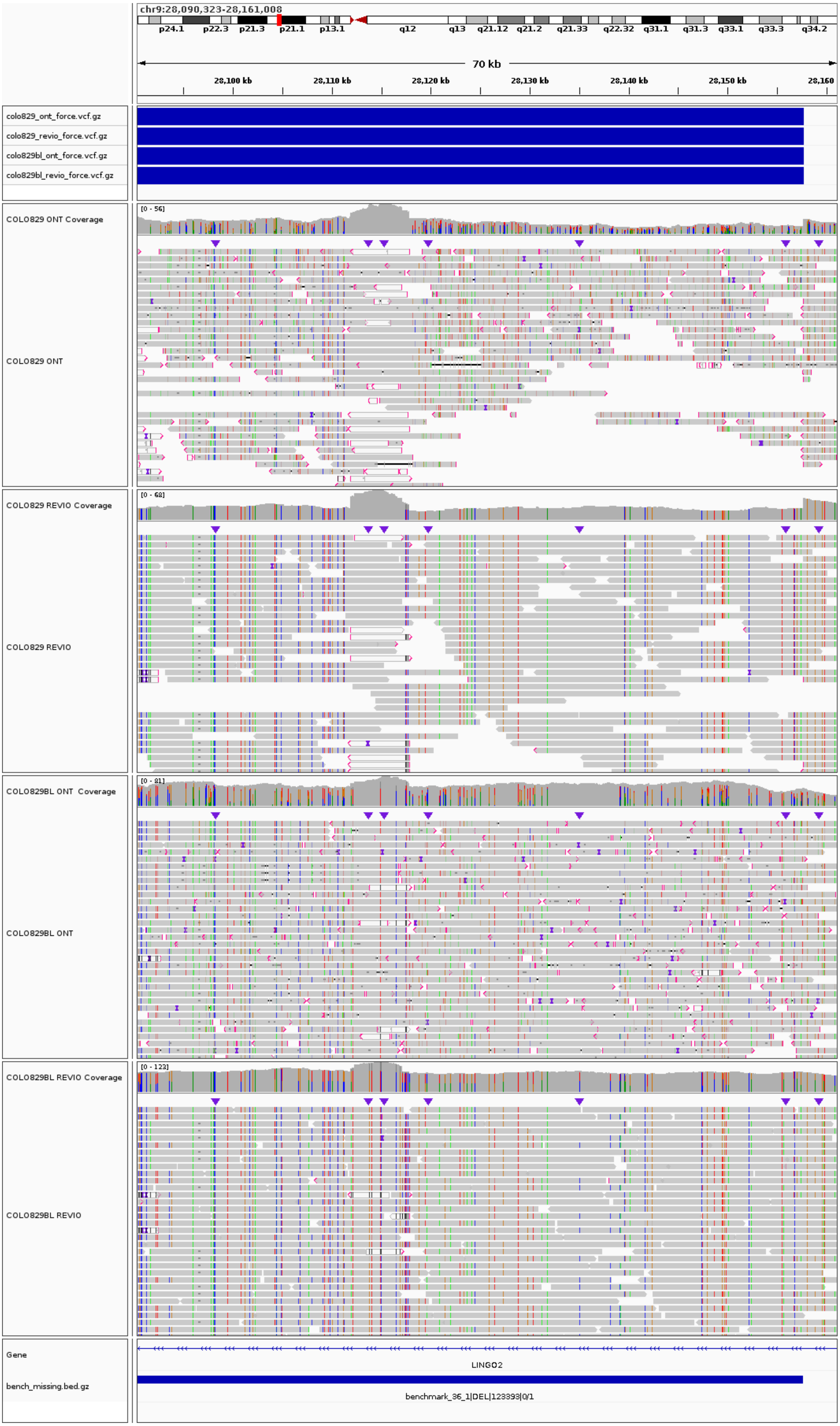
chr9:28,090,323-28,161,008
p24.1
p22.3
p21.3
p21.1
p13.1
q12
q13
q21.12
q21.2
q21.33
q22.32
q31.1
q31.3
q33.1
q33.3
q34.2
70 kb
28,100 kb
28,110 kb
28,120 kb
28,130 kb
28,140 kb
28,150 kb
28,160
colo829_ont_force.vcf.gz
colo829_revio_force.vcf.gz
colo829bl_ont_force.vcf.gz
colo829bl_revio_force.vcf.gz
COLO829 ONT Coverage
[0 - 56]
COLO829 ONT
COLO829 REVIO Coverage
[0 - 68]
COLO829 REVIO
COLO829BL ONT Coverage
[0 - 81]
COLO829BL ONT
COLO829BL REVIO Coverage
[0 - 123]
COLO829BL REVIO
Gene
LINGO2
bench_missing.bed.gz
benchmark_36_1|DEL|123393|0/1

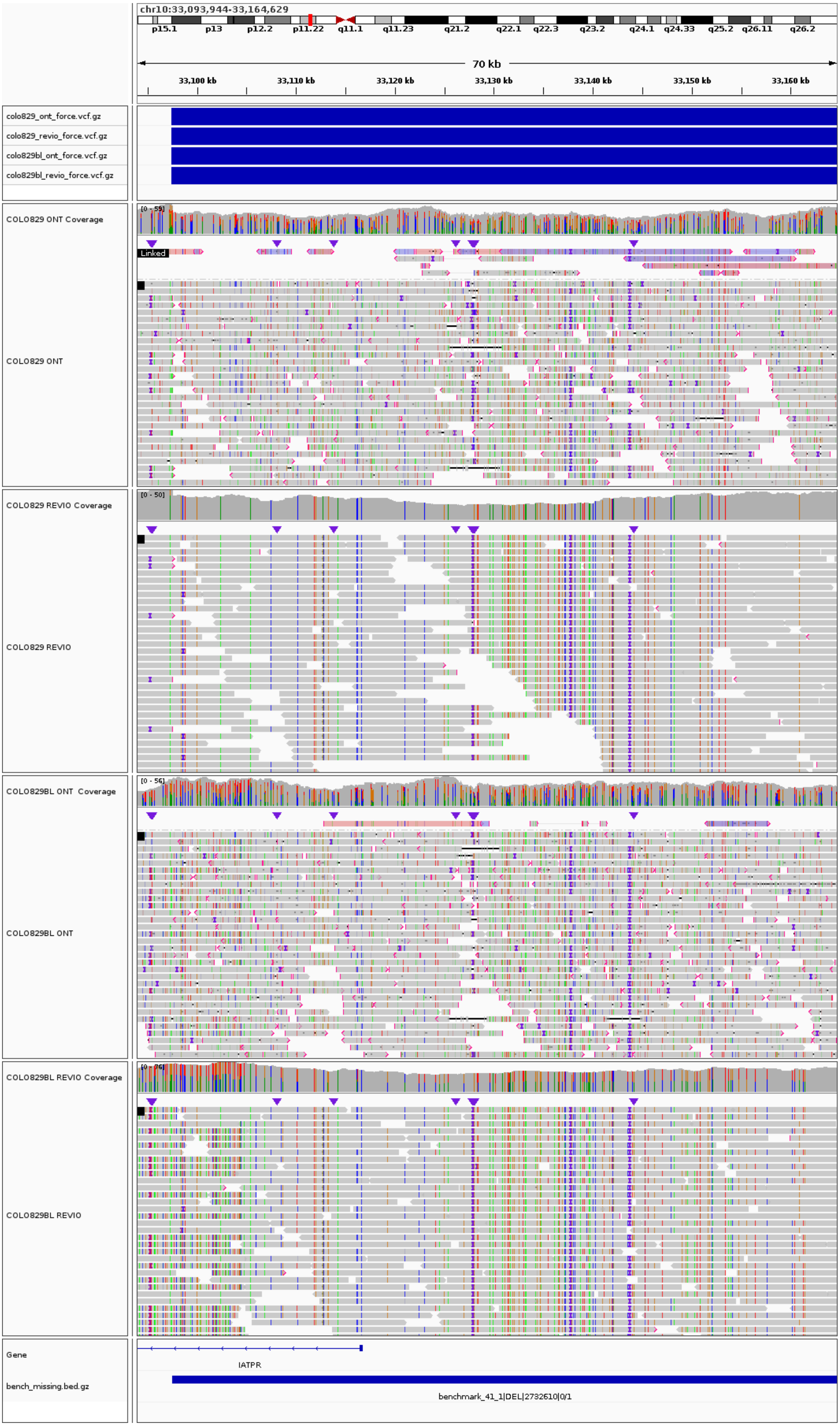
chr10:33,093,944-33,164,629
p15.1
p13
p12.2
p11.22
q11.1
q11.23
q21.2
q22.1
q22.3
q23.2
q24.1
q24.33
q25.2
q26.11
q26.2
70 kb
33,100 kb
33,110 kb
33,120 kb
33,130 kb
33,140 kb
33,150 kb
33,160 kb
colo829_ont_force.vcf.gz
colo829_revio_force.vcf.gz
colo829bl_ont_force.vcf.gz
colo829bl_revio_force.vcf.gz
COLO829 ONT Coverage
[0 - 59]
Linked
COLO829 ONT
COLO829 REVIO Coverage
[0 - 50]
COLO829 REVIO
COLO829BL ONT Coverage
[0 - 56]
COLO829BL ONT
COLO829BL REVIO Coverage
COLO829BL REVIO
Gene
IATPR
bench_missing.bed.gz
benchmark_41_1|DEL|2732610|0/1

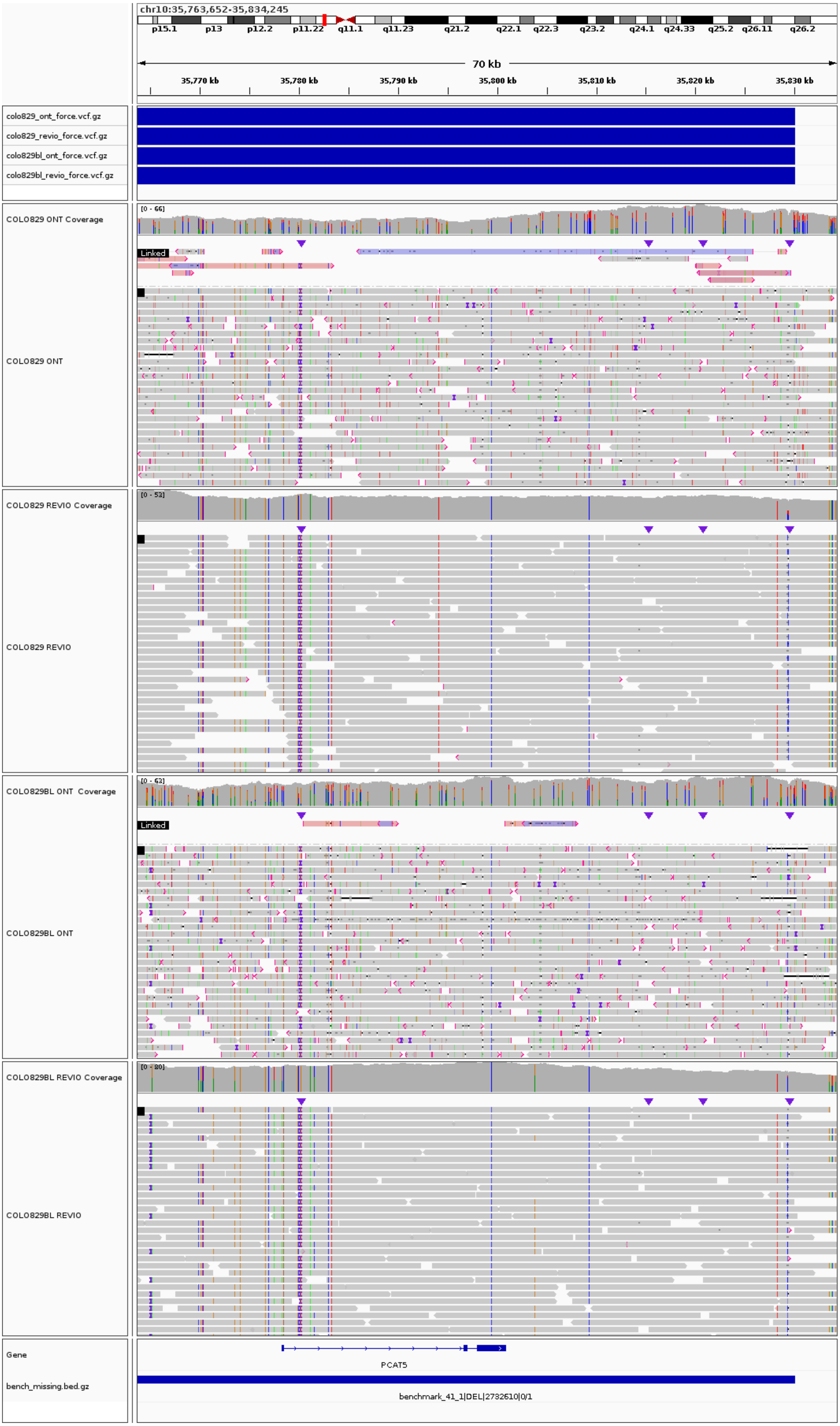
chr10:35,763,652-35,834,245
p15.1
p13
p12.2
p11.22
q11.1
q11.23
q21.2
q22.1
q22.3
q23.2
q24.1
q24.33
q25.2
q26.11
q26.2
70 kb
35,770 kb
35,780 kb
35,790 kb
35,800 kb
35,810 kb
35,820 kb
35,830 kb
colo829_ont_force.vcf.gz
colo829_revio_force.vcf.gz
colo829bl_ont_force.vcf.gz
colo829bl_revio_force.vcf.gz
COLO829 ONT Coverage
[0 - 66]
Linked
COLO829 ONT
COLO829 REVIO Coverage
[0 - 53]
COLO829 REVIO
COLO829BL ONT Coverage
[0 - 63]
Linked
COLO829BL ONT
COLO829BL REVIO Coverage
[0 - 80]
COLO829BL REVIO
Gene
PCAT5
bench_missing.bed.gz
benchmark_41_1|DEL|2732610|0/1

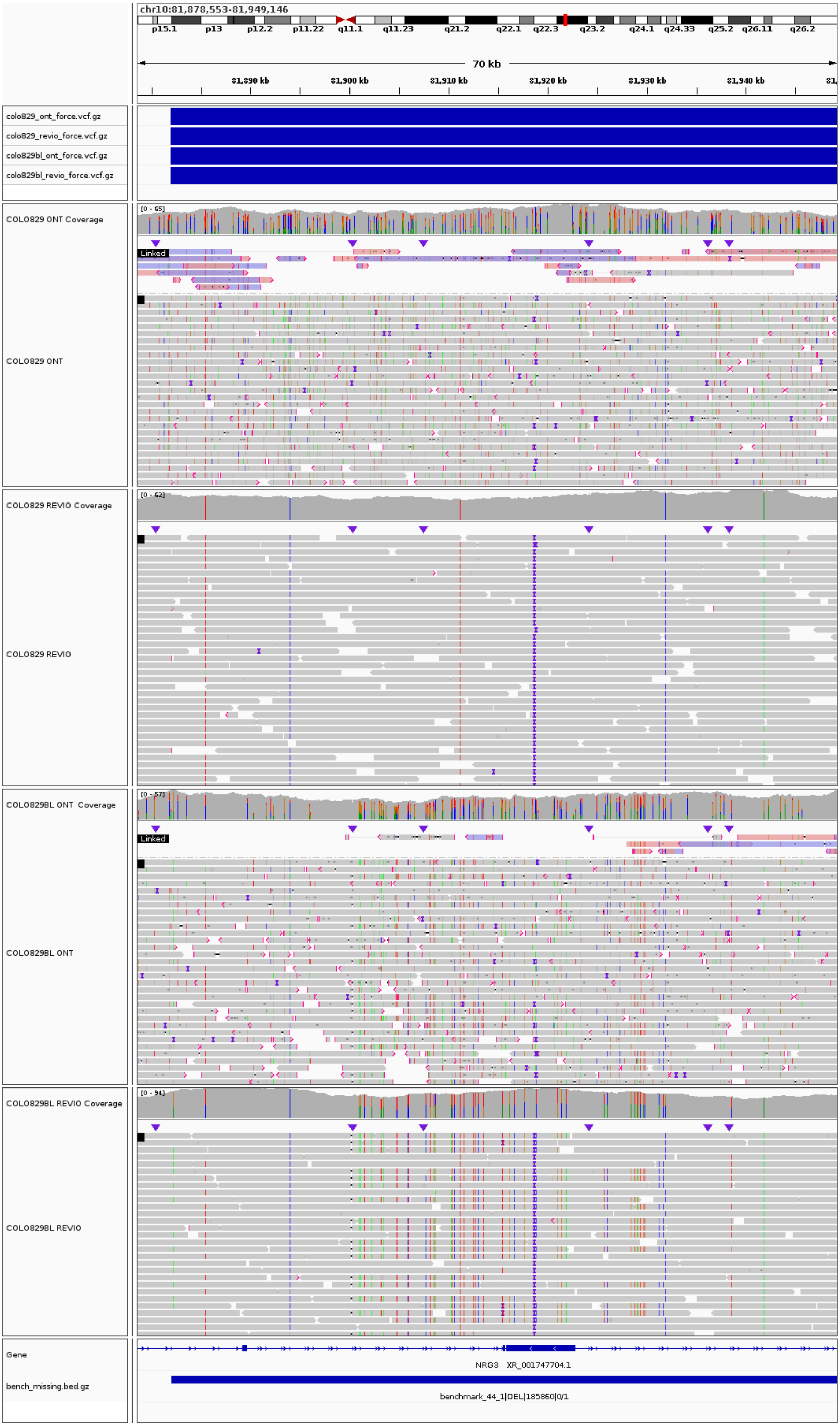

chr10:81,878,553-81,949,146
p15.1
p13
p12.2
p11.22
q11.1
q11.23
q21.2
q22.1
q22.3
q23.2
q24.1
q24.33
q25.2
q26.11
q26.2
70 kb
81,890 kb
81,900 kb
81,910 kb
81,920 kb
81,930 kb
81,940 kb
colo829_ont_force.vcf.gz
colo829_revio_force.vcf.gz
colo829bl_ont_force.vcf.gz
colo829bl_revio_force.vcf.gz
COLO829 ONT Coverage
[0 - 65]
Linked
COLO829 ONT
COLO829 REVIO Coverage
[0 - 62]
COLO829 REVIO
COLO829BL ONT Coverage
[0 - 57]
COLO829BL ONT
COLO829BL REVIO Coverage
[0 - 94]
COLO829BL REVIO
Gene
NRG3
XR_001747704.1
bench_missing.bed.gz
benchmark_44_1|DEL|185860|0/1

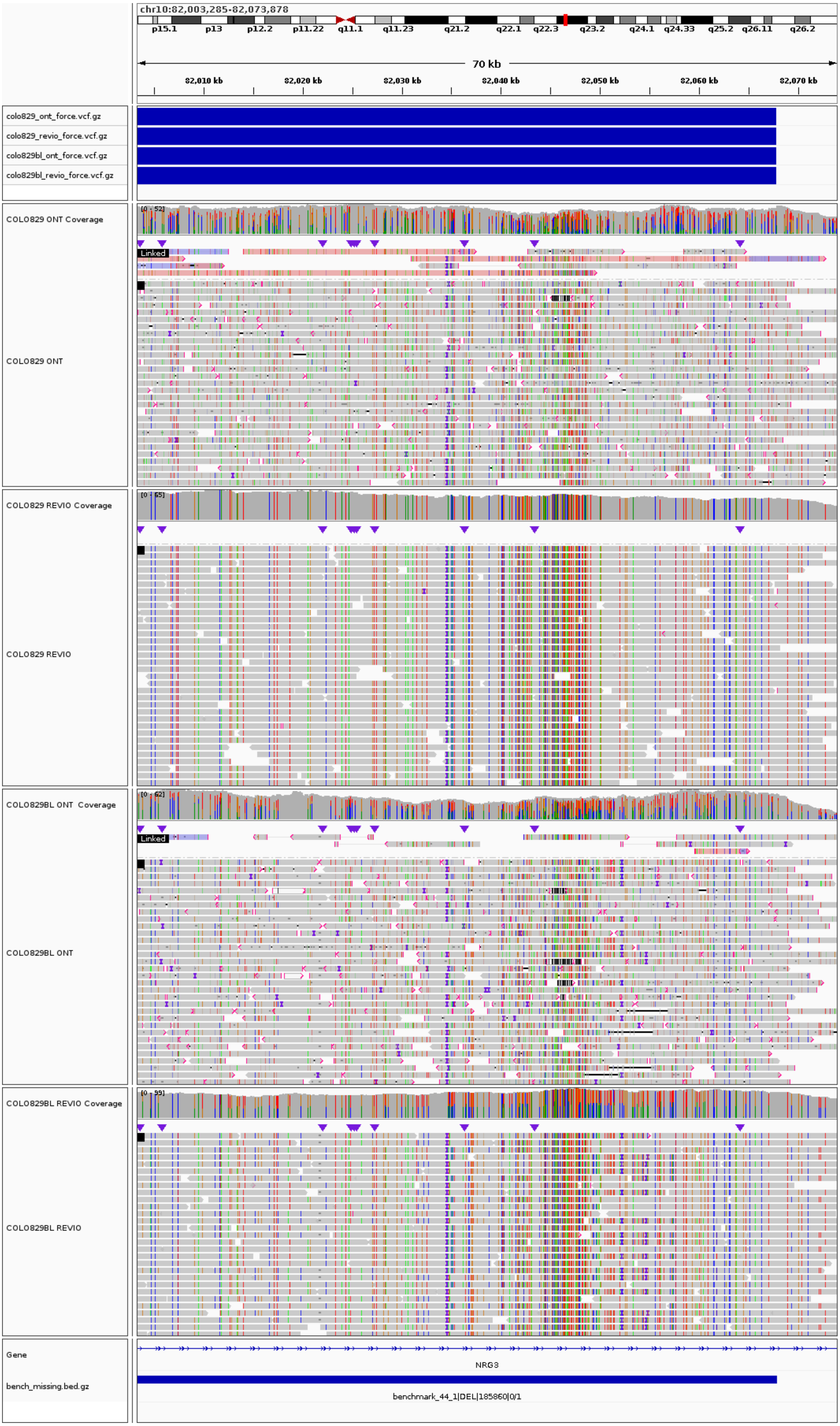
chr10:82,003,285-82,073,878
p15.1 p13 p12.2 p11.22 q11.1 q11.23 q21.2 q22.1 q22.3 q23.2 q24.1 q24.33 q25.2 q26.11 q26.2
70 kb
82,010 kb 82,020 kb 82,030 kb 82,040 kb 82,050 kb 82,060 kb 82,070 kb
colo829_ont_force.vcf.gz
colo829_revio_force.vcf.gz
colo829bl_ont_force.vcf.gz
colo829bl_revio_force.vcf.gz
COLO829 ONT Coverage
[0 - 52]
Linked
COLO829 ONT
COLO829 REVIO Coverage
[0 - 55]
COLO829 REVIO
COLO829BL ONT Coverage
[0 - 62]
Linked
COLO829BL ONT
COLO829BL REVIO Coverage
[0 - 59]
COLO829BL REVIO
Gene
NRG3
bench_missing.bed.gz
benchmark_44_1|DEL|185860|0/1

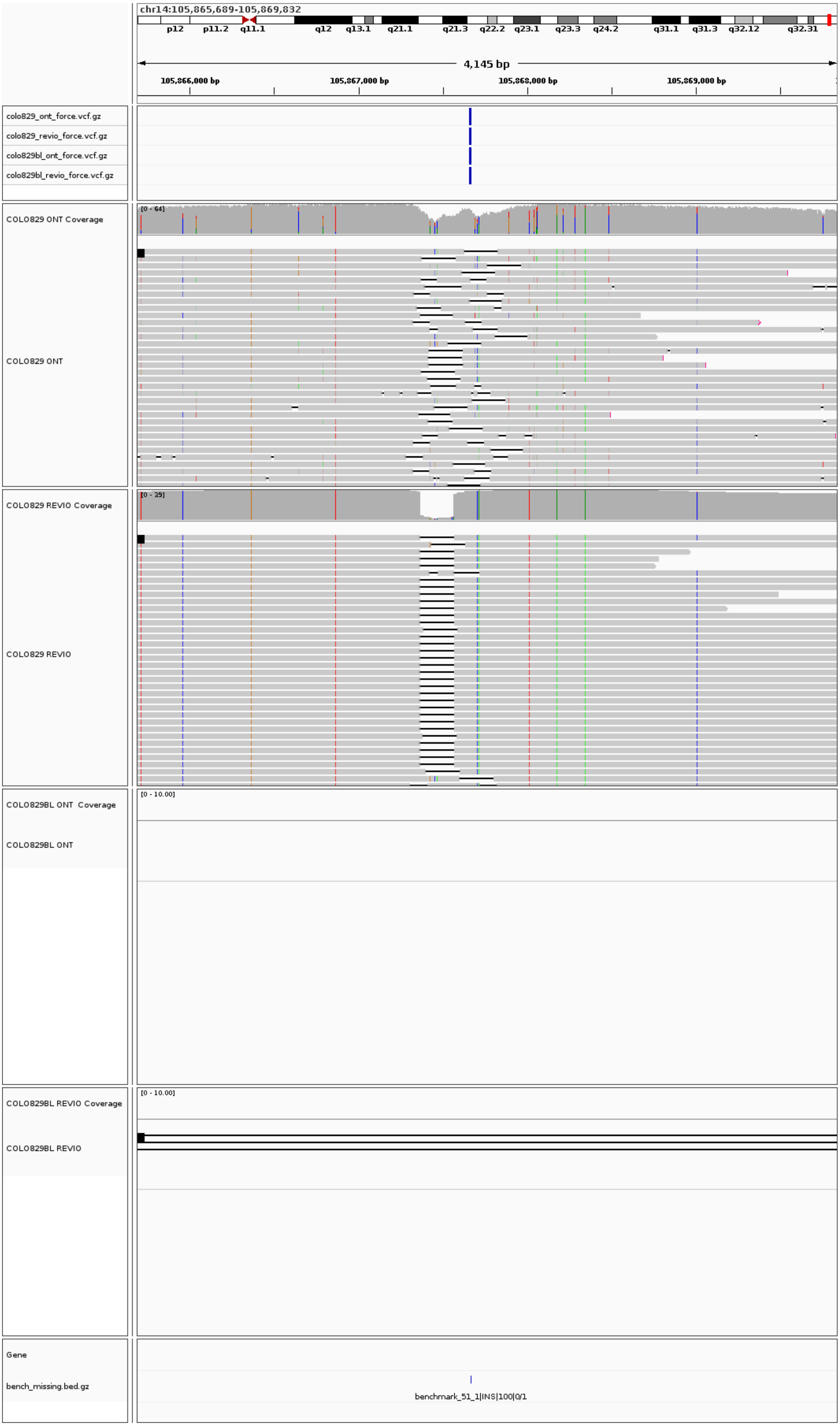

chr14:105,865,689-105,869,832
p12
p11.2
q11.1
q12
q13.1
q21.1
q21.3
q22.2
q23.1
q23.3
q24.2
q31.1
q31.3
q32.12
q32.31
4,145 bp
105,866,000 bp
105,867,000 bp
105,868,000 bp
105,869,000 bp
colo829_ont_force.vcf.gz
colo829_revio_force.vcf.gz
colo829bl_ont_force.vcf.gz
colo829bl_revio_force.vcf.gz
COLO829 ONT Coverage
[0 - 64]
COLO829 ONT
COLO829 REVIO Coverage
[0 - 39]
COLO829 REVIO
COLO829BL ONT Coverage
[0 - 10.00]
COLO829BL ONT
COLO829BL REVIO Coverage
[0 - 10.00]
COLO829BL REVIO
Gene
bench_missing.bed.gz
benchmark_51_1|INS|100|0/1

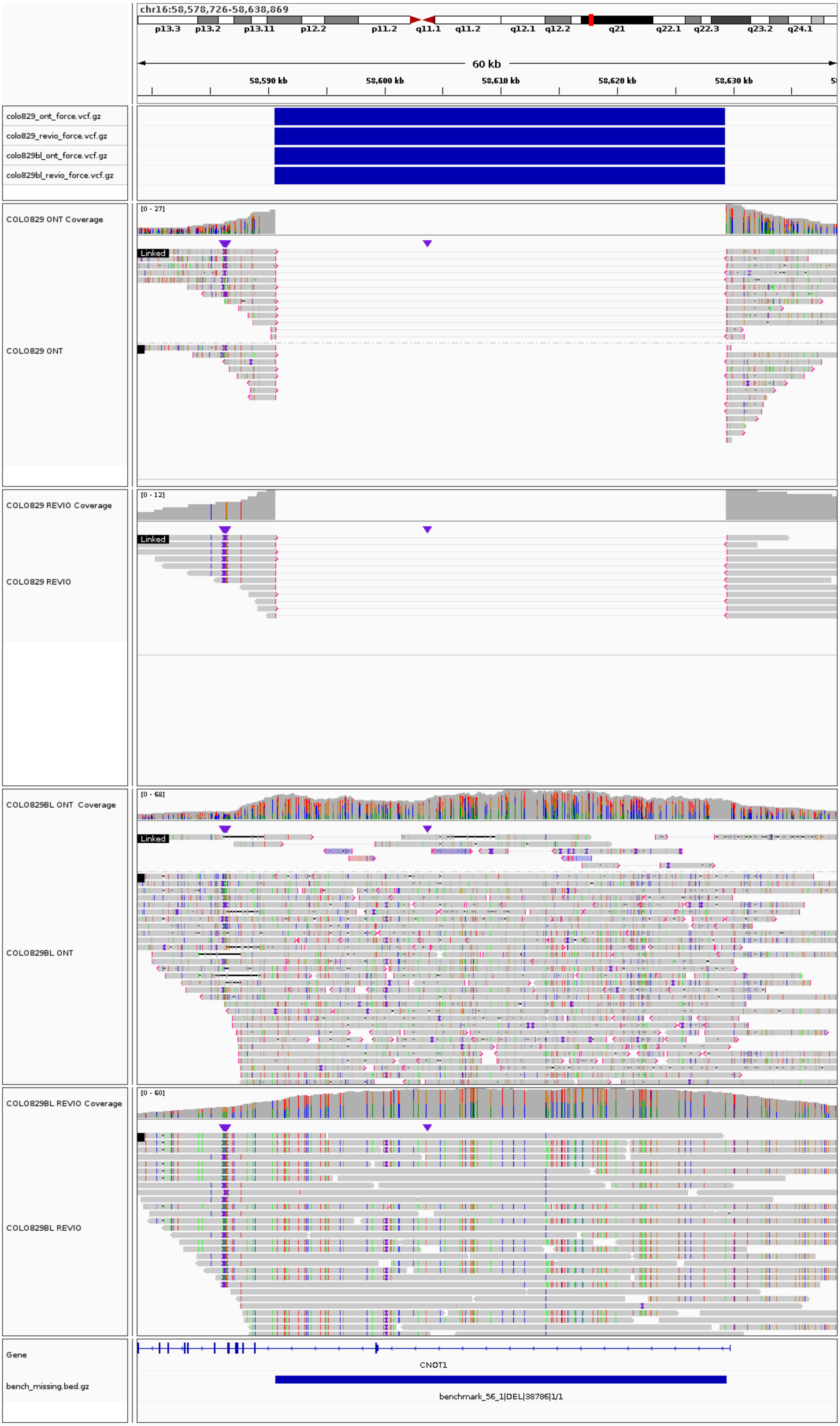

chr16:58,578,726-58,638,869
p13.3
p13.2
p13.11
p12.2
p11.2
q11.1
q11.2
q12.1
q12.2
q21
q22.1
q22.3
q23.2
q24.1
60 kb
58,590 kb
58,600 kb
58,610 kb
58,620 kb
58,630 kb
colo829_ont_force.vcf.gz
colo829_revio_force.vcf.gz
colo829bl_ont_force.vcf.gz
colo829bl_revio_force.vcf.gz
COLO829 ONT Coverage
[0 - 27]
COLO829 ONT
Linked
COLO829 REVIO Coverage
[0 - 12]
COLO829 REVIO
Linked
COLO829BL ONT Coverage
[0 - 68]
COLO829BL ONT
Linked
COLO829BL REVIO Coverage
[0 - 60]
COLO829BL REVIO
Gene
CNOT1
bench_missing.bed.gz
benchmark_56_1|DEL|38786|1/1

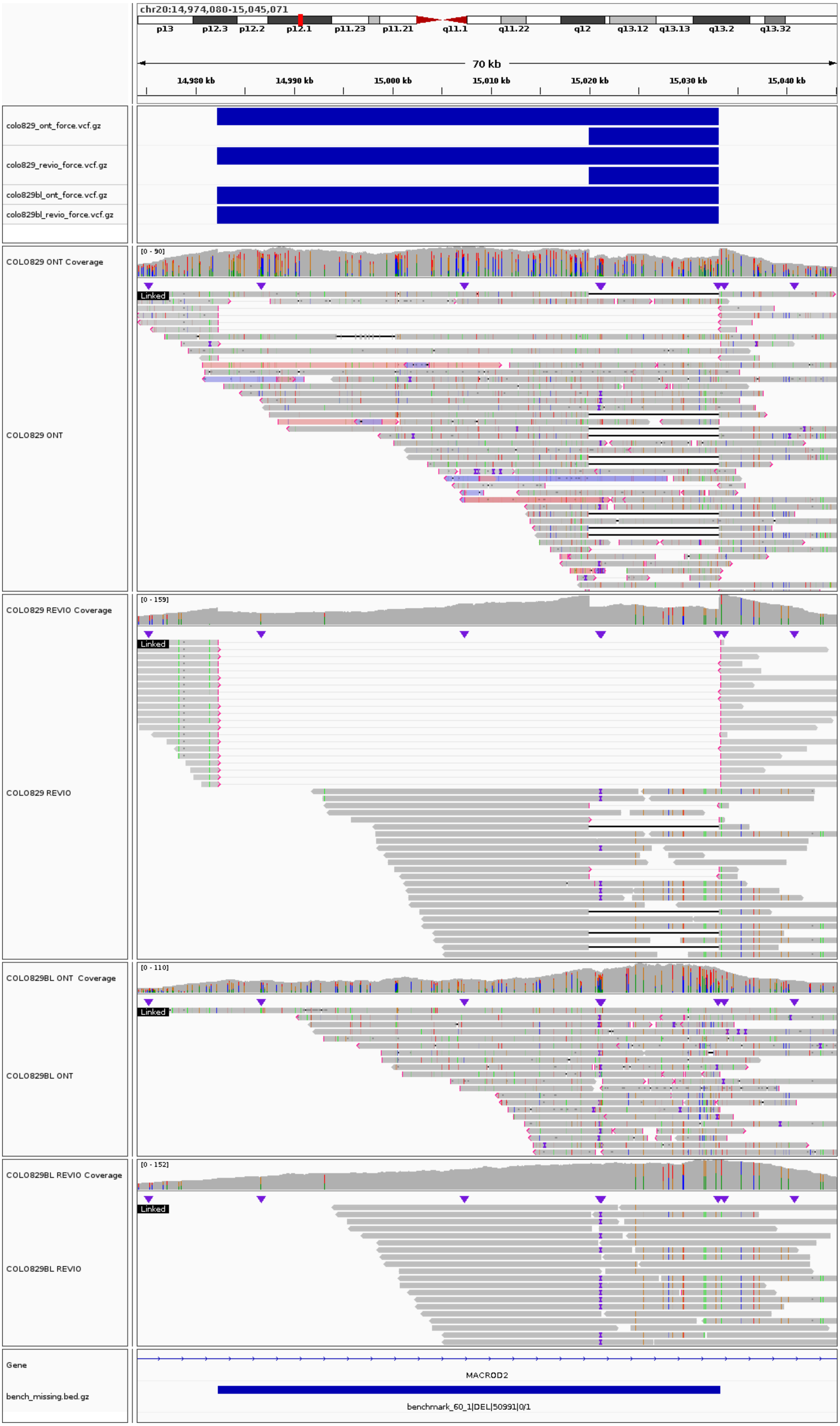

chr20:14,974,080-15,045,071
p13
p12.3
p12.2
p12.1
p11.23
p11.21
q11.1
q11.22
q12
q13.12
q13.13
q13.2
q13.32
70 kb
14,980 kb
14,990 kb
15,000 kb
15,010 kb
15,020 kb
15,030 kb
15,040 kb
colo829_ont_force.vcf.gz
colo829_revio_force.vcf.gz
colo829bl_ont_force.vcf.gz
colo829bl_revio_force.vcf.gz
COLO829 ONT Coverage
[0 - 90]
Linked
COLO829 ONT
COLO829 REVIO Coverage
[0 - 159]
COLO829 REVIO
COLO829BL ONT Coverage
[0 - 110]
COLO829BL ONT
COLO829BL REVIO Coverage
[0 - 152]
COLO829BL REVIO
Gene
MACROD2
bench_missing.bed.gz
benchmark_60_1|DEL|50991|0/1

Supplementary Figure 12. IGV screenshots of the 13 missing SVs from the COLO829 benchmark (see **Supplementary table 18**)

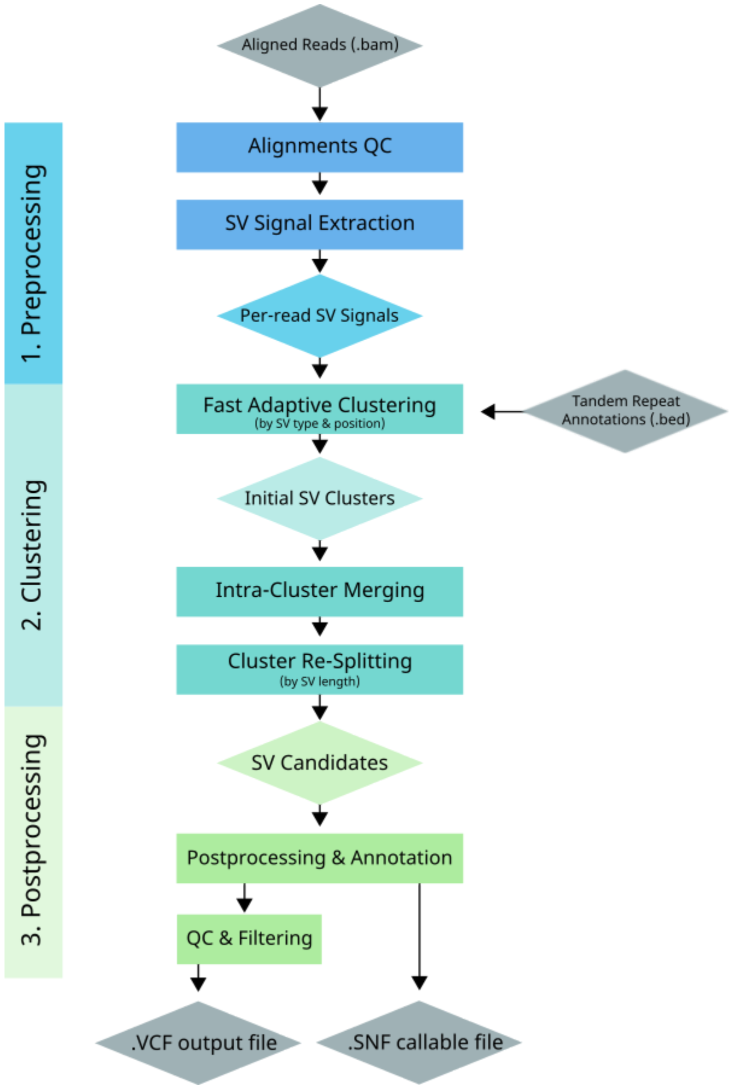

Supplementary Figure 13

Steps in the Sniffles2 germline mode algorithm

SAMPLE_1.snf	SAMPLE_2.snf	...	SAMPLE_N.snf
INDEX	INDEX	...	INDEX
Block 1: chr1 0-10000<br><br>SVCand1 (INS)<br>SVCand2 (DEL)<br>...<br>SVCandN (DUP)<br><br>Coverage Track	Block 1: chr1 0-10000<br><br>SVCand1 (DEL)<br>SVCand2 (INS)<br>...<br>SVCandN (INS)<br><br>Coverage Track	...	Block 1: chr1 0-10000<br><br>SVCand1 (DUP)<br>SVCand2 (BND)<br>...<br>SVCandN (INS)<br><br>Coverage Track
Block 2: chr1 10000-20000	Block 2: chr1 10000-20000	...	Block 2: chr1 10000-20000
...	...	...	...
Block n	Block n	...	Block n

Supplementary Figure 14

Schematic for the SNF file format

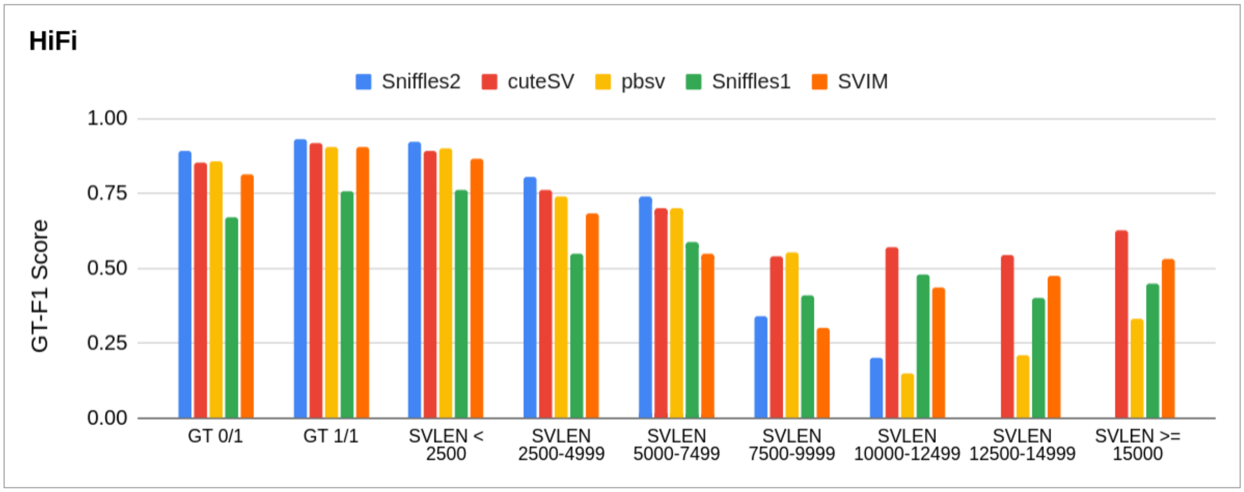

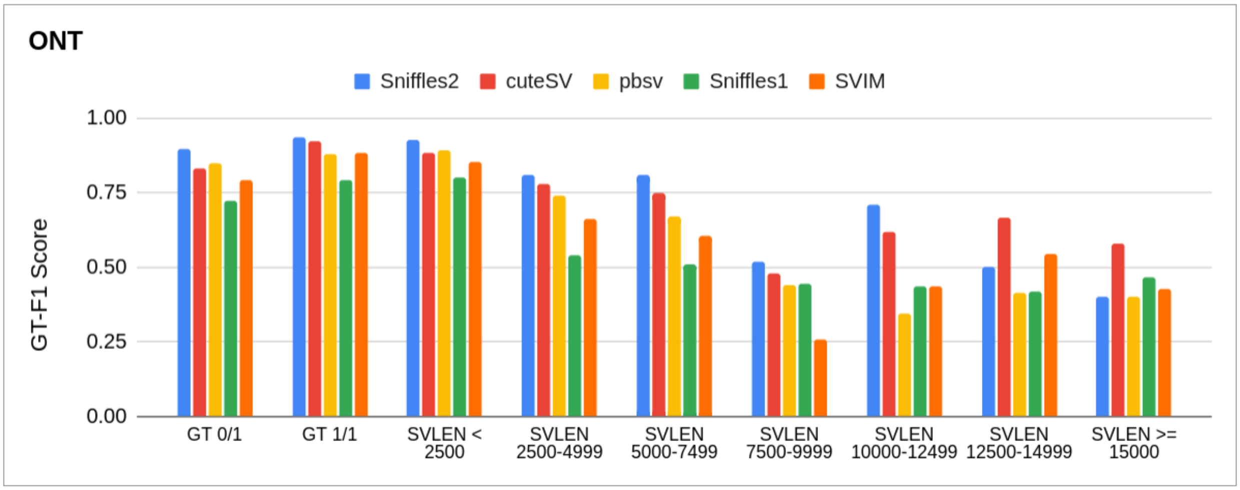

Supplementary Figure 15

Genotype F1 measure for the detection of insertions and deletions by genotype and SV length. Measured by using the Truvari summary output files. For the evaluation of each caller we used the GIAB Oxford Nanopore and Pacbio HiFi 30x coverage titration benchmark. All callers were benchmarked using default parameters. The plot shows F1-genotype measure (Y-axis) per category (X-axis). The complete results are shown in **Supplementary Table 19.** Notice that over 94% of the SV are of smaller to 2500 bases (absolute size).

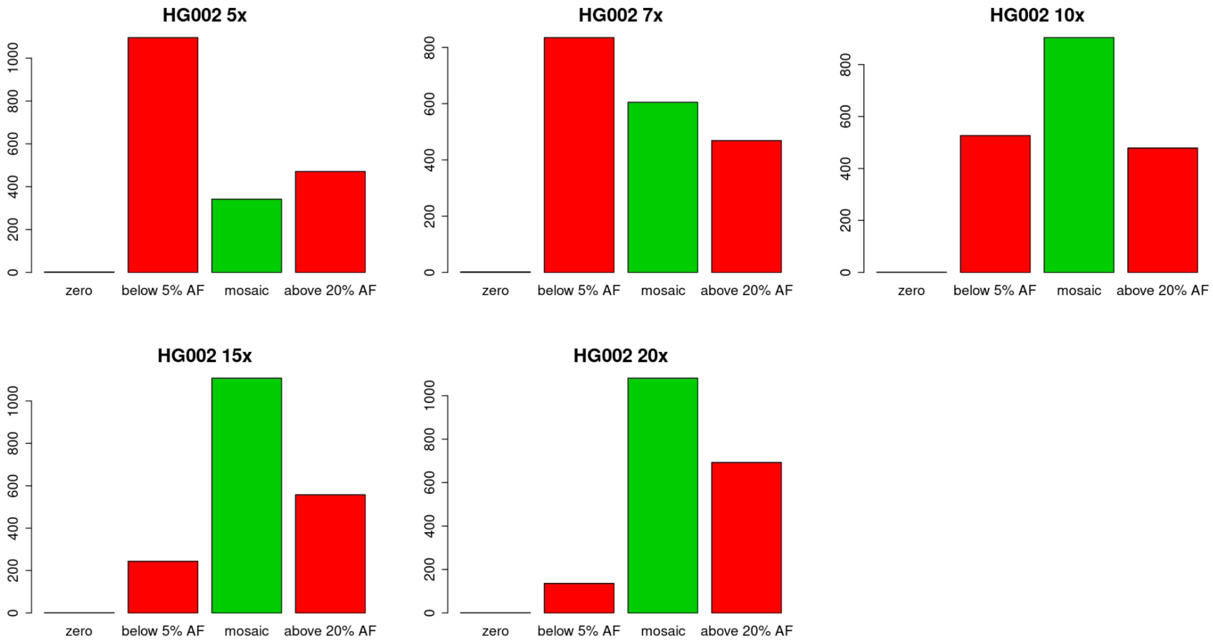

Supplementary Figure 16

Distribution of SV from HG002 by their AF in each of the five tested titrations (5x, 7x, 10x, 15x, 20x). In green are those that Sniffles2 mosaic mode can detect, the rest will not be assessed nor reported. Notice that even at 5x, some SVs do have AF greater than 20%.
