# Supplementary material for: Detection of mosaic and population-level structural variants with Sniffles2
Source: Nat Biotechnol. 2024 Jan 2;42(10):1571–80. doi: 10.1038/s41587-023-02024-y (PMC11217151; doi:10.1038/s41587-023-02024-y)
Supplement: Supplementary file 2 [file 41587_2023_2024_MOESM2_ESM.pdf]

Corresponding author(s): Sedlazeck Fritz

Last updated by author(s): Aug 30, 2023

Reporting Summary

Nature Portfolio wishes to improve the reproducibility of the work that we publish. This form provides structure for consistency and transparency in reporting. For further information on Nature Portfolio policies, see our Editorial Policies and the Editorial Policy Checklist.

Statistics

For all statistical analyses, confirm that the following items are present in the figure legend, table legend, main text, or Methods section.

n/a	Confirmed	
☐	☒	The exact sample size (*n*) for each experimental group/condition, given as a discrete number and unit of measurement
☐	☒	A statement on whether measurements were taken from distinct samples or whether the same sample was measured repeatedly
☒	☐	The statistical test(s) used AND whether they are one- or two-sided<br>*Only common tests should be described solely by name; describe more complex techniques in the Methods section.*
☒	☐	A description of all covariates tested
☒	☐	A description of any assumptions or corrections, such as tests of normality and adjustment for multiple comparisons
☐	☒	A full description of the statistical parameters including central tendency (e.g. means) or other basic estimates (e.g. regression coefficient) AND variation (e.g. standard deviation) or associated estimates of uncertainty (e.g. confidence intervals)
☒	☐	For null hypothesis testing, the test statistic (e.g. *F*, *t*, *r*) with confidence intervals, effect sizes, degrees of freedom and *P* value noted<br>*Give P values as exact values whenever suitable.*
☒	☐	For Bayesian analysis, information on the choice of priors and Markov chain Monte Carlo settings
☒	☐	For hierarchical and complex designs, identification of the appropriate level for tests and full reporting of outcomes
☒	☐	Estimates of effect sizes (e.g. Cohen's *d*, Pearson's *r*), indicating how they were calculated

Our web collection on statistics for biologists contains articles on many of the points above.

Software and code

Policy information about availability of computer code

Data collection

We have now listed all programs with github links, versions and names in the supplementary table 20. the methods are all cited in the methods or main text.

Software Name Version Github URL
minimap2 v2.17-r941 https://github.com/lh3/minimap2
samtools v1.13 http://htslib.org/
mosdepth v0.3.2 https://github.com/brentp/mosdepth
dipcall v0.2 https://github.com/lh3/dipcall
Truvari v2.1 https://github.com/ACEnglish/truvari
SVIM v1.4.2 https://github.com/eldariont/svim
Sniffles v1.12 https://github.com/fritzsedlazeck/Sniffles
cuteSV v1.0.11 https://github.com/tjiangHIT/cuteSV
pbsv v2.6.2 https://github.com/PacificBiosciences/pbsv
SnpSift v4.3t http://snpeff.sourceforge.net/SnpSift.html
SURVIVOR v1.0.7 https://github.com/fritzsedlazeck/SURVIVOR
Biopython v1.79 http://biopython.org
Saphyr Gen2 https://bionanogenomics.com/products/saphyr/
Bionano Solve v3.6 https://bionanogenomics.com/technology/genome-assembly/
bwa mem v0.7.17-r1188 http://bio-bwa.sourceforge.net/
manta v1.6.0 https://getmanta.com/

svtyper v0.7.1 https://github.com/hall-lab/svtyper
BCFtools v1.14 http://samtools.sourceforge.net/mpileup.shtml

Data analysis

Source code for Sniffles2 is available at https://github.com/fritzsedlazeck/Sniffles and https://doi.org/10.5281/zenodo.8121996 the auxiliary scripts are available at https://github.com/smolkmo/Sniffles2-Supplement and https://doi.org/10.5281/zenodo.8122060

For manuscripts utilizing custom algorithms or software that are central to the research but not yet described in published literature, software must be made available to editors and reviewers. We strongly encourage code deposition in a community repository (e.g. GitHub). See the Nature Portfolio guidelines for submitting code & software for further information.

Data

Policy information about availability of data

All manuscripts must include a data availability statement. This statement should provide the following information, where applicable:

- Accession codes, unique identifiers, or web links for publicly available datasets
- A description of any restrictions on data availability
- For clinical datasets or third party data, please ensure that the statement adheres to our policy

GIAB HG002 Pacbio HiFi data is hosted at the github server: https://ftp-trace.ncbi.nlm.nih.gov/ReferenceSamples/giab/data/AshkenazimTrio/HG002_NA24385_son/PacBio_CCS_15kb/
ONT HG002: https://labs.epi2me.io/gm24385_q20_2021.10/
ONT HG00733: https://www.internationalgenome.org/data-portal/search?q=HG00733 and https://ftp.hgsc.bcm.edu/Software/Truvari/3.1/sample_vcfs/hg19/li/HG00733.vcf.gz
GIAB benchmark sets:
Genome wide: https://ftp-trace.ncbi.nlm.nih.gov/ReferenceSamples/giab/release/AshkenazimTrio/HG002_NA24385_son/NIST_SV_v0.6/
Medical regions: https://ftp-trace.ncbi.nlm.nih.gov/ReferenceSamples/giab/release/AshkenazimTrio/HG002_NA24385_son/CMRG_v1.00/

The 1000 genomes data sets of the three genomes were downloaded from : https://github.com/human-pangenomics/hpgp-data The dipcall results that we leveraged as benchmark are deposited at https://github.com/smolkmo/Sniffles2-Supplement

The other data sets have been made available over SRA. 31 Oxford Nanopore data sets that represent cases of Mendelian disorders have SRA bioproject ID PRJNA953021. MSA sample has bioproject ID PRJNA985263. The COLO829BL (normal) and COLO829 (tumor) ONT samples can be found with the ENA ID: PRJEB27698 (samples ERR2752451 and ERR2752452 respectively), and the Revio tumor/normal samples can be found in https://downloads.pacbcloud.com/public/revio/2023Q2/COLO829/

Research involving human participants, their data, or biological material

Policy information about studies with human participants or human data. See also policy information about sex, gender (identity/presentation), and sexual orientation and race, ethnicity and racism.

Reporting on sex and gender	MSA sample: Male ; Mendelian samples gender information is reported in Table 1 in the main text
Reporting on race, ethnicity, or other socially relevant groupings	MSA sample: british, no other info available<br>The Mendelian samples dont have reported race
Population characteristics	N/a for all participants. Population characteristics weren't recorded.
Recruitment	MSA: Sample provided by Queen Square Brain Bank, UCL, UK. Had consented to brain donation and research
Ethics oversight	MSA: Ethics oversight London Central Ethics Research Committee (UK) REF /LO/0044

Note that full information on the approval of the study protocol must also be provided in the manuscript.

Field-specific reporting

Please select the one below that is the best fit for your research. If you are not sure, read the appropriate sections before making your selection.

☒ Life sciences ☐ Behavioural & social sciences ☐ Ecological, evolutionary & environmental sciences

For a reference copy of the document with all sections, see nature.com/documents/nr-reporting-summary-flat.pdf

Life sciences study design

All studies must disclose on these points even when the disclosure is negative.

Sample size	41 samples

Data exclusions	No samples were excluded as not applicable for study type.
Replication	No replicates were produced as all these samples were genomic samples showing variant detection capabilities. not applicable for study type.
Randomization	No randomization was performed as we didnt assess phenotypes. not applicable for study type.
Blinding	No blinding was done as we didnt run statistical associations or other studies where this would be needed. not applicable for study type.

Reporting for specific materials, systems and methods

We require information from authors about some types of materials, experimental systems and methods used in many studies. Here, indicate whether each material, system or method listed is relevant to your study. If you are not sure if a list item applies to your research, read the appropriate section before selecting a response.

Materials & experimental systems

n/a	Involved in the study
☒	☐ Antibodies
☒	☐ Eukaryotic cell lines
☒	☐ Palaeontology and archaeology
☒	☐ Animals and other organisms
☒	☐ Clinical data
☒	☐ Dual use research of concern
☒	☐ Plants

Methods

n/a	Involved in the study
☒	☐ ChIP-seq
☒	☐ Flow cytometry
☒	☐ MRI-based neuroimaging
